# Widespread genomic influences on phenotype in Dravet syndrome, a ‘monogenic’ condition

**DOI:** 10.1093/brain/awad111

**Published:** 2023-04-03

**Authors:** Helena Martins Custodio, Lisa M Clayton, Ravishankara Bellampalli, Susanna Pagni, Katri Silvennoinen, Richard Caswell, Genomics England Research Consortium, John C Ambrose, Prabhu Arumugam, Roel Bevers, Marta Bleda, Freya Boardman-Pretty, Christopher R Boustred, Helen Brittain, Matthew A Brown, Mark J Caulfield, Georgia C Chan, Adam Giess, John N Griffin, Angela Hamblin, Shirley Henderson, Tim J P Hubbard, Rob Jackson, Louise J Jones, Dalia Kasperaviciute, Melis Kayikci, Athanasios Kousathanas, Lea Lahnstein, Anna Lakey, Sarah E A Leigh, Ivonne U S Leong, Javier F Lopez, Fiona Maleady-Crowe, Meriel McEntagart, Federico Minneci, Jonathan Mitchell, Loukas Moutsianas, Michael Mueller, Nirupa Murugaesu, Anna C Need, Peter O'Donovan, Chris A Odhams, Christine Patch, Daniel Perez-Gil, Marina B Pereira, John Pullinger, Tahrima Rahim, Augusto Rendon, Tim Rogers, Kevin Savage, Kushmita Sawant, Richard H Scott, Afshan Siddiq, Alexander Sieghart, Samuel C Smith, Alona Sosinsky, Alexander Stuckey, Mélanie Tanguy, Ana Lisa Taylor Tavares, Ellen R A Thomas, Simon R Thompson, Arianna Tucci, Matthew J Welland, Eleanor Williams, Katarzyna Witkowska, Suzanne M Wood, Magdalena Zarowiecki, Andreas Brunklaus, Renzo Guerrini, Bobby P C Koeleman, Johannes R Lemke, Rikke S Møller, Ingrid E Scheffer, Sarah Weckhuysen, Federico Zara, Sameer Zuberi, Karoline Kuchenbaecker, Simona Balestrini, James D Mills, Sanjay M Sisodiya

**Affiliations:** University College London Queen Square Institute of Neurology, Department of Clinical and Experimental Epilepsy, London, WC1N 3BG, UK; Chalfont Centre for Epilepsy, Chalfont St Peter SL9 0RJ, UK; University College London Queen Square Institute of Neurology, Department of Clinical and Experimental Epilepsy, London, WC1N 3BG, UK; Chalfont Centre for Epilepsy, Chalfont St Peter SL9 0RJ, UK; University College London Queen Square Institute of Neurology, Department of Clinical and Experimental Epilepsy, London, WC1N 3BG, UK; Chalfont Centre for Epilepsy, Chalfont St Peter SL9 0RJ, UK; University College London Queen Square Institute of Neurology, Department of Clinical and Experimental Epilepsy, London, WC1N 3BG, UK; Chalfont Centre for Epilepsy, Chalfont St Peter SL9 0RJ, UK; University College London Queen Square Institute of Neurology, Department of Clinical and Experimental Epilepsy, London, WC1N 3BG, UK; Chalfont Centre for Epilepsy, Chalfont St Peter SL9 0RJ, UK; Kuopio Epilepsy Center, Neurocenter, Kuopio University Hospital, Kuopio 70210, Finland; Exeter Genomics Laboratory, Royal Devon University Healthcare NHS Foundation Trust, Exeter EX2 5DW, UK; Paediatric Neuroscience Research Group, Royal Hospital for Children, Glasgow G51 4TF, UK; Institute of Health and Wellbeing, University of Glasgow, Glasgow G12 8TB, UK; Neuroscience Department, Meyer Children’s Hospital IRCSS, University of Florence, 50139 Florence, Italy; Department of Genetics, University Medical Centre Utrecht, 3584CX Utrecht, The Netherlands; Institute of Human Genetics, University of Leipzig Medical Center, Leipzig 04103, Germany; Center for Rare Diseases, University of Leipzig Medical Center, Leipzig 04103, Germany; Department of Epilepsy Genetics and Personalized Medicine, Danish Epilepsy Centre, DK-4293 Dianalund, Denmark; Department of Regional Health Research, University of Southern Denmark, DK-5230 Odense, Denmark; Epilepsy Research Centre, Florey Institute, University of Melbourne, Austin Health and Royal Children's Hospital, Melbourne, VIC 3084, Australia; Murdoch Children's Research Institute, Parkville, VIC 3052, Australia; Applied and Translational Neurogenomics Group, VIB Centre for Molecular Neurology, VIB, Antwerp 2610, Belgium; Translational Neurosciences, Faculty of Medicine and Health Science, University of Antwerp, Antwerp 2650, Belgium; Department of Neurology, University Hospital Antwerp, Antwerp 2650, Belgium; µNEURO Research Centre of Excellence, University of Antwerp, Antwerp 2610, Belgium; Unit of Medical Genetics, IRCCS Istituto Giannina Gaslini, 16147 Genoa, Italy; Department of Neurosciences Rehabilitation, Ophthalmology, Genetics, Maternal and Child Health (DINOGMI), University of Genoa, 16132 Genoa, Italy; Paediatric Neuroscience Research Group, Royal Hospital for Children, Glasgow G51 4TF, UK; Institute of Health and Wellbeing, University of Glasgow, Glasgow G12 8TB, UK; University College London Division of Psychiatry, London W1T 7BN, UK; University College London Queen Square Institute of Neurology, Department of Clinical and Experimental Epilepsy, London, WC1N 3BG, UK; Chalfont Centre for Epilepsy, Chalfont St Peter SL9 0RJ, UK; Neuroscience Department, Meyer Children’s Hospital IRCSS, University of Florence, 50139 Florence, Italy; University College London Queen Square Institute of Neurology, Department of Clinical and Experimental Epilepsy, London, WC1N 3BG, UK; Chalfont Centre for Epilepsy, Chalfont St Peter SL9 0RJ, UK; Amsterdam UMC, University of Amsterdam, Department of (Neuro)Pathology, Amsterdam Neuroscience, 1105 AZ Amsterdam, The Netherlands; University College London Queen Square Institute of Neurology, Department of Clinical and Experimental Epilepsy, London, WC1N 3BG, UK; Chalfont Centre for Epilepsy, Chalfont St Peter SL9 0RJ, UK

**Keywords:** *SCN1A*, Dravet syndrome, polygenic risk scores, blended phenotypes, polymorphism

## Abstract

Dravet syndrome is an archetypal rare severe epilepsy, considered ‘monogenic’, typically caused by loss-of-function *SCN1A* variants. Despite a recognizable core phenotype, its marked phenotypic heterogeneity is incompletely explained by differences in the causal *SCN1A* variant or clinical factors.

In 34 adults with *SCN1A*-related Dravet syndrome, we show additional genomic variation beyond *SCN1A* contributes to phenotype and its diversity, with an excess of rare variants in epilepsy-related genes as a set and examples of blended phenotypes, including one individual with an ultra-rare *DEPDC5* variant and focal cortical dysplasia. The polygenic risk score for intelligence was lower, and for longevity, higher, in Dravet syndrome than in epilepsy controls. The causal, major-effect, *SCN1A* variant may need to act against a broadly compromised genomic background to generate the full Dravet syndrome phenotype, whilst genomic resilience may help to ameliorate the risk of premature mortality in adult Dravet syndrome survivors.

## Teaser

Dravet syndrome is typically considered monogenic, but shows marked phenotypic heterogeneity that is incompletely explained by differences in the causal *SCN1A* variant. Martins Custodio *et al*. now show that additional genomic variation beyond *SCN1A* contributes to and influences Dravet syndrome phenotypes.

## Introduction

With the discovery of numerous monogenic epilepsies, our understanding of the genetic architecture underlying developmental and epileptic encephalopathies (DEEs) has grown immensely.^1^ The initial identification of monogenic epilepsies is usually made through genetic studies of individuals with relatively homogeneous phenotypes. Subsequent characterization of additional cases with pathogenic variants in the same gene typically broadens the phenotypic spectrum.^2,3^ This evolving breadth of clinical presentations, even with a core defining phenotype, can become surprisingly wide and unexplained. One potential source of such phenotypic diversity within a single monogenic epilepsy may be variation across the rest of the genome. This possibility is rarely explored; typically, genetic investigations cease with the discovery of the first plausibly culpable variant.

Pathogenic variants in the voltage-gated sodium channel alpha subunit 1 gene (*SCN1A*) are one of the most frequent causes of monogenic epilepsies, though all are rare.^4^ The archetypal phenotype associated with pathogenic *SCN1A* variants is Dravet syndrome. The spectrum also includes familial febrile seizures, genetic epilepsy with febrile seizures plus (GEFS+), and other *SCN1A-*related epilepsies that do not obviously fit these categories but may share some core features, such as fever-provoked seizures.^5^ Further, people with pathogenic variants in *SCN1A* may also present with features beyond epilepsy, including mild to severe intellectual disability, behavioural problems and movement disorders.^5^ Within *SCN1A*-related conditions, and even for a given pathogenic variant, phenotypic heterogeneity can be observed: a given *SCN1A* variant may segregate with epilepsy in a family, and cause GEFS+ in one individual, and Dravet syndrome in another; individuals meeting a tight clinical definition for Dravet syndrome, harbouring identical *SCN1A* variants, may show divergent phenotypes. This wide range of associated phenotypes confounds prognostication for infants with S*CN1A*-related epilepsies and makes treatment challenging. As a prototypic monogenic disorder, *SCN1A*-related epilepsies provide a model for elucidating the potential contribution of background genetic architecture to the disease phenotype.

Additional genetic factors have been implicated in the phenotypic diversity seen in *SCN1A*-related epilepsies. Disease severity could be modulated by genomic factors directly related to *SCN1A,* such as variant class, mosaicism of the pathogenic *SCN1A* variant, or variants in non-coding regulatory regions affecting the expression of the mutated or wild-type *SCN1A* allele.^6,7^ Alternatively, variants in other genes may influence *SCN1A*-related epilepsy phenotypes, constituting blended phenotypes that reflect an aggregation of distinct or overlapping features, depending on the pathway or function of the gene(s) harbouring the additional variant(s).^8^ The poly-genetic ‘background’ of each individual may act as a phenotypic modifier. Evidence from animal models suggests that genetic background may modulate Dravet-like phenotypes, whilst an enrichment of rare variants in neuronal excitability genes has been reported in severe Dravet syndrome compared to mild Dravet syndrome.^9,10^ Beyond genomic influences, clinical management, including medication choices, may also affect outcomes,^11^ potentially through interactions with individual genetic features.

To test the hypothesis that the background genetic architecture influences the phenotypic presentation of individuals with monogenic epilepsy, we used whole-genome sequencing (WGS) across a cohort of adults with clinically well-characterized *SCN1A*-related Dravet syndrome. We studied several features of background genomic variation, including the contribution of rare variants in epilepsy-related genes, and common variation across the genome, including polygenic risk scores (PRS), aiming to elucidate whether these features influence Dravet syndrome phenotypes.

## Materials and methods

### Ethics statement

This research was approved by the relevant ethics committee. For all cases, written informed consent for research use of clinical and genetic data was obtained from patients, their parents, or legal guardians in the case of those with intellectual disability. All individuals for whom detailed phenotypic information is provided were recruited through a REC-approved study (REC 11/LO/2016), and all phenotypic and genetic information was gathered under this approval.

### Cohort descriptions

#### *SCN1A*-related Dravet syndrome cohort

Thirty-four adults with *SCN1A*-related Dravet syndrome were recruited from epilepsy clinics at the National Hospital for Neurology and Neurosurgery, London, UK through a REC-approved study (REC 11/LO/2016). WGS was performed on DNA extracted from peripheral blood (Supplementary material 1). Detailed clinical phenotyping was undertaken by L.M.C. after comprehensive review of the medical records. The Dravet syndrome phenotype was re-evaluated independently by L.M.C., S.B. and S.M.S. with reference to the diagnostic criteria for Dravet syndrome recently proposed by the International League Against Epilepsy (ILAE)^12^ (Supplementary Table 1 and Supplementary material 2).

The full cohort of 34 individuals with Dravet syndrome was used for the blended phenotype analysis. For PRS and burden analyses, only individuals of European ancestry (28/34) were included (Supplementary Fig. 1 and Supplementary material 3). A cohort including 13 individuals with Dravet syndrome of European ancestry who have missense *SCN1A* variants was used for *post hoc* analyses.

#### Control cohorts

All control cohorts were compiled from participants recruited to the Genomics England (GEL) 100 000 genomes project (Supplementary Fig. 2). Only individuals of European ancestry were considered in the control cohorts (Supplementary Fig. 1 and Supplementary material 3).

#### Genomics England epilepsy controls

The GEL epilepsy control cohort consisted of 772 adults with epilepsy recruited from clinics at the National Hospital for Neurology and Neurosurgery, London, UK, through a REC-approved study (REC 11/LO/2016) and genotyped by the GEL 100 000 genomes project. All individuals fell within the GEL ‘epilepsy and other features’ disease group. The human phenotype ontology (HPO) terms used for these individuals when recruited to the GEL 100 000 genomes project can be found in Supplementary Table 2. To minimize the possibility that individuals within this cohort had *SCN1A*-related epilepsies, individuals with unique variants in *SCN1A* (i.e. not present in gnomAD, version 3.1.1) were excluded (Supplementary Fig. 2).

#### Genomics England controls

The GEL control cohort consisted of 1187 unaffected relatives of probands from GEL disease categories considered to be unrelated to epilepsy (Supplementary Table 3).^13,14^ Medical information regarding these individuals is unknown, and a proportion, likely reflective of the prevalence of active epilepsy in the UK (5–10 per 1000), may have epilepsy, which would serve only to reduce the power of our comparisons. To minimize the number of individuals with potential ‘monogenic’ epilepsies in this cohort, individuals with unique variants (i.e. not present in gnomAD) in epilepsy-related genes were excluded (Supplementary Fig. 2).

#### Genomics England *SCN1A* controls

Following testing of the primary hypotheses, it became clear that a further *post hoc* investigation would be useful, examining individuals bearing ultra-rare *SCN1A* variants, but without epilepsy. The GEL *SCN1A* control cohort consisted of 45 GEL probands of European ancestry [median age at recruitment 37 years (range 4–71)] from disease categories considered to be unrelated to epilepsy (Supplementary Table 3),^13^ who were also identified as having unique/ultra-rare *SCN1A* missense variants (i.e. not present in gnomAD) (Supplementary Fig. 2). No individuals in the disease categories considered to be unrelated to epilepsy had truncating *SCN1A* variants. HPO terms and medical history timelines were reviewed for all identified cases and no individuals were found to have phenotypes that are known to be associated with *SCN1A* variants (Supplementary material 4 and Supplementary Table 4).

#### Epilepsy-related gene selection and annotation

To test the hypothesis that the phenotypic heterogeneity seen in Dravet syndrome could be partly explained by variation in other epilepsy-related genes, in addition to *SCN1A*, samples were screened for rare variants across the canonical coding sequences of 190 monoallelic or X-linked epilepsy-related genes in the GEL Genetic Epilepsy Syndromes (version 2.489) panel (Supplementary Table 5 and Supplementary material 5). Only genes designated by GEL with a ‘green’ rating, (i.e. those in which there is a high level of evidence for gene-disease association), were included and are referred to as ‘epilepsy-related genes’.^13,15^ Rare variants were defined as those with an allele frequency in gnomAD ≤0.0005, which is in line with previously defined ‘rare’ variant allele frequencies.^16,17^ The region of each epilepsy-related gene was extracted from variant call format and annotated using ANNOtate VARiation (ANNOVAR, version 2019Oct24). Stop-gains, frameshift-deletion, frameshift-insertion, in-frame-deletion, in-frame-insertion, splicing, and missense variants with a read coverage ≥8 were selected as qualifying variants. All variants were confirmed manually using the Integrative Genomics Viewer (IGV, version 2.9.4).

#### Gene and gene-set based collapsing analyses of rare variants

An enrichment of rare variants in known epilepsy-related genes confers risk for common and rare epilepsies.^16^ To test the hypothesis that there was an excess of rare variants in epilepsy-related genes in individuals with Dravet syndrome compared with GEL Epilepsy controls, we performed a gene-based and gene-set collapsing analyses for rare variants across 190 epilepsy-related genes.^13,15^ The optimal sequence kernel association test (SKAT-O) as implemented in SKAT R package version 2.0.1 was used.^18^*SCN1A* variants were excluded in both gene-based and gene-set collapsing analyses, to avoid the overestimation of enrichment of rare variants. The variants in these 190 genes were identified using region extraction and Ensembl Variant Effect Predictor (VEP) annotation.^19^ Variants that were observed less than three times in each cohort were included in the SKAT-O analysis. Gender was included as a covariate. A small sample size adjustment by SKAT-O was used. To determine if X chromosome gene variants were driving enrichment of rare variants in Dravet syndrome cases, we performed a rare variant collapsing analysis for the 153 epilepsy-related genes on autosomal chromosomes. To explore whether the burden of rare variants in epilepsy-related genes may influence the expressed phenotype in the setting of a unique *SCN1A* variant, a *post hoc* analysis was performed estimating the gene and gene-set based rare variant enrichment across the Dravet syndrome and GEL *SCN1A* control cohorts.^20^ Bonferroni correction was applied to *P*-values to correct for multiple testing.

#### Blended phenotypes

**Figure 1:**
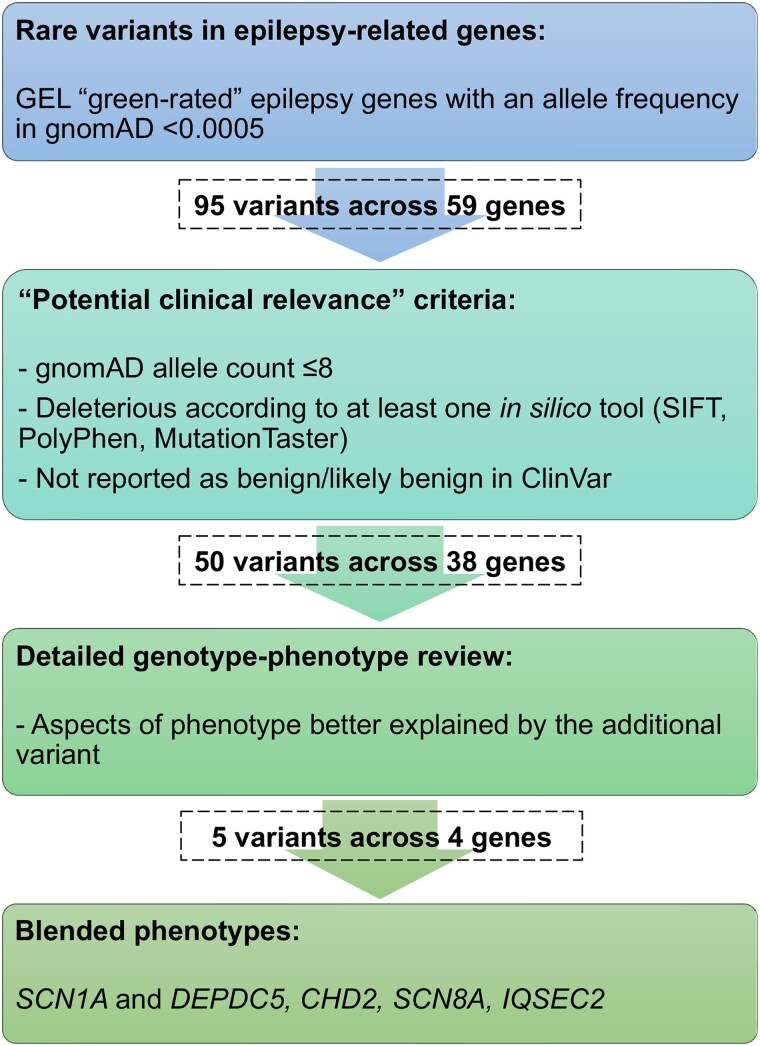
**Method for selection of variants in epilepsy-related genes.** Method for selection of variants in epilepsy-related genes with ‘potential clinical relevance’ that may contribute to blended phenotypes. GEL = Genomics England.

Several large patient series have shown that 3.2–7.2% of those in whom a molecular diagnosis has been identified have multiple molecular diagnoses, i.e. a pathogenic variant at more than one genetic locus, each associated with a distinct clinical disease, and each segregating independently.^8^ Each independent clinical-molecular diagnosis may have distinct or overlapping phenotypic features which together result in a ‘blended phenotype’, representing the complex interaction between effects of pathogenic variants in multiple genes within one individual.^8^ To test the hypothesis that phenotypic heterogeneity could be explained by ‘blended phenotypes’ in some individuals with Dravet syndrome, rare variants in additional epilepsy-related genes were evaluated for ‘potential clinical relevance’ (Fig. 1 and Supplementary material 6). All variants that met the ‘potential clinical relevance’ criteria were evaluated by three clinicians (L.M.C., S.B. and S.M.S.), and the published phenotypes associated with each epilepsy-related gene were compared with the phenotype of the individual harbouring that gene variant, to determine its potential contribution. Additional variants were determined to potentially contribute to blended phenotypes when aspects of the individual’s phenotype were better explained by the additional epilepsy-related gene variant than the *SCN1A* variant (Fig. 1). Variants that were deemed to contribute to blended phenotypes were subsequently classified using American College of Medical Genetics and Genomics-Association for Molecular Pathology (ACMG-AMP) criteria, excluding the criterion ‘BP5 alternate locus observations’ due to the known presence of the *SCN1A* variant,^21^ and were included if they were classified as pathogenic, likely pathogenic or variants of uncertain significance (VUS).

#### PRS

To test the hypothesis that common genetic variation also influences the phenotype, PRS were calculated for epilepsy, intelligence and longevity in the Dravet syndrome, GEL Epilepsy and GEL control cohorts. PRS for intelligence, longevity and epilepsy were estimated using genome-wide association study (GWAS) summary statistics generated by the ILAE Consortium on Complex Epilepsies,^22^ Savage *et al*.^23^ and Deelen *et al*.,^24^ respectively. To investigate the formal genetic correlation between intelligence, longevity and epilepsy, we performed linkage disequilibrium score regression (LDSC) comparing the GWASs used for each PRS estimation (Supplementary Fig. 3). Genetic correlation rates were calculated using the LDSC tool^25^ (Supplementary material 7).

Following quality control steps (Supplementary material 8), we calculated PRS based on the overlap of the study groups’ remaining quality-controlled single nucleotide polymorphisms (SNPs).^26^ PRS for each individual was obtained using the clumping and thresholding method implemented by PRSice-v2.3.3 across a set of *P*-value thresholds (*PT* = 10^−4^, 10^−3^, 10^−2^, 5 × 10^−2^, 10^−1^, 0.5, 1).^27^*PT* with the best fit for the target trait across the thresholds was identified (Supplementary material 9, and Supplementary Figs 4–10). *R*^2^ was used to measure the variance explained by the PRS and was produced directly from PRSice.^27^

To compare PRS between the three cohorts for the selected best-fit *PT*, a one-way ANOVA was applied (Supplementary material 10). The analysis of variance model was adjusted for sex and the first four principal components of ancestry, which further controls for ancestry bias.^28^ Differences in the means between each pair of groups were assessed for significance using a *post hoc* multiple pairwise comparison (Tukey’s test). To correct for multiple testing across three PRS analyses Bonferroni correction was applied to *P*-values and the significance set to *α* = 0.05 / 3.

To further demonstrate that a potentially ‘causal’ *SCN1A* variant is acting against a genomic background that may influence the expressed phenotype, we performed a set of *post hoc* analyses. We estimated the same three PRS across the Dravet syndrome and GEL *SCN1A* control cohorts. Differences in the PRS between cohorts were calculated as above. There is evidence that the most significantly associated SNP from the epilepsy GWAS may exert regulatory control over *SCN1A*^22^ and, therefore, may influence the outcome of PRS for epilepsy in Dravet syndrome. Therefore, we also performed a localized PRS for epilepsy, intelligence and longevity, where we separated out from the GWAS of common epilepsies the genome-wide significant SNPs which mapped to 2q24.3 and corresponded to the *SCN1A*-related locus. Although the 2q24.3 signal consisted of two independent subsignals, as shown in 2018 by the ILAE Consortium on Complex Epilepsies,^22^ the insufficient number of genome-wide significant SNPs corresponding to the two subsignals made performing separate PRS analyses for the two signals impossible; therefore, the genome-wide significant 2q24.3 SNPs across the two regions were considered as a single *SCN1A*-related signal. Localized PRS for epilepsy, intelligence and longevity were performed both for only the 2q24.3 SNPs and excluding the 2q24.3 SNPs and compared across the three cohorts.

### Data and code availability

The data can be requested by emailing the corresponding author. Data will be shared with bona fide researchers after approval of proposals with signed data access agreements as required by, and subject to, institutional and national regulations.

No bespoke code was used for this study. All code used in the manuscript is in the public domain already and has been appropriately referenced.

## Results

### *SCN1A*-related Dravet syndrome cohort and variant description

Thirty-four adults with *SCN1A*-related Dravet syndrome were included; 28 were of European ancestry. Mean age at last follow-up was 32.5 years [standard deviation (SD) ± 13.6; range 16–70]; mean age at genetic diagnosis was 25.8 years (SD ± 15.3; range 3–59); mean age at seizure onset was 6.5 months (SD ± 3.1; range 2–16); 18 (52.9%) were female. Further information is given in Supplementary Table 1.

**Figure 2:**
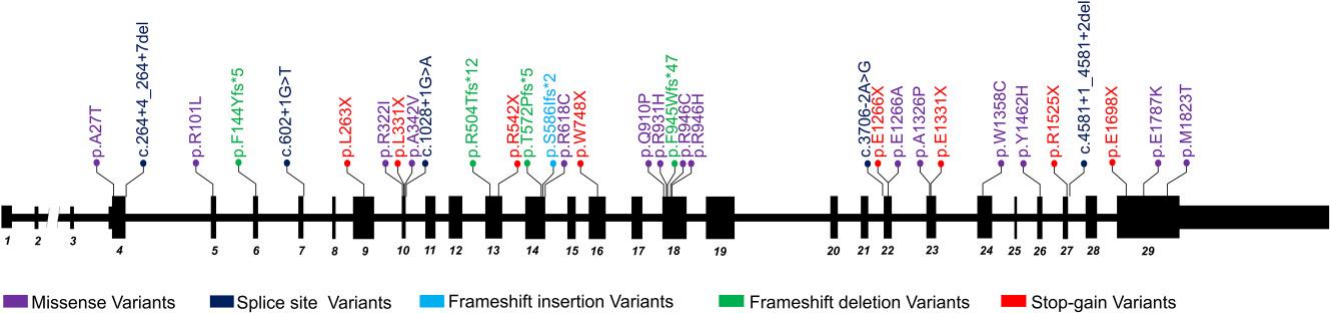
**Distribution of *SCN1A* variants found in the Dravet syndrome cohort.** A schematic diagram of the *SCN1A* gene. Exons are indicated by vertical black boxes (1–29) and introns by the horizontal black line (not to scale). Missense (purple), splicing (dark blue), frameshift insertion (light blue), frameshift deletion (green) and stop-gain (red) variants are shown. The whole gene deletion is not shown. Variants are shown according to the NM_001165963.4 reference sequence.

All pre-identified *SCN1A* variants were validated in the WGS data. Across the 34 individuals, 34 unique *SCN1A* variants were identified including one whole gene deletion. Details of the *SCN1A* variants can be found in Fig. 2, Supplementary material 11, and Supplementary Table 1. The variant distribution is comparable to published cohorts of individuals with *SCN1A*-related syndromes.^4,29,30^ No obvious association between variant class (i.e. missense or null) and specific phenotypes was observed (Supplementary Table 1). In addition, divergent phenotypes were seen in two unrelated individuals (Cases 1-105287 and 1-105683) who shared the same *SCN1A* variant (Supplementary Table 6). The WGS mean read coverage of the *SCN1A* gene region across the samples was 43.5 (excluding the *SCN1A* gene deletion). Visual inspection of the aligned reads using IGV showed an average alternate allele fraction of the known pathogenic *SCN1A* variants of 47.81%, confirming heterozygosity (excluding the homozygous *SCN1A* variant and whole gene deletion). None of the individuals showed evidence for mosaicism of the pathogenic *SCN1A* variant (*P*-value > 0.05; chi-squared test) (Supplementary Table 1 and Supplementary material 12).

We explored whether particular differences between ultra-rare *SCN1A* missense variants identified in the Dravet syndrome and GEL *SCN1A* control cohorts might explain differences in phenotype between these groups. No difference in the *SCN1A*-encoded variant residue location within the protein sequence was seen between missense variants identified in the Dravet syndrome cohort compared with the GEL *SCN1A* control cohort (Supplementary Table 1, Supplementary Table 4 and Supplementary material 13). Five GEL *SCN1A* controls carried *SCN1A* missense variants that have previously been reported in association with epilepsy syndromes, including Dravet syndrome,^31–35^ or sudden unexpected death^36,37^ (Supplementary Table 4).

### Rare variant analyses

#### Collapsing analyses of rare variants: enrichment of rare variants in Dravet syndrome

All individuals with Dravet syndrome were first assessed for the presence of additional rare variants, meeting a frequency cut-off of ≤0.0005 in gnomAD, across 190 epilepsy-related genes: 95 additional rare variants across 59 epilepsy-related genes were identified (Supplementary Table 7). Individuals had a median of 3 (range 0–7; interquartile range 2–3) additional rare variants (Supplementary Table 1).

To evaluate if individuals with Dravet syndrome harbour a higher burden of additional rare variants compared to the control cohorts, we performed gene-based and gene-set collapsing analyses for rare variants across 190 epilepsy-related genes, excluding *SCN1A*.^13,15^ Each gene was considered individually for the gene-based analysis, while all 190 genes were considered as a set for the gene-set collapsing analysis. In the gene-set collapsing analysis, there was an enrichment (*P* = 0.0006) of rare variants in epilepsy-related genes in Dravet syndrome (78 qualifying rare variants in 28 cases; 2.78 variants per individual) compared to the GEL Epilepsy controls (1251 qualifying rare variants in 772 cases; 1.62 variants per individual), in concordance with a previous study reporting an excess of rare variants in (different but overlapping) epilepsy-related genes in individuals with Dravet syndrome.^38^ The gene-based collapsing analyses suggested a higher rare variant burden in the genes *EHMT1*, *CHD2*, *FLNA*, *TSC1*, *PRICKLE1*, *SETBP1*, *NRXN1*, *SPTAN1* and *ARID1B* (*P <* 0.05) in Dravet syndrome compared to GEL Epilepsy controls (Supplementary Fig. 11A), but after correction for multiple comparisons, none of the adjusted *P*-values were significant. Of the 78 rare variants identified in these individuals with Dravet syndrome, a significant proportion (11/78 variants; 14.10%) overlapped with the 1251 rare variants identified in the GEL Epilepsy controls (*P* = 0.0001, Fisher’s exact test). The results of the collapsing analysis for rare variants across 153 autosomal genes showed the same direction of enrichment as in the main analysis for rare variants across all 190 genes (Supplementary material 14). Though we investigated whether the observed variant enrichment in Dravet syndrome was driven by individuals with missense *SCN1A* variants but were underpowered to formally report this outcome (Supplementary material 15 and 16).

#### Rare variants in additional epilepsy-related genes: blended phenotypes and phenotypic heterogeneity

Across all individuals with Dravet syndrome, 51 rare variants in 38 epilepsy-related genes met pre-specified ‘potential clinical relevance’ criteria and underwent a detailed phenotype-genotype review (Supplementary Table 7). Five variants across four epilepsy-related genes (*DEPDC5, CHD2, SCN8A* and *IQSEC2*), all VUS by ACMG-AMP criteria alone, were considered to offer an independent molecular diagnosis, alongside the known *SCN1A* variant, resulting in blended phenotypes including features of both Dravet syndrome and the additional epilepsy-related genetic disorder. Parental samples were not available for these five adults, so we were unable to determine if the additional variants were *de novo.* For each of the five individuals, the variant and phenotype are discussed in detail (see Case 1 below and Supplementary material 17).

#### Case 1: blended phenotype due to *SCN1A* and *DEPDC5* variants (Case id: 1-102398)

**Figure 3:**
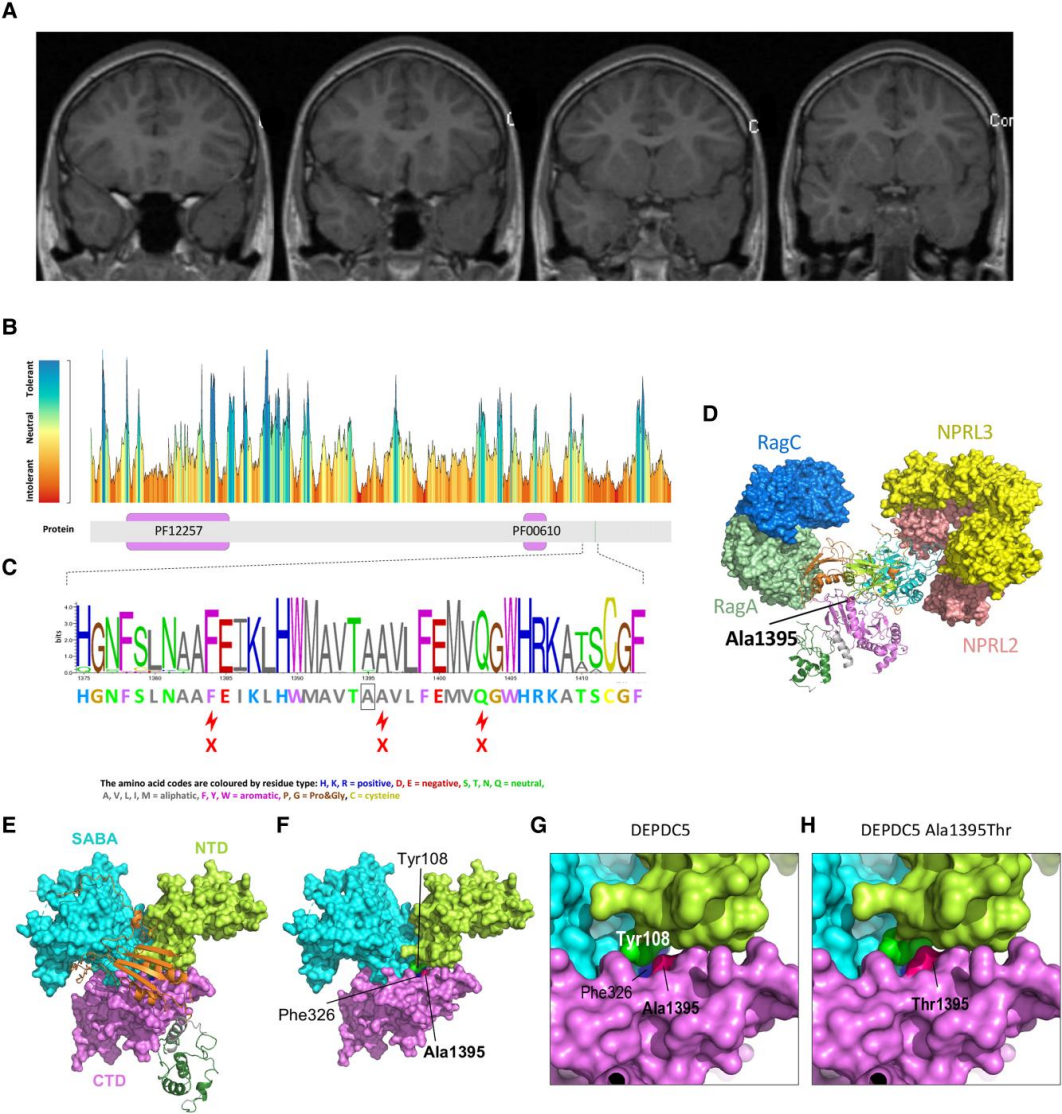
**FCD and details of *DEPDC5* variant.** (**A**) Brain MRI showing FCD. Coronal T_1_-weighted brain MRI from Case 1-102398, with *DEPDC5* variant NM_001242896.3:c.G4183A:p.A1395T, showing left temporal lobe FCD (right of patient is on the *left* of the image, following radiological convention), with blurred grey–white interface and cortical thickening apparent in the left temporal lobe across several consecutive slices. (**B**) MetaDome map of regional constraint in DEPDC5. Grey bar below the graph represents the protein, pink bars showing Pfam domains: PF12257, Vacuolar membrane-associated protein Iml1 domain; PF00610, Domain found in Dishevelled, Egl-10, and Pleckstrin (DEP); A1395 is marked by a vertical green line, with a reported tolerance score of 0.28 (‘intolerant’). (**C**) VarSite sequence logo for DEPDC5 residues 1375–1414, based on alignment of structural homologues; below the logo is the sequence of DEPDC5 itself, with A1395 boxed; sequence conservation score for this residue was 0.92 [range 0 (low)–1 (high)]; alanine was observed at this position in 31/33 aligned sequences. (**D**) Structure of the GATOR1-Rag GTPases complex and context of DEPDC5 Ala1395. PDB 6ces, the structure of the heterotrimeric GATOR1 complex (DEPDC5:NPRL2:NPRL3) bound to RagA and RagC GTPases; protein surfaces shown by colour as indicated (except DEPDC5, shown as a ribbon and coloured by structural domains as annotated by Shen *et al*.^39^: bright green = N-terminal domain (NTD) (residues 38–165); cyan = SABA domain (166–425); orange = steric hindrance for enhancement of nucleotidase activity (SHEN) domain (721–1010); dark green = DEP domain (1175–1270); violet = C-terminal domain (CTD) (1271–1600); Ala1395 is pink with sidechain atoms shown as spheres. (**E** and **F**) Ala1395 lies at an inter-domain interface in DEPDC5. The figure shows selected residues of DEPDC5 from PDB 6ces (chain D); residues of the NTD, SABA domain and CTD are shown as separate surfaces; residues of the SHEN domain and DEP domain are shown as ribbons. **F** shows the same structure as **E** with SHEN and DEP domains removed; residues Tyr108 (bright green), Phe326 (blue) and Ala1395 (rose pink) lie in close proximity at a three-way interface between the NTD, SABA and CTD. (**G**) Enlarged image of the DEPDC5 structure (PDB 6ces, chain D) as in **E** and **F**, zoomed to show detail around the three-way interface between the NTD, SABA and CTD; (**H**) The Ala1395Thr substitution results in reduced space at the inter-domain interface in 6cesD. This figure shows the same structure as **G** after introduction of the Ala1395Thr variant by *in silico* mutagenesis. Quantitative results are given in Supplementary material 18. Analysis of DEPDC5 from PDB 6cet is shown in Supplementary Fig. 1

This individual with Dravet syndrome and a likely pathogenic splicing variant in *SCN1A* (NM_001165963:exon22:c.3706-2A>G), has left temporal lobe focal cortical dysplasia (FCD) (Fig. 3A), and ictal scalp EEG recordings consistently demonstrating that many of his seizures are of left temporal onset (see Supplementary material 17, for full details). He was found to have a *DEPDC5* missense variant (NM_001242896.3:c.G4183A:p.A1395T) that met pre-specified ‘potential clinical relevance’ criteria.

The identified *DEPDC5* missense variant replaces a highly conserved alanine with threonine at codon 1395 of the DEPDC5 protein (Fig. 3B and C), with a Genomic Evolutionary Rate Profiling (GERP) score of 4.1, indicating the site is under evolutionary constraint.^40^ Computational evidence (SIFT, PolyPhen-2, MutationTaster) suggests the variant is damaging (Supplementary Table 7). Whilst most pathogenic variants in *DEPDC5* are truncating, some missense variants are also established as disease-causing, and have been identified in individuals with FCD.^41–44^ This variant is encountered in seven individuals in gnomAD, corresponding to an allele frequency of 0.00005, considered to be within the pathogenic range,^45^ and is absent from an ancestry-matched population database (*n* = 800).^46^ The penetrance of *DEPDC5*-related epilepsies is estimated to be around 60%,^47^ and therefore the presence of this variant at low numbers within a population database would not be unexpected. This variant is considered a VUS according to a classification framework specifically adapted to GATOR1 genes,^48^ by ACMG-AMP criteria, and reported as a VUS in ClinVar. To further explore its potential pathogenicity, *in silico* modelling was undertaken. Ala1395 lies at an internal inter-domain interface between the N-terminal, Structural Axis for Binding Arrangement (SABA) and C-terminal domains of DEPDC5 (domains as defined by Shen *et al*.^39^), in close proximity to residues within those domains (Fig. 3D–G and Supplementary Fig. 12A–C). The effect of the variant was examined in both published structures for DEPDC5, protein data bank (PDB) 6ces (GATOR1 complex bound to Rag GTPases) and 6cet (GATOR1 complex alone), with similar, though not identical, results (for details, see Fig. 3H, Supplementary Fig. 12D and Supplementary material 18). In summary, the Ala1395Thr variant has a deleterious impact either on the folding and/or stability of DEPDC5, or impairs the ability of the GATOR1 complex to respond to Rag GTPases, in both cases likely leading to loss of function, the most commonly recognized mechanism of disease causation associated with *DEPDC5* variants.

FCD is a malformation of cortical development. We explored the potential contribution of the *SCN1A* and *DEPDC5* variants to the FCD by examining the dynamic expression patterns of those genes in the human temporal neocortex. FCD is thought to arise at 8–20 weeks post-conception,^49^ the time frame in which *DEPDC5* has a peak in expression; conversely, at this time expression of *SCN1A* is minimal (Supplementary Fig. 13 and Supplementary material 19). Therefore, the variant in *DEPDC5* is temporally more likely to be causative of the FCD, in keeping with known consequences of *DEPDC5* loss of function variants.^44,50^ However, we acknowledge that this finding is an association only, that is, we do not know and cannot establish when the FCD arose in the individual. Eight individuals with Dravet syndrome and *SCN1A* variants with FCD, six with histopathological confirmation, have been described (Supplementary Table 8).^51–55^ To our knowledge, in these reports, only *SCN1A* sequencing was undertaken.

Overall, in the context of the visualized FCD, concordant electroclinical onset for many of his seizures, the *in silico* analysis and the temporal expression, we consider this variant to likely be contributory, thus potentially responsible for generating a blended phenotype in this individual. To confirm this finding a full exploration with model systems would be required.

### PRS analyses

In Dravet syndrome, phenotypic heterogeneity encompasses many elements, including seizure severity and type, degree of intellectual disability, risk of sudden unexpected death in epilepsy (SUDEP) and comorbidities. Common genetic variation that confers risks for these traits may influence the phenotypic expression. We used two PRS analyses to explore key characteristics of Dravet syndrome for which there is known phenotypic heterogeneity: ‘epilepsy’ and ‘intelligence’. In addition, recognizing that our adult Dravet syndrome cohort represents self-selected survivors, we also performed a PRS for ‘longevity’. All PRS were performed on individuals of European ancestry only.

#### PRS for intelligence: common genetic variation may influence severity of intellectual disability in Dravet syndrome

**Figure 4:**
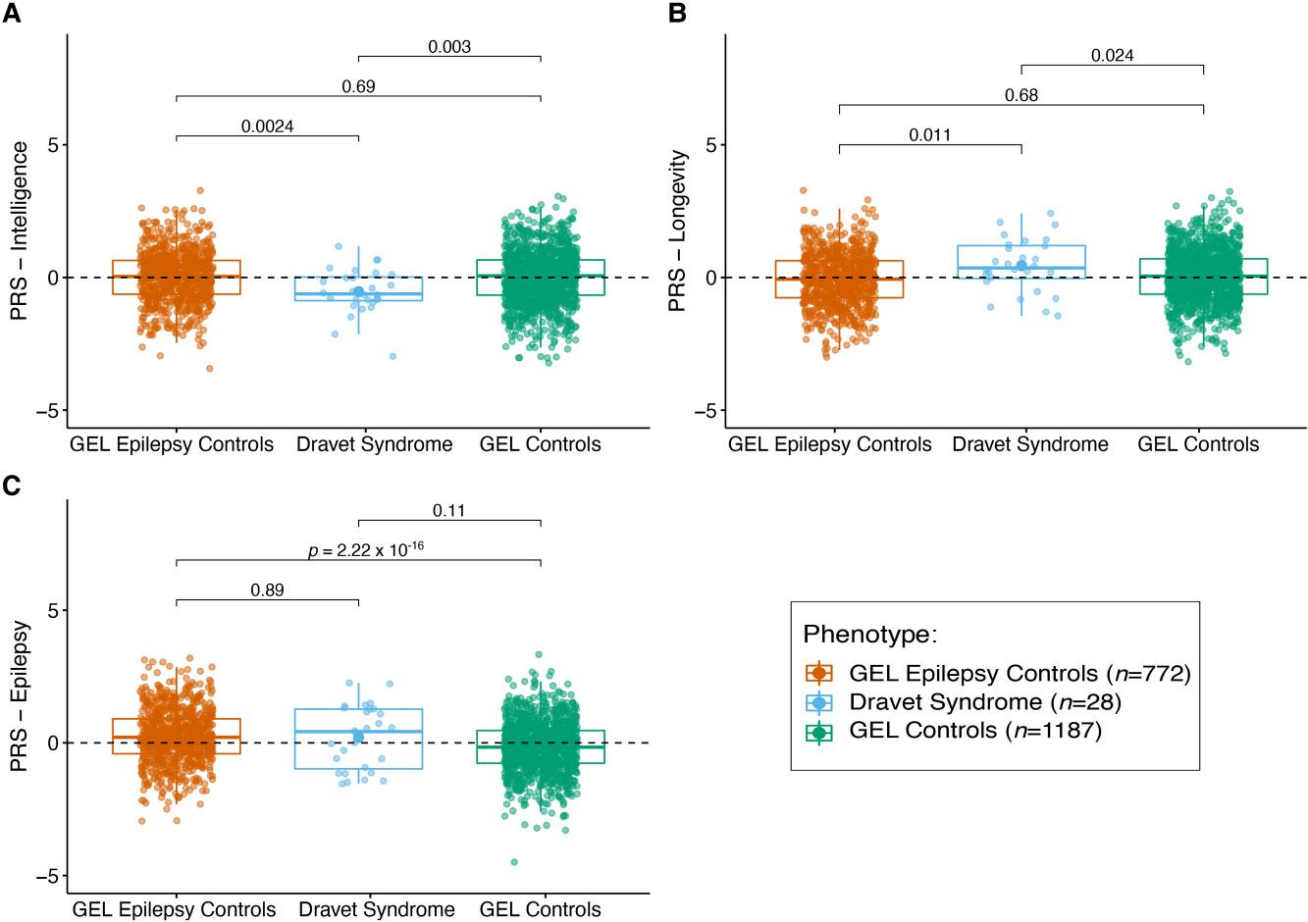
**PRS applied across the cohorts.** (**A**) Polygenic risk score (PRS) for intelligence was lower in the Dravet syndrome cohort than in GEL Epilepsy (adjusted *P* = 0.0024) and GEL control cohorts (adjusted *P* = 0.003). The difference between GEL Epilepsy and GEL controls was not significant (adjusted *P* = 0.69). (**B**) PRS for longevity was significantly higher in the Dravet syndrome cohort than in GEL Epilepsy controls (adjusted *P* = 0.011), and higher than, but not significant, in GEL controls (adjusted *P* = 0.024) and not significantly different in GEL Epilepsy controls compared to GEL controls (adjusted *P* = 0.68). (**C**) PRS for epilepsy was not significantly different in the Dravet syndrome cohort compared with the GEL controls (adjusted *P* = 0.89) and GEL Epilepsy controls (adjusted *P* = 0.11). PRS for epilepsy was significantly higher in the GEL Epilepsy controls than in the GEL controls (adjusted *P <* 2.22 ×10^−16^). The per-PRS *P*-values shown in the graphics are estimated using a *post hoc* multiple pairwise comparison (Tukey’s test). As multiple PRS analyses were performed, the final adjusted *P*-value significance threshold was set to *α* = 0.05/3. GEL = Genomics England.

Intellectual disability is almost universal in adults with Dravet syndrome, but the severity of impairment can range from borderline to severe,^29,56,57^ although, rarely, adults and adolescents with Dravet syndrome have near-normal intellect.^56–58^ Identical *SCN1A* variants can present with a range of cognitive phenotypes even within families.^59^ Factors impacting cognitive outcomes in people with Dravet syndrome are debated.^11,29,57,60–62^ We hypothesized that the common variant load for intelligence would be lower in individuals with Dravet syndrome compared with GEL Epilepsy and GEL controls. PRS for intelligence was significantly lower in the Dravet syndrome cohort than in GEL Epilepsy (adjusted *P* = 0.0024, at *PT* = 10^−4^, Tukey’s test), and GEL controls (adjusted *P* = 0.003, at *PT* = 10^−4^, Tukey’s test). There was no significant difference in the intelligence PRS between GEL epilepsy and GEL controls (adjusted *P* = 0.69, at *PT* = 10^−4^, Tukey’s test) (Fig. 4A, Supplementary material 9, and Supplementary Figs 4 and 5). The intelligence PRS explained approximately 3% (*R*^2^ = 0.03) of the total phenotypic variance in the Dravet syndrome group (derived from PRSice; Supplementary Fig. 6A).

#### PRS for longevity: common genetic variation may contribute to survival in Dravet syndrome

An estimated 10–20% of children with Dravet syndrome die before reaching adulthood, mostly due to SUDEP and status epilepticus.^63,64^ We hypothesized that the longevity PRS would be higher in this cohort of individuals with Dravet syndrome who have survived into adulthood (mean age 32.5 years), especially as many had received a late diagnosis and had unknowingly had what in retrospect was suboptimal antiseizure medication (e.g. sodium channel-blocking medications) (Supplementary Table 1). PRS for longevity was significantly higher in the Dravet syndrome cohort than in GEL Epilepsy controls (adjusted *P* = 0.011, at *PT* = 10^−2^, Tukey’s test), and higher than, but not significant, in GEL controls (adjusted *P* = 0.024, at *PT* = 10^−2^, Tukey’s test). No significant difference was seen in the longevity PRS comparing GEL controls with GEL Epilepsy controls (adjusted *P* = 0.68, at *PT* = 10^−2^, Tukey’s test) (Fig. 4B, Supplementary material 9, and Supplementary Figs 7 and 8). The longevity PRS explained around 2% (*R*^2^ = 0.02) of the total phenotypic variance in the Dravet syndrome cohort (Supplementary Fig. 6B).

#### PRS for epilepsy: no common genetic variant contribution to the epilepsy phenotype in Dravet syndrome

Variants in *SCN1A* are associated with a spectrum of disorders in which the seizure phenotype is variable, from simple, self-remitting febrile seizures at the mild end, to drug-resistant epilepsy in people with Dravet syndrome at the severe end. Even amongst family members segregating one pathogenic *SCN1A* variant, the severity of the seizure phenotype can be wide-ranging, suggesting a contribution of additional genetic variation to the phenotype.^65^ Therefore, we hypothesized that the PRS for epilepsy would be higher in individuals with Dravet syndrome compared to GEL epilepsy and GEL controls. The epilepsy PRS was higher in the Dravet syndrome cohort compared with the GEL epilepsy and GEL controls, although this did not reach statistical significance (adjusted *P* = 0.89, at *PT* = 10^−2^, and adjusted *P* = 0.11, at *PT* = 10^−2^, Tukey’s test, respectively). As expected, the epilepsy PRS was significantly higher in GEL epilepsy compared with GEL controls (adjusted *P <* 2.22 × 10^−16^, at *PT* = 10^−2^, Tukey’s test) (Fig. 4C, Supplementary material 9, and Supplementary Figs 9 and 10). The epilepsy PRS explained around 0.05% (*R*^2^ = 0.0005) of the total phenotypic variance in the Dravet syndrome cohort (Supplementary Fig. 6C).

### *Post hoc* analyses

#### Variation in *SCN1A* does not influence difference in PRS for intelligence and longevity

To further investigate the influence of *SCN1A*-related common variation on the PRS results, we selected the genome-wide significant SNPs from the largest published GWAS of common epilepsies, which mapped to 2q24.3, corresponding to the *SCN1A*-related locus.^22^ We then performed a localized PRS for intelligence, longevity and epilepsy first excluding the 2q24.3 SNPs, and then evaluating only the 2q24.3 SNPs.^22^ Exclusion of the *SCN1A* signal did not modify the findings from the full PRS analysis, confirming that common variation in *SCN1A* is not driving the lower PRS for intelligence and higher PRS for longevity in the Dravet syndrome cohort compared with GEL Epilepsy and GEL control cohorts (Supplementary Fig. 14). PRS performed considering only the 2q24.3 *SCN1A*-related SNPs did not show a significant difference across the cohorts, further supporting the finding that the *SCN1A* signal is not driving differences in PRS (Supplementary Fig. 1^5^).

#### PRS and burden analyses of GEL *SCN1A* control cohort

To further evaluate the hypothesis that additional rare and common genetic variation may be necessary for the Dravet syndrome phenotype in some individuals with *SCN1A* variants, a *post hoc* exploration with PRS and burden analysis was undertaken, comparing individuals with Dravet syndrome with a GEL *SCN1A* control cohort composed of 45 GEL probands with unique *SCN1A* missense variants, but without epilepsy (Supplementary Table 4). Five GEL *SCN1A* controls carried unique *SCN1A* variants that have previously been reported in association with epilepsy syndromes^31–35^ or sudden unexpected death^36^ (Supplementary Table 4).

**Figure 5:**
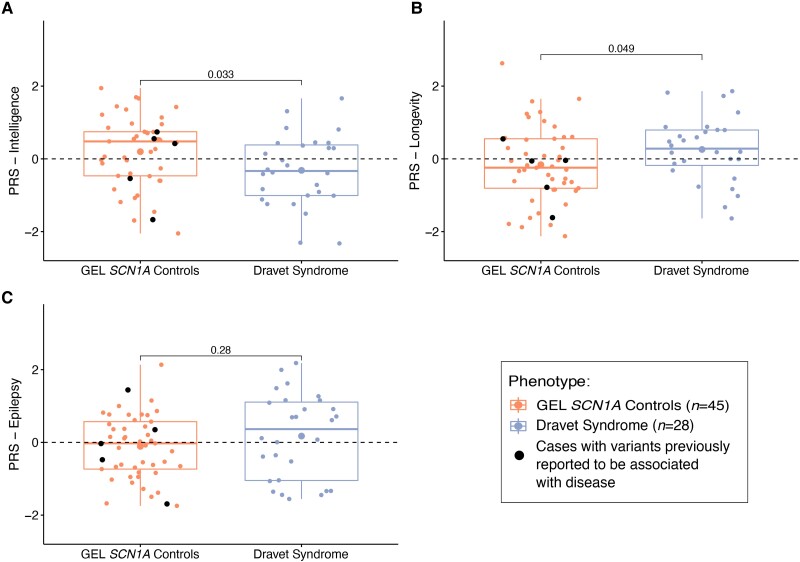
**PRS applied across the GEL *SCN1A* control and Dravet syndrome cohorts.** (**A**) Polygenic risk score (PRS) for intelligence was lower, but not significant, in the Dravet syndrome cohort than in GEL *SCN1A* controls (adjusted *P* = 0.033). (**B**) PRS for longevity was higher, but not significant, in the Dravet syndrome cohort than in GEL *SCN1A* controls (adjusted *P* = 0.049). (**C**) PRS for epilepsy was not significantly different between the Dravet syndrome cohort and GEL *SCN1A* controls (adjusted *P* = 0.28). Black circles = individuals from the GEL *SCN1A* control cohort with variants previously reported to be associated with disease. The per-PRS *P*-values shown in the graphics are estimated using a *post hoc* multiple pairwise comparison (Tukey’s test). As multiple PRS analyses were performed, the adjusted *P*-value significance threshold was set to *α *= 0.05/3. GEL = Genomics England.

PRS for intelligence was lower but not significant (adjusted *P* = 0.033, at *PT* = 10^−4^, Tukey’s test) (Fig. 5A), PRS for longevity was higher but not significant (adjusted *P =* 0.049, at *PT* = 10^−2^, Tukey’s test) (Fig. 5B), and PRS for epilepsy was higher but not significant (adjusted *P* = 0.28, at *PT* = 10^−1^, Tukey’s test) in the Dravet syndrome cohort compared with the GEL *SCN1A* controls (Fig. 5C). We also compared PRS for intelligence, longevity, and epilepsy between GEL *SCN1A* controls and the 13 Dravet syndrome cases with *SCN1A* missense variants. No significant difference was identified, though the direction of effect was maintained in comparison to the main analysis (Supplementary Fig. 16).

The gene-set collapsing analysis revealed an enrichment (*P* = 0.010) of rare variants in Dravet syndrome (78 variants in 28 individuals; 2.78 variants per individual) compared with GEL *SCN1A* controls (81 variants in 45 individuals; 1.8 variants per individual). None of the variants identified in Dravet syndrome overlapped with variants in the GEL *SCN1A* controls. A gene-based collapsing analysis highlighted an increased variant burden in *CHD2*, *FLNA* and *TSC1* (*P <* 0.05) in Dravet syndrome compared with GEL *SCN1A* controls (Supplementary Fig. 11B) that was not significant after correction for multiple comparisons.

## Discussion

Dravet syndrome is the archetypal DEE and amongst the most common of the rare epilepsies.^1,4^ Understanding of Dravet syndrome pathophysiology is amongst the most advanced for any DEE, reflected in the range of targeted therapies now in development.^66–68^ The core phenotype is sufficiently distinct that the diagnosis is usually made clinically, followed by genetic testing anticipating a causal *SCN1A* variant, reflecting the very strong association between phenotype and causal gene. Nevertheless, the currently understood full phenotypic spectrum of Dravet syndrome is very broad, to the extent that in the absence of the telling early clinical history, the diagnosis may be missed clinically, especially in adulthood, and only considered on revelation of a putatively pathogenic *SCN1A* variant.^69^ Moreover, even given the distinct core phenotype, there is marked phenotypic heterogeneity within the syndrome,^30^ which is not fully explained by differences between causal pathogenic variants,^29,70^ and unexplained heterogeneity (not always due to mosaicism) within families segregating one pathogenic variant^65^ and between unrelated individuals carrying the same variant.^71^ ‘Incomplete penetrance’ and ‘variable expressivity’ are useful operational constructs in clinical practice to accommodate such heterogeneity. As with the concept of a ‘syndrome’, the undoubted utility of the terms ‘penetrance’ and ‘expressivity’ presumably reflects their basis in biology and pathophysiology. Some of the heterogeneity captured by these terms is probably due to genetic variation beyond the causal *SCN1A* variant. Digenic, oligogenic, polygenic, dual molecular diagnoses, mutational burden and double-hit contributions to disease phenotypes are well established as concepts.^8^ Discovering real examples in epilepsy is complicated both by the many syndromes and conditions that constitute this umbrella term, and by the known common variant contribution to the epilepsies overall. Controlling for the main genetic contributor of a genetic condition can allow additional genetic contributions to the phenotype to be discovered, as has been shown for example in Huntington’s disease.^72,73^ Here, we adopted the same approach to Dravet syndrome, exploring WGS from a small group of adults with Dravet syndrome due to variation in *SCN1A*. We show that in clinically-distinct cases of Dravet syndrome, with a known *SCN1A* variant (classified as pathogenic or likely pathogenic in 33/34 cases, and published as pathogenic in the remaining case^74^), there are examples of blended phenotypes, an excess of rare variants in epilepsy-related genes, and polygenic contributions to the overall phenotype, with additional evidence for genomic resilience (significantly elevated PRS for longevity). We show that beyond the causal coding or genic *SCN1A* variant, enrichment of rare variants in epilepsy-related genes and common variation in both *SCN1A* and across the genome are present and may have an impact. The presence of two disease-causing rare variants can lead to blended phenotypes, as shown by the presence of symptomatic FCD and a *DEPDC5* variant in one individual with a clear Dravet syndrome phenotype due to a causal variant in *SCN1A*, with additional examples in other genes (*CHD2*, *IQSEC2* and *SCN8A*). PRS analyses demonstrate that the causal *SCN1A* variant is acting against particular backgrounds. The effect size (as demonstrated by the explained variance) is limited, a common observation in studies of polygenic risk using current tools. However, evidence shows that the polygenic background may have a more substantial and clinically relevant effect in individuals with a monogenic disease,^75,76^ demonstrating the principle that the rest of the genome is not inert in monogenic epilepsies, as recently demonstrated in unselected DEEs.^77^

For example, in two unrelated individuals with Dravet syndrome from this cohort, who share the same *SCN1A* splicing variant, the milder seizure and cognitive phenotype in one may in small part be explained by their lower epilepsy, and higher intelligence, PRS, respectively (Supplementary Table 6), demonstrating how a more (or less) favourable genetic background may contribute to explaining intra-familial and variant-specific phenotypic heterogeneity, and have bearing on our understanding of disease biology in ‘monogenic’ epilepsies. Of particular interest, the significantly lowered PRS for intelligence in our cohort could imply that even with symptomatic treatment leading to seizure freedom, or with disease-modifying treatment increasing *SCN1A* expression, the full phenotype of Dravet syndrome may not be entirely reversible. All these additional rare and common variants are obviously present independently of the observed *SCN1A* variant. Our results demonstrate that there is value in exploring additional genomic variation even when a ‘causal’, plausible and compatible pathogenic variant is identified, but clearly challenges remain in such work. Gathering and sequencing a cohort large enough to explore additional genomic variation, such as *SCN1A*-independent common (for example, through a genome-wide SNP-based association study) and rare variation (for example, through gene burden testing) is challenging. Functional validation for multiple variants will be complex, especially when, in most cases, there is no functional validation in clinical practice for the *SCN1A* variant itself found in an individual with Dravet syndrome: individual-based induced programmable stem cells and organoids may offer a way forward.^37^ More tools are being developed that will allow integration and joint analysis of the contributions of different types of variation (e.g. category-wise association studies), but many potentially useful existing tools, especially those devised for clinical application, such as the ACMG-AMP system, are not intended to be used for additional variants^21^: our mindset is still largely centred on monogenic causation.

Nevertheless, we demonstrate that pathogenic variants in *SCN1A* do not necessarily act alone to produce the final phenotype: *SCN1A* may be the gene of major effect in Dravet syndrome, but it is not always the only gene, or only variant, of relevance. Moreover, Dravet syndrome-causing pathogenic variants may need to act against a broadly compromised genomic background (with, for example, a lower PRS for intelligence) to generate the full Dravet syndrome phenotype, whilst on the other hand genomic resilience may ameliorate some serious outcomes, such as premature mortality in Dravet syndrome, as shown by the elevated PRS for longevity in our adult Dravet syndrome survivors, most of whom had received a diagnosis in adulthood, and had been exposed to contraindicated medication. That a causal *SCN1A* variant inevitably acts within the context of the rest of the genome, some variation within which is relevant to the final phenotype, is perhaps unsurprising, but has not been demonstrated across a range of *SCN1A* variants before, and has not been addressed using the range of variation that can be examined using WGS data. Such work may help define the true phenotypic breadth of DS and other ‘monogenic’ conditions, and constrain the often bewildering expansion of phenotype in any given condition. Finally, the revelation of additional influential genomic variation in individual cases may have relevance to individual prognostication, and to treatments currently in development (e.g. gene-based therapies), informing realistic outcomes to be expected from new and existing treatments, and point the way to novel treatments, for example by using information from genomic variants in individuals with mild phenotypes to generate therapies to lessen severity in those with more severe phenotypes.

There are limitations to this study, primarily the limited size of the cohort, the cohort only consisting of adults and the lack of experimental validation using appropriate model systems. Despite these limitations, the results suggest that there may be occasions when stopping at the first plausible causal variant is premature,^8^ with additional biological information of value identifiable by more extensive interrogation of the rest of an individual’s genome. Non-genomic factors will undoubtedly also modulate phenotype, but genomic variation may contribute more than is currently believed.

## Supplementary Material

awad111_Supplementary_Data

## Appendix I: Genomics England Research Consortium contributors

Full details are provided in the Supplementary material.

John C. Ambrose, Prabhu Arumugam, Roel Bevers, Marta Bleda, Freya Boardman-Pretty, Christopher R. Boustred, Helen Brittain, Matthew A. Brown, Mark J. Caulfield, Georgia C. Chan, Adam Giess, John N. Griffin, Angela Hamblin, Shirley Henderson, Tim J. P. Hubbard, Rob Jackson, Louise J. Jones, Dalia Kasperaviciute, Melis Kayikci, Athanasios Kousathanas, Lea Lahnstein, Anna Lakey, Sarah E. A. Leigh, Ivonne U. S. Leong, Javier F. Lopez, Fiona Maleady-Crowe, Meriel McEntagart, Federico Minneci, Jonathan Mitchell, Loukas Moutsianas, Michael Mueller, Nirupa Murugaesu, Anna C. Need, Peter O'Donovan, Chris A. Odhams, Christine Patch, Daniel Perez-Gil, Marina B. Pereira, John Pullinger, Tahrima Rahim, Augusto Rendon, Tim Rogers, Kevin Savage, Kushmita Sawant, Richard H. Scott, Afshan Siddiq, Alexander Sieghart, Samuel C. Smith, Alona Sosinsky, Alexander Stuckey, Mélanie Tanguy, Ana Lisa Taylor Tavares, Ellen R. A. Thomas, Simon R. Thompson, Arianna Tucci, Matthew J. Welland, Eleanor Williams, Katarzyna Witkowska, Suzanne M. Wood, Magdalena Zarowiecki.
