# Supplementary material for: Widespread genomic influences on phenotype in Dravet syndrome, a ‘monogenic’ condition
Source: Brain. 2023 Apr 3;146(9):3885–97. doi: 10.1093/brain/awad111 (PMC10473570; doi:10.1093/brain/awad111)
Supplement: awad111_Supplementary_Data [file awad111_supplementary_data.zip › brain-2022-01408-File007.pdf]

Supplementary Information:

1) **Supplementary Materials**

2) **Supplementary Figures**

3) **Supplementary Tables**

4) **Supplementary References**

Abbreviations: AAF=Alternate allele fraction, ACMG-AMP=American College of Medical Genetics and Genomics-Association for Molecular Pathology, AFR=African, AMR=American, ASD=Autism Spectrum Disorder, ATP=Adenosine triphosphate, B=Benign, BP4=Computational evidence, Supporting evidence of benign, BP5=Alternate locus observation, supporting evidence for benign, USA=United States of America, CTD=C-terminal Domain, D=Damaging, DEE=Developmental and Epileptic Encephalopathies , DEP=Dishevelled, Egl-10, and Pleckstrin, EAS=East Asian, EUR=European, FCD=Focal Cortical Dysplasia, FIAS=Focal Impaired Awareness Seizure, FLNA=Filamin A, GATK=Genome Analysis Tool Kit, GEL=Genomics England, gnomAD=The Genome Aggregation Database, GOF=Gain-of-Function, GRCh38=Genome Reference Consortium Human Build 38, GTCS=Generalised Tonic-Clonic Seizure, GWAS=Genome-Wide Association Study , HET=Heterozygosity, HPO=Human Phenotype Ontology, IBS=Identity by state, ID=Intellectual Disability , ILAE=International League Against Epilepsy, LDSC= Linkage Disequilibrium Score Regression, LMC=Lisa M Clayton, LOF=Loss of function, NDD=Neurodevelopmental disorder, NR=Not reported in publication, NTD=N-terminal domain, P=Probably damaging, PC1=Principal Component 1, PC2=Principal Component 2, PCA=Principal Component Analysis, PCR=Polymerase Chain Reaction, PCW=Postconception weeks, PDB=Protein Data Bank, PEG=Percutaneous endoscopic gastrostomy, pLI=Probability of being loss-of-function intolerant, PM2=Absent or low frequency in control population, moderate evidence for pathogenic, PP2= Missense variant in a gene that has a low rate of benign missense variation, Supporting evidence of pathogenicity ,

PP3=Computational (in silico) data, Supporting evidence for pathogenic, PRS=Polygenic Risk scores, PT=P-value Threshold, RPKM= Reads per kilobase of transcript per Million reads mapped, SABA=Structural Axis for Binding Arrangement , SAS=South Asian, SB=Simona Balestrini, SCN1A=voltage-gated sodium channel alpha subunit 1 gene, SHEN=Steric Hindrance for Enhancement of Nucleotidase Activity, SIFT=Sorting Intolerant From Tolerant, SKAT=SNP-set (Sequence) Kernel Association Test, SKAT-O= The optimal sequence kernel association test, SMS=Sanjay M Sisodiya, SNPs=Single Nucleotide Polymorphisms, T=Tolerated, TCS=Tonic-Clonic Seizure, UK=United Kingdom, VEP=Ensembl Variant Effect Predictor, VUS=Variant of Uncertain Significance, XLID=X-linked intellectual disability, ΔΔG=Change in free energy, WGS=Whole-Genome Sequencing, 2D PCA=Two-dimensional PCA.

1. Supplementary Materials

Supplementary Material 1: Whole genome sequencing and data processing

Whole genome sequencing (WGS) was carried out at Genuity Science (Dublin, Ireland). WGS was undertaken on DNA extracted from peripheral blood of the probands. Samples were prepared using the TruSeq DNA PCR-Free Library Kit (Illumina, San Diego, CA, USA) in accordance with the manufacturer's guidelines. All samples were subject to paired end sequencing of a read-length of 150 nucleotides to 30x coverage using a NovaSeq6000 (Illumina, San Diego, CA, USA). Quality control of the individual FASTQ files was performed using TrimGalore v0.6.3 (Babraham Institute, Babraham, Cambridgeshire, UK). All low-quality nucleotides and contaminating adapter sequences were removed. Reads that were shorter than 100 nucleotides in length or lacking both forward and reverse orientations were excluded from the downstream analysis. Reads passing quality control were aligned to the human reference genome GRCh38 using Burrows-Wheeler Aligner v0.7.17[1], followed by marking of duplicate reads using Picard tools (v2.20.3) and Base Quality Score Recalibration. The resultant binary alignment map files were then processed through the genome analysis tool (GATK v4.1.2) according to the best practices pipeline for identification of variants and copy number variations[2].

Supplementary Material 2: International League Against Epilepsy diagnostic criteria for Dravet syndrome

For the 34 adults with *SCN1A*-related Dravet syndrome, the Dravet syndrome phenotype was re-evaluated independently by LMC, SB and SMS with reference to the diagnostic criteria for Dravet syndrome currently under review by the International League Against Epilepsy (ILAE)[3]. Individuals classified as having Dravet syndrome were required to meet the ILAE proposed "mandatory criteria", whilst not fulfilling any "exclusionary criteria" (Supplementary Table 1). The mandatory criterion of "drug-resistant epilepsy", was interpreted as meaning that at some point in their disease course an individual was regarded as having "drug-resistant epilepsy" according to the ILAE definition[4], but might no longer fulfil this definition, having achieved seizure freedom at the time of inclusion in the study: seizure freedom is reported in some individuals with Dravet syndrome[5,6]. For the purposes of this study, individuals were allowed a maximum of one "alert criterion" (Supplementary Table 1).

Supplementary Material 3: Estimation of ancestries

The ancestry of the individuals in the dataset was determined by comparison with the 1000 Genomes Project reference dataset[7]. Principal component analysis (PCA) of the combined data was used to detect population structure down to the level of the reference dataset. A 2D PCA plot was used to visualise sample ancestry and only samples from our dataset which overlapped with the European-ancestry samples from the 1000 Genomes Project reference dataset were retained for further analysis (Supplementary Fig. 1).

Supplementary Material 4: Genomics England (GEL) *SCN1A* control cohort

The GEL *SCN1A* control cohort consisted of 45 GEL probands of European ancestry from disease categories considered to be unrelated to epilepsy (Supplementary Table 3), who were also identified as having unique *SCN1A* missense variants (i.e. not present in gnomAD) (Supplementary Table 4). *SCN1A* variants in the GEL *SCN1A* control cohort, were identified using region extraction and Ensembl Variant Effect Predictor (VEP)[8] annotation. Forty-seven individuals were identified; all were ≥4 years old at the time of recruitment to GEL. To ensure that no individuals were included for whom we could not exclude a contributory role for the unique

SCN1A variant to their phenotype, the HPO terms were reviewed for the 47 identified cases. Two individuals were excluded from the GEL *SCN1A* control cohort on this basis. To determine if any of the unique *SCN1A* variants identified in the GEL *SCN1A* controls had previously been associated with disease, we searched for each variant across three *SCN1A* databases (The Leiden Open Variation database https://databases.lovd.nl/shared/genes/SCN1A; The Human Gene Mutation Database, http://www.hgmd.cf.ac.uk/ac/gene.php?gene=SCN1A; Institute of Neurosciences Guangzhou Medical University *SCN1A* database, http://scn1a.caae.org.cn/index.php), and ClinVar. Five variants have previously been reported in association with disease, including two reported in people with Dravet syndrome[9,10], two in association with other epilepsy syndromes[11–13], and one associated with sudden unexpected death[14] (Supplementary Table 4).

Supplementary Material 5: Epilepsy-related gene selection and annotation

Only variants in monoallelic or X-linked epilepsy-related genes were included in the rare variant analyses (including burden analysis and blended phenotype analysis). Epilepsy-related genes in which only biallelic variants have been associated with disease were not included in the rare variant analysis, as parental samples were not available for most individuals with Dravet syndrome, and therefore we were unable to accurately determine if two rare variants within one individual were in *cis* or *trans*.

Supplementary Material 6: Selection criteria for variants with potential clinical relevance

To determine whether phenotypic heterogeneity could be explained by "blended phenotypes" in some individuals with Dravet syndrome, rare variants in additional epilepsy-related genes were evaluated against the following criteria, to identify those with "potential clinical relevance": allele count in gnomAD ≤8[15]; at least one *in silico* prediction tool classifying coding missense variants as deleterious (SIFT score ≤0.05[16], PolyPhen score ≥0.908[17] and MutationTaster[18] for missense

variants), or predicting splicing variants to affect splicing (SpliceAI Delta score ≥0.8[19]); absence of a benign/likely benign classification in ClinVar (where reported) (Supplementary Table 7).

Supplementary Material 7: GWAS Correlation

To avoid over-interpretation of the PRS results, we analysed the genetic correlation between intelligence, longevity and epilepsy, estimating formal genetic correlation (*LD*-score) between the GWAS summaries used for the PRS estimations[21,22,31]. Genetic correlation coefficients (*rg*), obtained with LDSC tool[32], showed that intelligence and longevity are weakly correlated (*rg*=0.16), intelligence and epilepsy show a weak negative genetic correlation (*rg*=-0.19), and epilepsy and longevity show negligible genetic correlation (*rg*=-0.03)(Supplementary Fig. 3): nevertheless, from a conservative approach, as three PRS analyses were performed, the overall Adjusted P-value significance threshold was set to α=0.05/3 (0.017).

Supplementary Material 8: SNP quality control

Following the guidelines of Choi et al., additional quality checks were performed in the target and base data[20–22]. We used PLINK 1.92[23] to remove all samples with <0.98 call rate for all single nucleotide polymorphisms (SNPs). Using a subset of uncorrelated SNPs (r^2<0.1 in a sliding window of 100 SNPs per window and shifting the window by 25 SNPs each time), we calculated heterozygosity (HET), identity by state (IBS), represented by $\hat{\pi}$, and gender, and removed: a) HET outliers >5 standard deviations from the median of the whole sample; ii) closely-related individuals from each identified pair, where $\hat{\pi} \geq 0.125$; iii) all samples where sex determined from genotype did not match with the reported gender. All SNPs with <0.95 genotype rate, <0.01 minor allele frequency, or deviation from Hardy-Weinberg equilibrium (with $P<1x10^{-6}$) in samples from any site, were also removed.

Supplementary Material 9: P-value thresholding for polygenic risk score analysis

To identify the optimal P-value threshold (PT) for PRS prediction, we used the software PRSice-v2.3.3[24]. This program permutes the target trait values across the sample of individuals 10000 times, and the PRS analysis is repeated on each set of permuted phenotypes. Thus, for each permutation, the "best-fit PRS" is obtained as that most associated (higher R^2) with the target trait across the range of PTs considered[24]. We chose the PT with the most significant P-value. We estimated PRS for three phenotypes (intelligence, longevity and epilepsy) for three cohorts (Dravet syndrome, GEL Epilepsy control, and GEL controls) as follows, assuming that the biological signal for common variant risk for all analysed phenotypes is the same irrespective of sample status. We calculated PRS using PRSice in a model that included the three cohorts setting GEL controls and GEL Epilepsy controls as controls, and Dravet syndrome as cases. Using this model, the PT with the most significant P-value was 10^{-4} for intelligence PRS and 10^{-2} for longevity PRS and epilepsy PRS (Supplementary Fig. 6).

To confirm that our approach did not force a single PT across the three groups, we repeated the PRS analyses three times applying PRSice in three different case vs control comparisons: Dravet syndrome vs GEL Epilepsy controls, Dravet syndrome vs GEL controls, and GEL Epilepsy controls vs GEL controls (Supplementary Fig. 5, 8 and 10). For all the three models, the PT with the most significant P-value was 10^{-4} in the PRS for intelligence (Supplementary Fig. 5), 10^{-2} in the PRS for longevity (Supplementary Fig. 8) and 10^{-2} in the PRS for epilepsy (Supplementary Fig. 10). The results from this second approach are concordant with those of the first.[25] The methods described above were also applied in the localised PRS analysis.

Supplementary Material 10: Application of ANOVA test

We considered the assumptions for ANOVA testing. The three cohorts (Dravet syndrome, GEL Epilepsy controls and GEL controls) were independent. We checked that each cohort was normally distributed using the Shapiro-Wilk normality test and the homogeneity of variances using the Bartlett test. For each test, all P-values were >0.05.[25]

Supplementary Material 11: *SCN1A* variants in individuals with Dravet syndrome

In the 34 individuals with Dravet syndrome, all pre-identified *SCN1A* variants were validated in the WGS data. 36 *SCN1A* variants were identified, including 34 unique variants: one individual had two different *SCN1A* variants (1-102398), another individual had homozygous variants (1-104484), and two unrelated individuals ($\hat{\pi}$=0.000; Supplementary Material 8) shared the same variant (1-105287 and 1-105683) (Supplementary Table 1 and 6). Twenty-two *SCN1A* variants were classified as "pathogenic" (recurrent variant counted once) and 10 "likely pathogenic" by the American College of Medical Genetics and Genomics–Association for Molecular Pathology (ACMG-AMP) criteria. Two variants were "variants of uncertain significance" (VUS) (Supplementary Table 1) (homozygous variant counted once): one individual (1-102398) harboured both a likely pathogenic variant and a VUS; one previously reported individual[33] (1-104484) had homozygous variants classified VUS, with a typical Dravet syndrome phenotype. While classed as VUS, these variants have previously been published as pathogenic for DS[33]. Variants included 15 missense variants (homozygous variant counted once), eight stop-gains, five splicing variants (recurrent variant counted once), four frameshift deletions, one frameshift insertion, and one whole gene deletion (Fig. 2; Supplementary Table 1). The *SCN1A* whole gene deletion spanned 350kB (chr2:165953001-166303000) and included the first exon of the gene *TTC21B*, the entirety of *SCN1A*-antisense 1 (*SCN1A-AS1*), and exons 8 to 27 of *SCN9A*. No variants were identified in the *SCN1A* poison exon 20N.

Supplementary Material 12: Detection of mosaicism of *SCN1A* variants

To determine whether mosaicism of the *SCN1A* variant was present in the Dravet syndrome cohort, the alternate allele fraction (AAF) and read coverage were examined using Integrative Genomics Viewer (version 2.9.4) (excluding the homozygous variants and the whole gene deletion)[26]. P-values were calculated (Chi-square test) to test the deviation of the AAF from 0.5[26]. The WGS data from an additional individual (not included in the Dravet syndrome cohort), with a known mosaic *SCN1A* variant, was used as a positive control for the analysis.

Supplementary Material 13: Comparison of ultra-rare missense variants identified in Dravet syndrome and GEL *SCN1A* controls

We explored whether particular differences between ultra-rare *SCN1A* missense variants identified in the Dravet syndrome and GEL *SCN1A* control cohorts might explain differences in phenotype between these groups (Figure A, and Supplementary Table 1 and 4). Variant distribution was explored according to location within one of the NaV1.1 homologous domains (highlighted in blue in Figure A), and also according to location within the pore-forming regions (S5 and S6 transmembrane segments and the interconnecting pore loop). All protein domains were defined according to UniProt[34]. There was no statistically significant difference in variant residue location according to phenotype. However, we acknowledge that this is a small sample, and that several protein domains are combined into one group due to the small sample size.

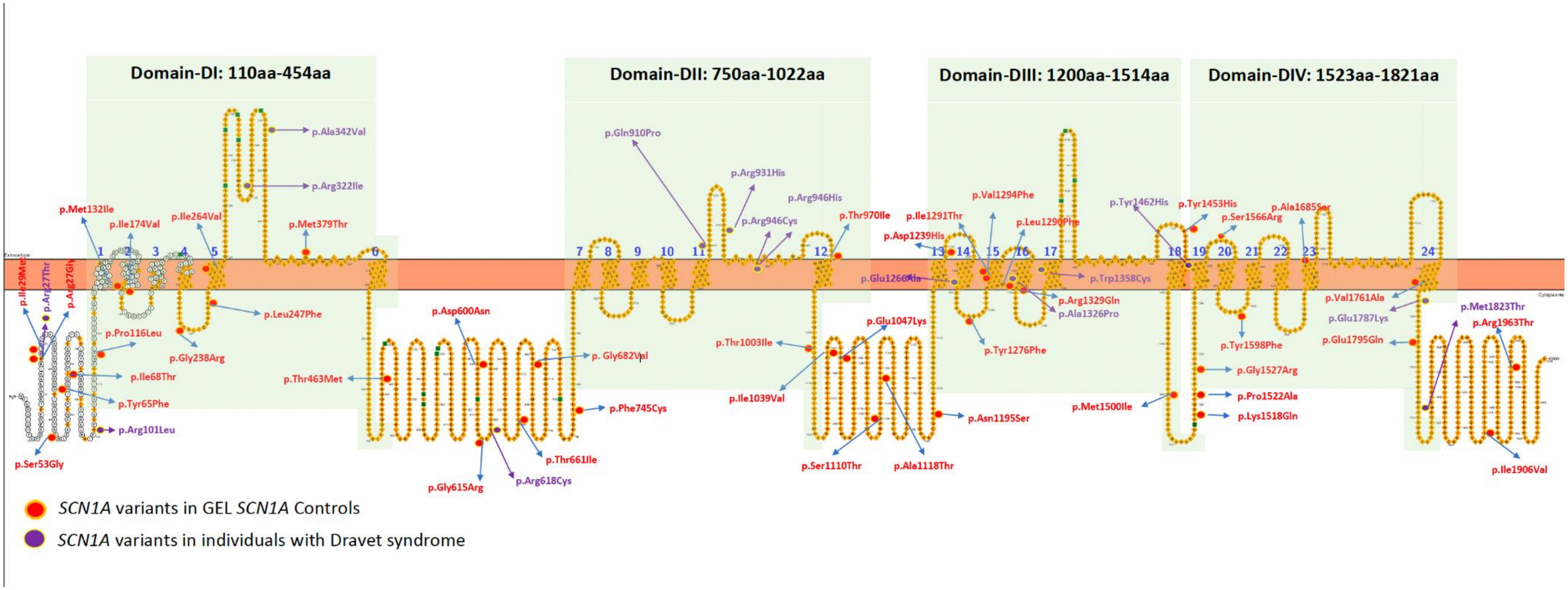

Figure A: Distribution of *SCN1A* missense variants in NaV1.1 protein from the Dravet syndrome and GEL *SCN1A* control cohort: Missense *SCN1A* variants from the Dravet syndrome cohort (purple) and GEL *SCN1A* control cohort (red) are shown on the NaV1.1 protein (Image modified from Protter UniProt protein accession:P35498[35]).

Supplementary Material 14: Gene-set based collapsing analyses: exploring if enrichment of rare variants in Dravet syndrome is driven by rare variants in the X-chromosome.

We performed the rare variant burden analysis across 190 epilepsy-related genes (genes located on both autosomes and X chromosome) in the main analysis. In addition to this, we performed rare variant burden analysis across only 153 epilepsy-related genes present only in the autosomal chromosomes; *SCN1A* gene and 37 X chromosome genes were excluded. We found significant enrichment (*P*=0.0025) of rare variants in Dravet cases (68 qualifying rare variants in 28 cases; 2.43 variants per individual) compared to GEL Epilepsy controls (1108 qualifying rare variants in 772 cases; 1.43 variants per individual). The adjusted P-value was not significant after correction for multiple comparisons. The direction of enrichment was maintained in comparison to the main analysis (rare variants across the 190 genes). These results highlight that the observed enrichment of rare variants across 190 epilepsy related genes in Dravet syndrome cases in the main analysis was not driven by rare variants of X chromosome genes.

Supplementary Material 15: Gene-set based collapsing analyses: exploring if enrichment of rare variants in Dravet syndrome is driven by individuals with missense *SCN1A* variants

To determine whether the observed enrichment of rare variants identified in the Dravet syndrome cohort (*n*=28; patients of European ancestry only), was driven by individuals with missense *SCN1A* variants, we performed the same gene-set collapsing analyses for rare variants across 190 Genomics England (GEL) "green" rated epilepsy-related genes[27,28] only in Dravet syndrome individuals with missense *SCN1A* variants (*n*=13). We performed the optimal sequence kernel association test (SKAT-O) as implemented in SKAT R package version 2.0.1[29]. The rare variants that were observed only in Dravet syndrome individuals with missense variants (*n*=13) and rare

variants in GEL Epilepsy controls (*n*=772) were included in the SKAT-O analysis. A small sample size adjustment by SKAT-O was used. Bonferroni correction was applied to P-values for multiple testing.

Supplementary Material 16: Gene-set based collapsing analyses: Exploring if enrichment of rare variants in Dravet syndrome is driven by individuals with missense *SCN1A* variants

We investigated whether the observed enrichment of rare variants in epilepsy-related genes identified in the Dravet syndrome cohort (*n*=28) was driven by individuals with missense *SCN1A* variants (*n*=13). The SKAT-O analysis was performed for individuals with Dravet syndrome and *SCN1A* missense variants (34 qualifying rare variants in 13 cases; 2.62 variants per individual) and GEL Epilepsy controls (1251 qualifying rare variants in 772 cases; 1.62 variants per individual). This was a post-hoc analysis, and was likely to be underpowered. No significant enrichment was found (*P*=0.055), but the direction of enrichment was maintained in comparison to the main analysis (*n*=28).

Supplementary Material 17: Blended phenotype case reports

Case 1 - Blended phenotype due to *SCN1A* and *DEPDC5* variant (Patient ID: 1-102398)

This 32 year old, right-handed man of non-European descent had a normal birth and early development. At 4.5 months, he experienced a prolonged, hemiclonic febrile seizure, which recurred frequently over the ensuing years. He subsequently developed afebrile tonic, focal, and focal to bilateral tonic-clonic seizures (TCS). From 9 months, development plateaued: he now has severe ID. The genetic diagnosis of Dravet syndrome was made at 23 years. Brain MRI at 15 years showed left temporal lobe focal cortical dysplasia (FCD) (Fig. 3A main text). Fluorodeoxyglucose-positron emission tomography demonstrated focal decreased uptake in the anterior left temporal lobe, indicating localised dysfunction. Multiple electroencephalograms (EEG) over his life show a diffusely slow background with widespread and multifocal interictal sharp waves. Ictal scalp EEG recordings consistently demonstrate that many of his seizures have left temporal onset. His

epilepsy is drug-resistant: he continues to have frequent (~20/month) focal aware and focal impaired awareness seizures (FIAS), characterised by behavioural arrest, facial flushing, stertorous breathing or groaning, clonic twitching of the mouth and cheeks bilaterally and clonic jerking of both arms. In addition, he has 5-10 focal to bilateral TCS per month, mainly arising from sleep. There is no family history of epilepsy.

Further details can be found in the main text.

Case 2 and 3 - Blended phenotypes due to *SCN1A* and *CHD2* variants (Patient ID: 1-103700 (2) and 1-103124 (3)

Rare variants (AF <0.0005) in *CHD2* were identified in 4/34 individuals with Dravet syndrome (Supplementary Table 7). 2/4 *CHD2* variants did not meet the "potential clinical relevance" criteria (Supplementary Table 7) for the blended phenotype analysis; one variant (NM_001271:c.G3400A:p.A1134T) was excluded as the gnomAD allele count was ≥8, and one (NM_001271:c.A4481G:p.N1494S) was excluded as it was classified as benign/tolerated by three *in silico* tools (SIFT, PolyPhen and MutationTaster).

Pathogenicity of the *CHD2* variants: Two *CHD2* variants were classified as VUS by ACMG-AMP criteria (Table A). The NM_001042572:c.G1272T:p.E424D (Patient ID: 1-103700 (case 2)) variant lies within one of two chromodomains within the N-terminal region of *CHD2*[36], which is essential for the chromatin remodelling activity of the encoded protein[37]. The chromodomains are depleted of missense variation in the general population[38]. The NM_001271:c.A4745G:p.E1582G (Patient ID: 1-103124 (case 3)) variant falls within an interdomain region in the C-terminal. The C-terminal region, which contains the DNA-binding domain, is not required for the chromatin remodelling function of CHD2, but enhances its DNA binding and remodelling activity[37]. Pathogenic or likely pathogenic missense variants have previously been reported in this region[38,39], and this variant is predicted to be deleterious by multiple *in silico* tools.

Table A: Two individuals with Dravet syndrome and *CHD2* variants that met "potential clinical relevance" criteria

	CASE 2<br>PATIENT ID: 1-103700	CASE 3<br>PATIENT ID: 1-103124

SCN1A variant:	NM_001353948:c.G3796T: p.E1266X	NM_001353948:c.G2244A: p.W748X
CHD2 Variant:	NM_001042572:c.G1272T: p.E424D	NM_001271:c.A4745G: p.E1582G
ACMG-AMP classification[a]	VUS (PM2 moderate; PP2 supporting; BP4 supporting)	VUS (PM2 moderate; PP3 supporting)
		2
gnomAD allele count	Absent	0.00001
gnomAD allele frequency	0	
Sex / age	Male / 52 years	Female / 23 years
Seizure type at onset (age)	"Minor fits with fever" (9 months)	Prolonged febrile seizure (7 months)
Other seizure types (age of onset)	Unresponsive episodes (unknown, after 9 months)<br>GTCS (unknown, after 9 months)<br>FIAS (unknown, after 9 months)	Unresponsive episodes (12 months)<br>TCS (unknown, after 12 months)
Age at onset of myoclonus	Prior to 9 months	12 months
Age of myoclonic status (recorded on EEG)[b]	26 years	12 years

a= excluding the criterion "BP5 alternate locus observations

b= Other episodes of myoclonic status epilepticus may have occurred but were not confirmed with EEG

GTCS=generalised tonic clonic seizures; EEG=electroencephalogram; ACMG-AMP=American College of Medical Genetics and Genomics/Association for Molecular Pathology American College of Medical Genetics; TCS=tonic clonic seizures (of unknown onset)

Clinical Case 2: The individual is a 52 year old man who was reported previously, before the *CHD2* variant was identified[40]. He was the product of an uneventful pregnancy and birth. At 9 months he had three seizures, described in his paediatric records as "minor fits", at least one of which was associated with fever. He was noted to have prominent myoclonus at the time of presentation and was diagnosed with "myoclonic epilepsy". He subsequently developed frequent GTCS, "absences" (no EEG confirmation) and FIAS.

Myoclonic seizures remained a prominent feature of his epilepsy. In his 20's his myoclonus was described as "almost continuous" and frequent clusters of myoclonic jerks would precede convulsive seizures. He was admitted to hospital aged 26 with myoclonic status, which resolved with the addition of valproate. The withdrawal of carbamazepine aged 28 also led to an improvement in myoclonus.

Later in adulthood, frequent "absences" and monthly GTCS continued. EEGs in adulthood showed encephalopathy with frequent epileptiform discharges. There is no history of photosensitivity and intermittent photic stimulation was not undertaken during any of the available EEG recordings. He is currently treated with valproate, clonazepam, levetiracetam and lacosamide.

His early development was normal but began to slow after seizure onset. Global developmental delay was noted by the age of 3-4 years. Language development was markedly impaired; he was unable to speak in sentences but did use individual words. As a young child he was able to run and ride a pony. At the age of 10 he was noted to have increased tone in all limbs. In his 30's he was found to have a spastic quadriparesis but could take a few steps with assistance. By his 40's he was no longer able to walk and required a wheelchair.

Clinical case 3: The individual is a 23 year old woman. Birth and early development were normal. Seizure onset was at seven months with a prolonged febrile seizure, followed by recurrent febrile seizures every 2-3 months. By 12 months, myoclonic jerks affecting the limbs and neck emerged, as well as unresponsive episodes, and she was started on treatment with valproate. Subsequently, afebrile TCS, of unknown onset, developed.

Development was normal until seizure onset, but then slowed. Neuropsychological assessment at 2.5 years noted language delay, and at 10 years reported severe ID. She has behavioural difficulties.

The frequency of TCS gradually increased through childhood, reaching 100 - 160/year by 15 years, almost exclusively arising from sleep. Through her late teens and early 20's she has continued to have TCS from sleep on most nights, daily myoclonic jerks, and monthly unresponsive episodes. Videotelemetry EEG at 12 years identified frequent myoclonic jerks as well as a period of myoclonic status. Videotelemetry EEG at 23 years showed a slow background, with loss of normal rhythms, and frequent multifocal spike discharges. Frequent, daily myoclonic jerks were captured,

all accompanied by generalised bursts of spike activity. There is no history of photosensitivity and intermittent photic stimulation was not undertaken during any of the available EEG recordings. She is currently treated with a combination of valproate and zonisamide.

Discussion: The two individuals described here were the only individuals in the Dravet syndrome cohort in whom myoclonic status epilepticus was reported. In the first case (2), myoclonic jerks began prior to his first febrile seizure at 9 months. In both cases, myoclonic jerks remained a prominent feature of their epilepsy into adulthood. Myoclonic seizures are infrequently the initial seizure type in Dravet syndrome, more often arising months after epilepsy onset, which is typically with prolonged hemiclonic, or bilateral tonic clonic febrile seizures[41,42]. Conversely, individuals with *CHD2*-related DEE, frequently present with myoclonic seizures, with other seizure types developing concomitantly, or occurring later[36,43]. The prominence of myoclonic seizures during early adulthood, and episodes of myoclonic status, are also unusual for Dravet syndrome. Myoclonic seizures present in childhood often remit by adulthood in people with Dravet syndrome[44–47]. In a case series of adults with Dravet syndrome (aged 20 – 66), myoclonus was present in adulthood in 14/22 individuals and was regarded as "not prominent", although it may be under-reported[48]. Myoclonic status is rare in Dravet syndrome, although it has been reported in some children with a clinical diagnosis of Dravet syndrome, but without genetic confirmation[49,50]. Whilst myoclonic seizures are common in *CHD2*-related DEE, long-term data on their evolution with age are lacking. Episodes of nonconvulsive status epilepticus, often including a prominent myoclonic component, have been described in individuals with *CHD2*-related DEE[51–53].

CHD2 encodes the chromodomain DNA helicase binding protein 2, an ATP-dependent chromatin remodeler that regulates transcription through the manipulation of chromatin structure, and modulation of access for gene regulation machinery [36]. *In vitro* and *in vivo* studies have demonstrated that *CHD2* haploinsufficiency results in dysregulated expression of genes involved in chromatin regulation, neurogenesis, and synaptic organisation[54,55], including a range of genes known to be associated with neurodevelopmental disorders and epilepsy[54,55], such as those encoding sodium and potassium channels[55].

The range of genes for which there may be dysregulated expression as a result of *CHD2* haploinsufficiency might explain the diversity of epilepsy phenotypes that have been associated

with *CHD2*. Syndromes including Dravet syndrome[53], epilepsy with myoclonic-atonic seizures[38,39,51,56], Lennox-Gastaut[39,51,57], West[39], febrile seizures plus[39], epilepsy with eyelid myoclonia[38,58,59,60], childhood absence epilepsy[59], as well as unspecified generalised epilepsy +/- varying degrees of ID, autism spectrum disorder (ASD) and other neurological features[38,52] have all been associated with pathogenic variants in *CHD2.* Whilst *CHD2* is an accepted monogenic epilepsy gene, it is possible that in some individuals *CHD2* may be acting through digenic, oligogenic or modifier mechanisms, alongside other known, or unknown genetic variants, giving rise to the observed phenotypic heterogeneity. Further, the gene-based enrichment analysis demonstrated a greater proportion of rare variants in *CHD2* in Dravet syndrome compared to GEL *SCN1A* controls (14.3% and 0%, respectively), suggesting that *CHD2* may act as a modifier of a co-occurring rare *SCN1A* variant, increasing the risk of developing an epilepsy phenotype, possibly through downstream effects of dysregulated expression of ion channel, and other, genes.

Overall, a contribution of the *CHD2* variant to the phenotype of these individuals cannot be excluded. The *CHD2* variants may contribute independently, resulting in a blended phenotype, representing an aggregation of the effect of both the *CHD2* and *SCN1A* variants. Alternatively, the *CHD2* variants may be acting as modifiers of *SCN1A.* Both proposed mechanisms may lead to a Dravet syndrome phenotype that has features that could be considered “atypical” or “expanded”, such as the early development of myoclonus, and episodes of myoclonic status, both of which are uncommon in Dravet syndrome, and are more typical in *CHD2*-related DEE. Overall, we conclude that these individuals’ phenotypes represent a blended effect of both the *SCN1A* and *CHD2* variants.

Case 4 - Blended phenotype *IQSEC2* and *SCN1A* (Patient ID: 1-106026)

***SCN1A* variant:** NM_006920.6:c.4548+2T>C

Additional Variant: NM_001111125:c.C1448T:p.P483L

Clinical Case: The individual is a 21 year old man. At 5 months he experienced his first prolonged febrile seizure, with the second occurring four months later. From this time, he developed afebrile TCS (of unknown onset) that increased in frequency until the age of 2 years when they were

occurring daily. Other seizure types, including FIAS, myoclonic jerks, atonic and tonic seizures, subsequently developed. Episodes of convulsive and non-convulsive status throughout his teenage years necessitated multiple hospital admissions. Interictal EEG at seizure onset aged 5 months was normal, but from 2 years old showed centrotemporal spikes, and bursts of slow spike and wave independently in both hemispheres.

He had normal early development and by 2 years he could say 50 words, and some short sentences. Developmental regression began at 2 years and by 4 years he became non-verbal. He subsequently lost motor skills, being able to ride a tricycle in infancy, but not by the age of 6 years.

Currently, in his twenties he has daily tonic and TCS, mostly occurring from sleep. He takes brivaracetam, valproate and clobazam. Trials with cannabidiol and stiripentol were both ineffective. He has severe ID and severe ASD. There is no significant family history.

Pathogenicity of the *IQSEC2* variant: The *IQSEC2* variant was considered a VUS according to ACMG-AMP criteria (PM2 moderate; PP2 supporting; PP3 supporting), excluding the criterion “BP5 alternate locus observations”; The variant identified in *IQSEC2* in this case is absent from gnomAD. *IQSEC2* is intolerant to missense variation and loss-of-function (gnomAD missense *Z-Score* 5.19 and pLI = 1, respectively), and a highly restricted level of tolerated variation is observed, particularly in hemizygous males[61].

In males, a range of variants in *IQSEC2*, including truncating, splicing, missense and in-frame deletions, are associated with disease[62]. Variants are mostly *de novo,* although maternally inherited missense variants have been reported, and may be more frequently observed in males than *de novo* missense variants[62]. Whilst pathogenic truncating variants, splicing variants and inframe deletions are distributed throughout the gene, pathogenic missense variants in *IQSEC2* largely cluster within the IQ calmodulin-binding motif, Sec7 and Pleckstrin homology domains, and missense variants within these domains have been shown to result in a partial loss of IQSEC2 function[61,63–65].

The variant identified in this case (c.C1448T:p.P483L) falls outside the four known functional protein domains; however, multiple lines of computational evidence support a deleterious effect on the gene product (Supplementary Table 7). Seven males have been reported in the literature with missense variants in *IQSEC2* falling outside a known functional domain (Table B)[62,66–68]. Piton et al. describe an individual with ASD, with a maternally-inherited variant (R358Q), which

is absent from gnomAD. The variant was predicted to be benign by three *in silico* tools. Further clinical details regarding the proband and mother were not available[67]. Karaca et al. reported an individual with the variant p.R1122C. This variant is present in gnomAD, including in 5 hemizygous males, and has conflicting interpretations of pathogenicity reported in ClinVar. The individual was described as having "dysgenesis of the corpus callosum", but no other clinical features were provided[66]. Three missense variants were identified by Hu et al. in individuals with X-linked intellectual disability (XLID) but without further phenotypic details. Two of the variants are absent from gnomAD, and one is predicted to be pathogenic by two *in silico* tools. The potential pathogenicity of these *IQSEC2* variants, and the relationship to the XLID in these cases, was not further discussed in the reporting study[68]. Mignot et al. describe two related males with moderate to severe ID without epilepsy[62]. The variant was inherited from their unaffected mother and was predicted to be deleterious by two *in silico* tools, and classified as a VUS by ACMG-AMP criteria

Table B: Hemizygous missense variants in *IQSEC2* reported in the literature that do not fall within functional domains

Variant	Phenotype	gnomAD allele count (frequency) total	gnomAD allele count in hemizygous males	Pathogenicity predictions from *in silico* tools (as reported in reporting publication)	Reference
NM_015075.2:c.G1073A:p.R358Q[a]	ASD	0 (0)	0	Polyphen - B<br>SIFT - T<br>Panther - B	Piton 2011[67]
NM_001111125: c.C3364T: p.R1122C[b]	Dysgenesis of the corpus callosum	12 (0.00011)	5	NR	Karaca 2015[66]
NM_015075.2: c.C1751T: p.A584V[c]	XLID	0 (0)	0	Polyphen - P<br>SIFT – D	Hu 2016[68]
NM_001111125: c.C3541T: p.P1181S[c]	XLID	7 (0.00006)	2	Polyphen - P<br>SIFT – T	Hu 2016[68]
NM_001111125: c.C3463T: p.R1155W[c]	XLID	0 (0)	0	NR	Hu 2016[68]
NM_001111125: c.C3463T: p.R1155W[d]	XLID	0 (0)	0	Polyphen - P<br>SIFT – D	Mignot 2019[62]

NM_001111125: c.C3463T: p.R1155W[d]	XLID	0 (0)	0	Polyphen - P SIFT – D	Mignot 2019[62]

ASD – autism spectrum disorder; XLID – X-linked intellectual disability; NR – not reported in publication; P = probably damaging; D = damaging; T = tolerated; B= benign

a - Suspected to be pathogenic by reporting paper

b - Identified as a novel variant in a known gene and representing phenotype expansion

c - Pathogenicity not discussed in the reporting paper

d - Related individuals

Discussion: The case reported here has features typical of Dravet syndrome, including normal early development and language acquisition, followed by significant developmental regression as seizure frequency increased, with eventual severe ID and no verbal communication. ID is almost ubiquitous in adults with Dravet syndrome, but the severity of impairment can range from borderline to profound[41,44,47,69,70], and rare cases of adults and adolescents with Dravet syndrome and *SCN1A* mutations, but normal intellect have also been described[6,44,70]. This individual also has severe ASD. ASD is a common comorbidity in people with Dravet syndrome, the prevalence of which was found to range from 22 - 61.5%, and is significantly higher than that encountered in the general population[69,71,72]. An association between ASD and ID has been reported in individuals with Dravet syndrome, where those with ASD show more significant cognitive impairment than those without ASD[72].

Variants in *IQSEC2* are associated with XLID with and without epilepsy[61]. All described individuals with *IQSEC2*-related encephalopathy show developmental delay and ID which ranges from mild to profound, and ASD is common[61,62,73,74]. In individuals with epilepsy, global developmental delay is evident prior to seizure onset, which is typically at 2 -3 years, with multiple, drug-resistant seizure types[61,62,73,74].

The individual reported in this case had no verbal communication. Most individuals with Dravet syndrome develop some language, but there are typically pronounced deficits, including expressive language dysfunction, dysarthria and oral motor impairment[72,75]. Complete loss of acquired language, rendering an individual non-verbal is reported in Dravet syndrome, although the prevalence of this is unknown[45,47]. Males with *IQSEC2*-related encephalopathies typically have

severe ID, in particular in regard to language development, with many being non-verbal.[62,73,74]. In terms of his other phenotypic features, polymorphic, drug-resistant seizures including generalised tonic-clonic, atypical absences, tonic, atonic and myoclonic seizures, with fever being a common trigger, are encountered in both Dravet syndrome and *IQSEC2*-related encephalopathies[62].

The factors influencing poor cognitive outcomes in people with Dravet syndrome are debated, and do not appear to be related to the *SCN1A* variant type[41,45,76]. Similarly, a genotype-phenotype relationship between *SCN1A* variant type and the development of ASD has not been identified, and factors associated with the co-occurrence of ASD in Dravet syndrome are unknown[71]. It is possible that in individuals with Dravet syndrome (or indeed other monogenic epilepsy), genomic variation beyond *SCN1A* may influence the severity of ID and the development of ASD. In this individual, the *IQSEC2* variant, independently from the *SCN1A* variant, could have resulted in a neurodevelopmental disorder, causing ID, ASD and seizures. In combination with the *SCN1A* variant, the *IQSEC2* variant has resulted in a blended phenotype, that manifests as someone with "severe" Dravet syndrome.

Case 5 Blended phenotype *SCN8A* and *SCN1A* (Patient ID: 1-104181)

***SCN1A* variant:** NM_001353948:c.1511_1515del:p.R504Tfs*12

Additional Variant: NM_014191.4:c.C5098G:p.Q1700E

Clinical Case: The individual is a 23 year old man. He was the product of a normal pregnancy and birth. He experienced his first hemiclonic seizure at 3 months old: it was not associated with fever. He continued to experience afebrile hemiclonic seizures, that would alternate sides, and that could progress to bilateral TCS, from that time. His seizures were refractory to multiple antiseizure medications. Sodium channel-blocking agents were not found to exacerbate his seizures, but were also not found to be effective. He is now on a combination of valproate, stiripentol and clobazam. His early development was normal; he walked at 11 months and said his first words at 1 year. At 15 months he had a prolonged seizure, lasting for more than three hours, after which there was significant developmental regression. Other seizure types including TCS (of unknown onset),

myoclonic jerks and “blank spells”, subsequently also developed. No EEGs were available for review.

Currently, he has 1-2 TCS per week; he has severe ID and ASD. In his late teens, in conjunction with starting stiripentol, he experienced a period of anorexia with significant weight loss. Extensive investigations did not reveal a cause. A percutaneous endoscopic gastrostomy (PEG) was sited to support nutritional intake and medication administration. He has a crouched and ataxic gait, with lower limb pyramidal signs, and has been wheelchair-dependent since his teens. He has microcephaly.

Pathogenicity of the *SCN8A* variant: The *SCN8A* variant identified in this individual is considered a VUS according to ACMG-AMP criteria (PM2 moderate; PM1 supporting; PP2 supporting; PP3 supporting), excluding the criterion “BP5 alternate locus observations”. It is absent from gnomAD. *SCN8A* is highly intolerant to missense variation (gnomAD missense z score of 7.64), which accounts for the majority of disease-causing variation in *SCN8A*[77,78]. The variant lies within a pore-loop between the 5th and 6th α-helical transmembrane segments of the IV domain, which together form the ion-conducting pore of voltage-gated sodium channels[79]. This region exhibits high conservation between species[80]. Pathogenic variants in the pore-loops have been described in individuals with a range of *SCN8A*-related neurodevelopmental disorders (Table C, Figure B)[12,77,81–84]. An unpublished, pathogenic variant (NM_014191:c.C5108A;p.T1703N) in a nearby residue to the variant in this case, is also reported in the SCN8A.net database.

Table C: Individuals reported in the literature with pathogenic *SCN8A* variants falling within the pore-loops

Variant	Phenotype classification[77]	Reference
NM_001330260:c.1078_1079delTTinsGC:p.F360A	DEE	Rolvien 2017[81]

NM_014191: c.A1099G:p.M367V	Insufficient details in reporting study	Encinas 2019[82]
	Insufficient details in reporting study	Lindy 2018[12]
NM_014191:c.C1122G:p.N374K[b]	Intermediate epilepsy[a]	Johannesen 2019[83]
NM_014191:c.G2792A:p.R931Q	NDD without epilepsy[c] (two unrelated individuals)	Johannesen 2021[77]
	Generalised epilepsy[d] (one individual)	
NM_014191:c.G2806A:p.E936K	Intermediate epilepsy[a]	Johannesen 2019[83]
NM_001330260:c.C4214A: p.A1405D	Intermediate epilepsy[a]	Jain 2017[84]

DEE = developmental and epileptic encephalopathy; NDD = neurodevelopmental disorder

Phenotype classification was taken from Johannesen et al.[77]

a - "Intermediate epilepsy" was defined in the citing reference as individuals with a focal epilepsy that was of "intermediate severity", with normal cognition - moderate ID and partially pharmacoresponsive epilepsy. Other features such as autism spectrum disorder, ataxia and behavioural difficulties may also be present[77,83]

b - in vitro studies show mild GOF effects of this variant but without alteration in neuronal firing and the functional consequences were therefore considered to be "neutral"[77]

c - "NDD without epilepsy" was defined in the citing reference as individuals with intellectual disability and/or behavioural disorder, developmental delay, microcephaly[77]

d - "Generalised epilepsy" was defined in the citing reference as individuals with a generalised epilepsy (seizure types and EEG), with cognition ranging from normal to severe ID +/- the presence of additional features including ataxia, behavioural disorders and speech delay[77]

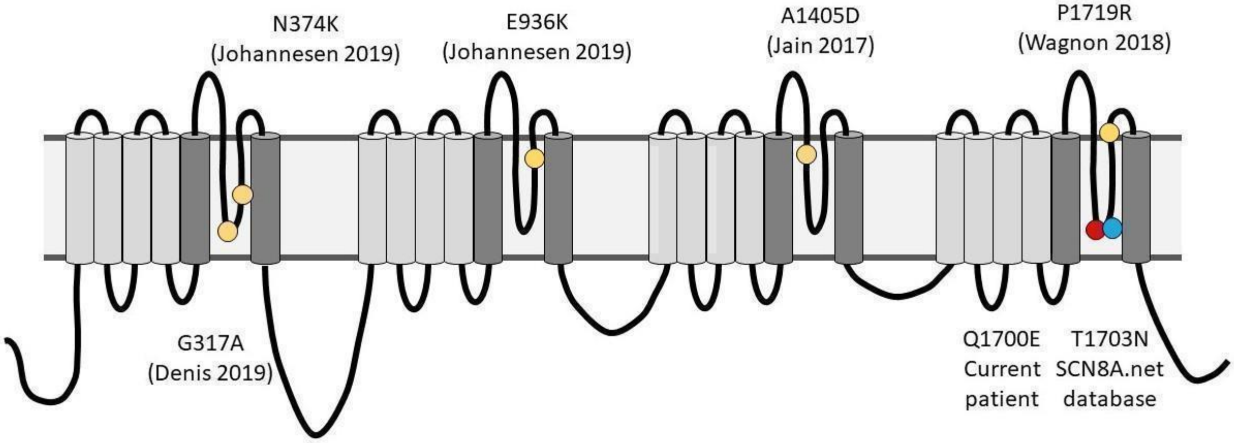

Figure B: *SCN8A*-encoded protein structure showing pathogenic variants falling within the pore loops that, together with the S5 and S6 segments, form the channel pore. Light grey transmembrane segments (S1-4) form the voltage-sensing domains, dark grey transmembrane segments (S5-6), and the interconnecting pore-loops, form the pore forming domains. Yellow circles = pathogenic variants reported in the literature; Red circle = Current case's variant (case report 5; Patient ID: 1-104181); Blue circle = unpublished pathogenic variant reported in SCN8A.net database.

Discussion: The individual described in this case has a core phenotype consistent with Dravet syndrome. There are some atypical features, including the absence of febrile seizures at onset and lack of fever sensitivity, and the presence of microcephaly, which is not described in Dravet syndrome, but has been reported in individuals with *SCN1A*-related early infantile epileptic encephalopathy, secondary to the recurrent Thr226Met variant (not found on WGS in this patient).[85]

The disease trajectory is also consistent with Dravet syndrome, albeit on the severe end of the spectrum, with walking difficulties, spasticity and a crouched gait developing in his teenage years, necessitating the use of a wheelchair by 19 years. Feeding difficulties and weight loss are frequently reported in individuals with Dravet syndrome, and may be exacerbated by use of medications such as topiramate, cannabidiol and stiripentol[86–90]. In our own cohort of adults with Dravet syndrome, PEG insertion for supplemental nutrition due to reduced food intake, or because

of dysphagia, is observed in around 20% (Clayton, unpublished). The average age of insertion is at 31 years, and this individual represents one of the youngest cases requiring gastrostomy in our adult cohort (Clayton, unpublished).

The *SCN8A* variant identified in this individual with Dravet syndrome may explain some of the atypical features, and the severity of his phenotype. *SCN8A*-related syndromes encompass a spectrum of neurodevelopmental disorders, for which the phenotypic heterogeneity can partly be explained by the functional effect of the pathogenic variant[77,78] .

This individual has a previously undescribed missense variant in a pore forming domain. Missense variants falling within pore domains typically result in loss of function (LOF)[77,91], as demonstrated by *in vitro* studies[92–94], or as predicted by a machine learning-based statistical model developed to predict the functional effects of variants in voltage-gated sodium channels[95]. Therefore, it is probable that this individual's *SCN8A* variant results in LOF.

SCN8A LOF variants (either truncating, or missense with *in vitro* evidence of LOF), and missense variants in the pore forming domain with unknown functional effects, are most commonly associated with either a phenotype characterised by generalised epilepsy (with absence, GTCS and febrile seizures), with or without ID, ASD and ataxia, or a neurodevelopmental disorder without epilepsy[77]. Individuals with a *SCN8A*-related neurodevelopmental disorder without epilepsy show mild to severe ID (80%), ASD or attention deficit hyperactivity disorder (43%), speech delay (24%) and/or microcephaly (19%).

SCN8A-related syndromes associated with LOF variants share many clinical features with Dravet syndrome, including intellectual disability, epilepsy and ASD. Disentangling the contribution of each variant is challenging, and the resulting phenotype likely reflects the combined effect of both variants, and may explain the clinical severity of this individual's syndrome with frequent TCS, severe ID and severe ASD. The presence of microcephaly in this case, is more likely to be a consequence of the *SCN8A* variant; microcephaly is reported in a range of *SCN8A*-related syndromes[77,96–99], and is described in 19% of individuals with *SCN8A*-associated neurodevelopmental disorder without epilepsy (associated with LOF variants)[77].

Establishing the role of additional genetic variants in individuals with Dravet syndrome may help to explain phenotypic heterogeneity, and the origin of atypical features. Prior to undergoing WGS,

the presence of microcephaly in this individual could have been interpreted as representing "phenotypic expansion" of Dravet syndrome. However, identification of the additional *SCN8A* variant suggests that his phenotype represents a composite effect of both the *SCN1A* and *SCN8A* variants, with some features that could be attributable to either variant (seizures, ID, and ASD), and others features that are more clearly attributable to only one of the two variants (microcephaly with *SCN8A*).

In animal models, interactions between multiple ion channel variants alter the seizure and behavioural phenotype[100]. For example, in a mouse model of Dravet syndrome (*Scn1a*-haploinsufficient), the introduction of a heterozygous LOF *Scn8a* mutation restored normal seizure thresholds, and improved survival[101]. Similarly, treatment of Dravet syndrome mice with an *SCN8A* antisense oligonucleotide, (leading to a 50% reduction of *Scn8a* transcript in the brain), reduced seizures and prolonged survival[102]. Whilst animal models demonstrate the principle that multiple ion channel variants can interact to result in unique phenotypes in "monogenic" disorders[100], the beneficial effect of *Scn8a* down-regulation/LOF in a *Scn1a*-haploinsufficient Dravet syndrome mouse model, is discordant with what we observe in this individual, whose phenotype is severe with additional features (microcephaly). Further work is required to understand the impact and interaction of rare and common genetic variation across multiple ion channel genes, the functional effects, and the resulting consequences on neuronal function and ultimately disease phenotype.

Supplementary Material 18: The Ala1395Thr variant likely leads to loss of function of DEPDC5, as demonstrated by *in silico* modelling

The effect of the variant was examined in both published structures for DEPDC5, PDB 6ces (GATOR1 complex bound to Rag GTPases) and 6cet (GATOR1 complex alone), with similar, though not identical, results. In both structures, the most obvious impact of the variant is to restrict space at the interdomain interface (Fig. 3G and H, main text), and Supplementary Fig. 12C and D), and to create a novel inter-domain hydrogen bond. In both structures, the net thermodynamic effect of the variant was neutral: in 6ces chain D, the change in free energy due to the variant, $\Delta\Delta G$, is -0.57 kcal/mol, while in PDB 6cetD, the value was -0.28 kcal/mol. Values <1 kcal/mol are generally regarded as benign, suggesting that the variant is unlikely to cause structural

destabilisation *per se*. However, these values for $\Delta\Delta G$ represent only a net effect, and it is important to note that the variant is predicted to alter the manner in which the different domains of the protein interact, with either an impact on folding of the domains into the final 3D conformation, or altered response to Rag GTPase binding through subtle conformational changes. While the structures of DEPDC5 are highly similar in PDB 6ces and 6cet, they are not identical; it is not clear whether these differences are a result of binding of the GATOR1 complex to Rag GTPases in 6ces or simply due to intrinsic flexibility within DEPDC5, but the former seems more likely. Looking specifically at the impact of the larger threonine sidechain at the inter-domain interface, the Ala1395Thr variant was analysed in both 6ces and 6cet using the Missense3D server, a tool which performs *in silico* mutagenesis on the specified PDB file and then evaluates the effect of variants by a number of quantitative and qualitative criteria[103]: though the method has relatively low sensitivity, it provides high specificity for identifying structurally damaging variants which are likely to lead to loss of function. In PDB 6cesD, the variant was calculated as causing a reduction in volume of a cavity (i.e. the interdomain space around the variant) of 27.0 $Å^3$, below the threshold of 70.0 $Å^3$ which is used by Missense3D to classify variants as structurally damaging. In PDB 6cetD, the same variant was predicted to reduce the cavity volume by 74.95 $Å^3$, and thus was classified as structurally damaging. These results again suggest that the variant may have a deleterious impact on protein folding and stability, or that it impairs the ability of DEPDC5 to undergo subtle conformational changes in response to binding and – possibly – the activity state of Rag GTPases.

Supplementary Material 19: Dynamic expression of *SCN1A* and *DEPDC5*

Processed RNA-sequencing (RNA-Seq) data from the developing and adult human brain was retrieved from the BrainSpan database[30]. The complete dataset consisted of gene expression data produced across 26 brain regions and 13 age bins (8-9 post conception weeks (PCW), 12 PCW, 13 PCW, 16-17 PCW, 19-21 PCW, 24-37 PCW, 4 months, 6-12 months, 2-4 years, 8-11 years, 13-19 years, 21-37 years, and 40+ years). For the analysis of *DEPDC5* and *SCN1A* expression over time, the gene expression profiles from the inferolateral temporal cortex and the posterior (caudal) superior temporal cortex were extracted.

3. Supplementary Figures

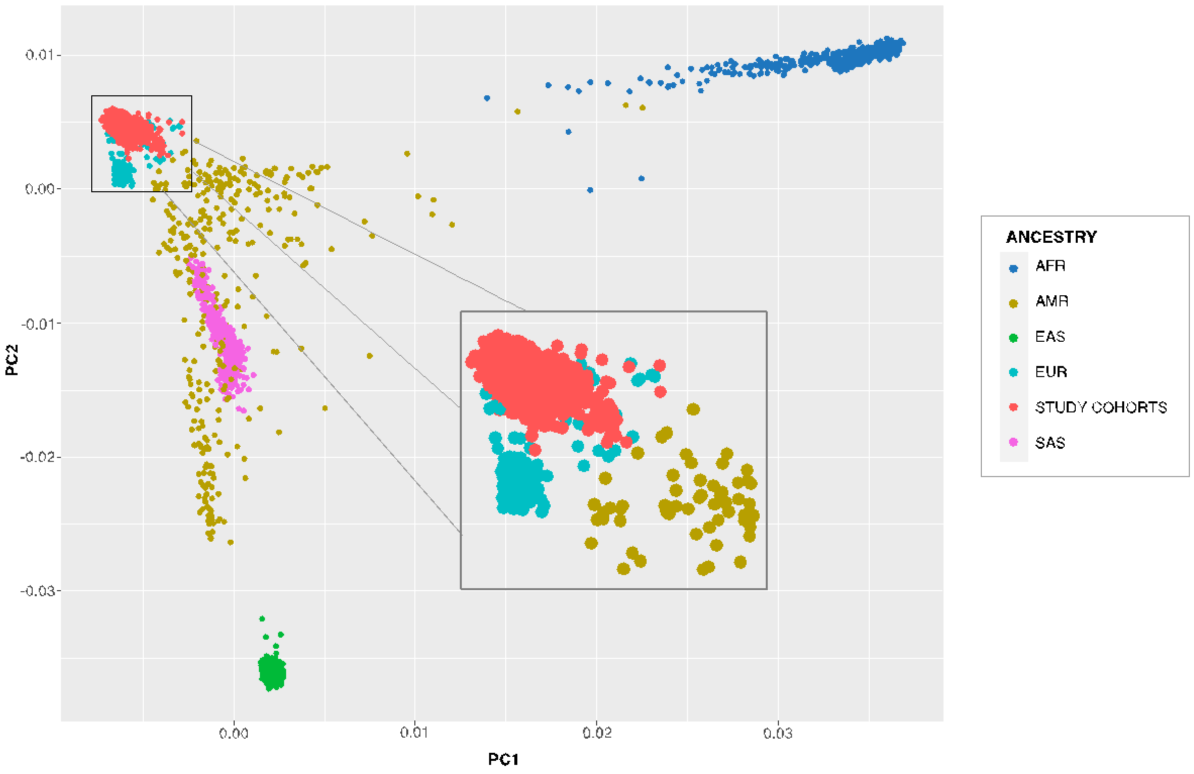

Supplementary Figure 1: Ancestry of the individuals in the study cohorts (Dravet syndrome, GEL Epilepsy controls, GEL controls and GEL *SCN1A* controls) determined by comparison with the 1000 Genomes Project reference dataset. A principal component 1 (PC1) vs principal component 2 (PC2) plot was used to visualise sample ancestry.

AFR: 1000 Genomes African samples; AMR: 1000 Genomes admixed American samples; EAS: 1000 Genomes East Asian samples; EUR: 1000 Genomes European samples; SAS: 1000 GenomesSouth Asian samples.

STUDY COHORTS: Dravet syndrome (*n*=28), GEL Epilepsy controls (*n*=772), GEL controls (*n*=1,187) and GEL *SCN1A* controls (n=45).

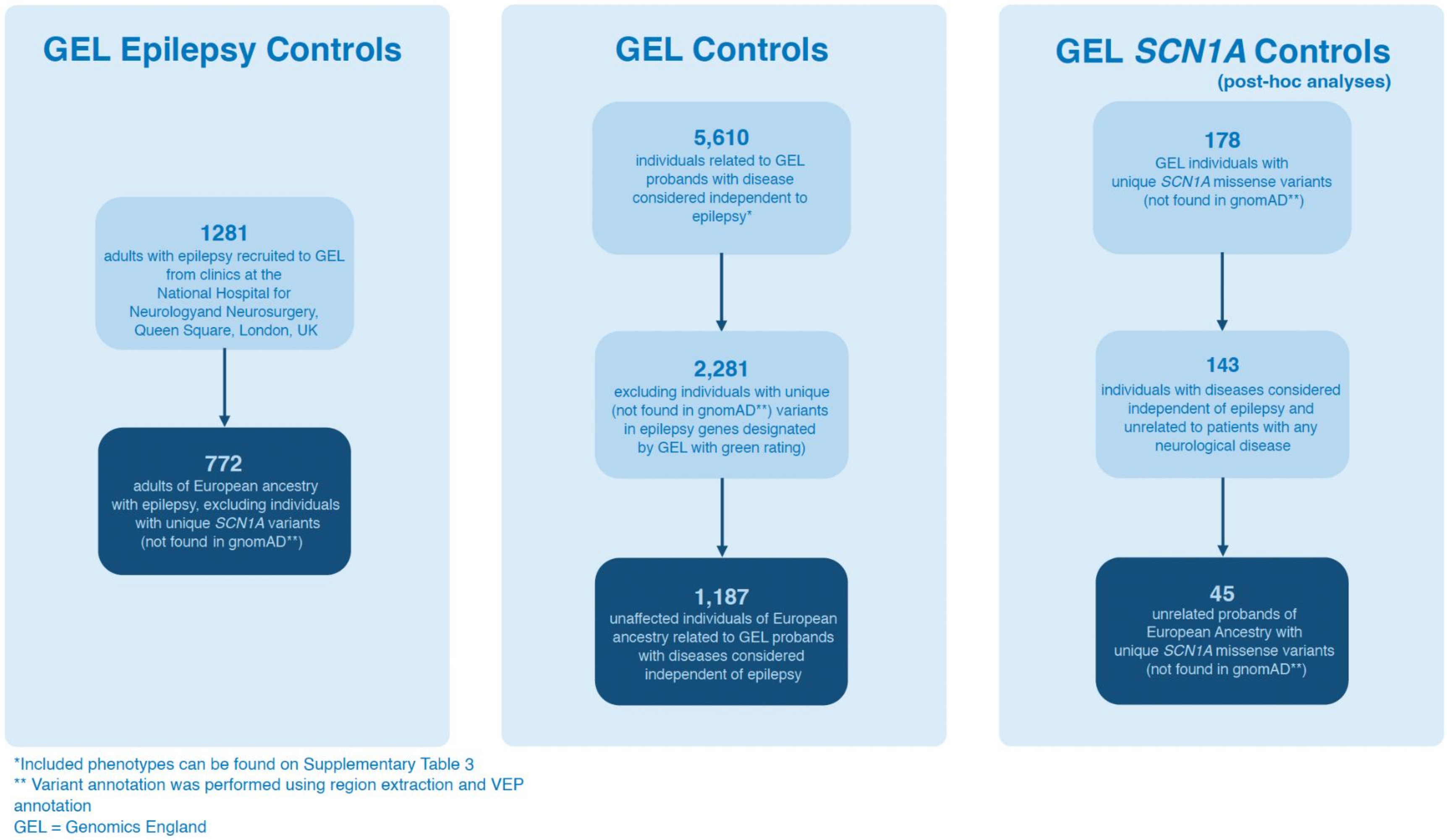

Supplementary Figure 2: PRS control cohorts. Description of control cohorts used for polygenic risk score and burden analyses.

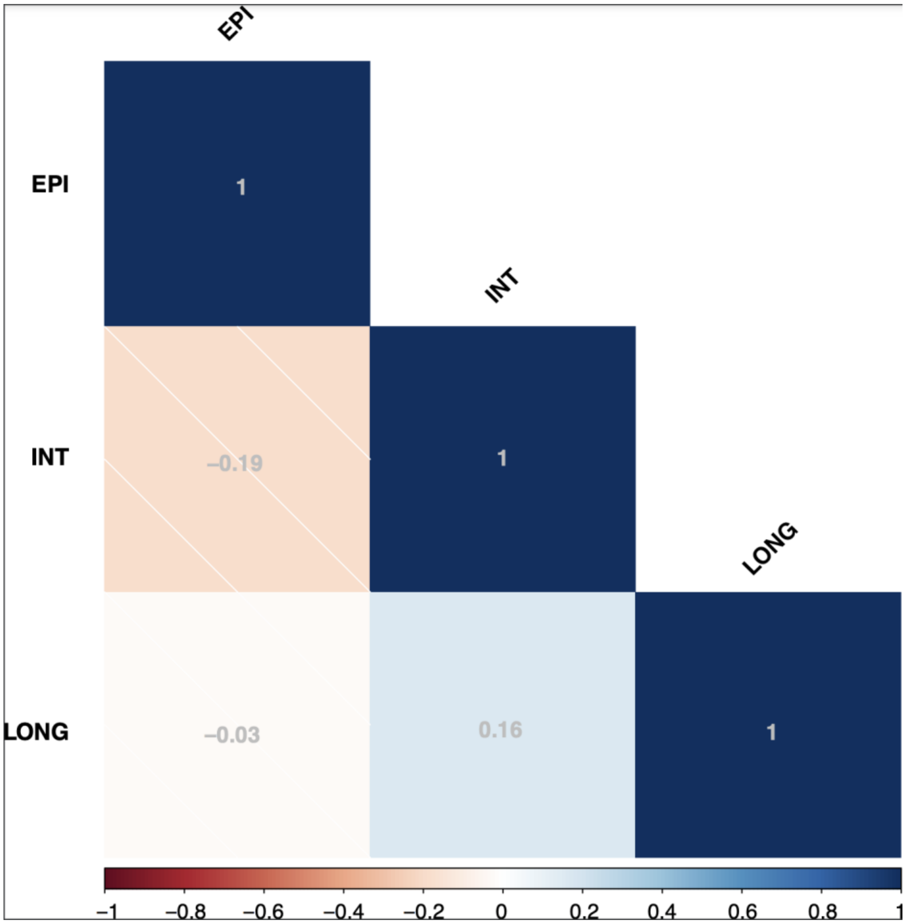

Supplementary Figure 3: **LD-score regression estimate between intelligence, longevity and epilepsy GWASes:** The genetic correlation coefficient (rg), calculated using LDSC tool[32], is denoted with a colour scale ranging from -1 (red) to 1 (blue). Intelligence and longevity are weakly genetically correlated (rg=0.16), intelligence and epilepsy show a weak negative genetic correlation (rg=-0.19), and epilepsy and longevity show negligible negative genetic correlation. Diagonal lines represent negative genetic correlation coefficients. EPI = epilepsy; INT = intelligence; LONG = longevity.

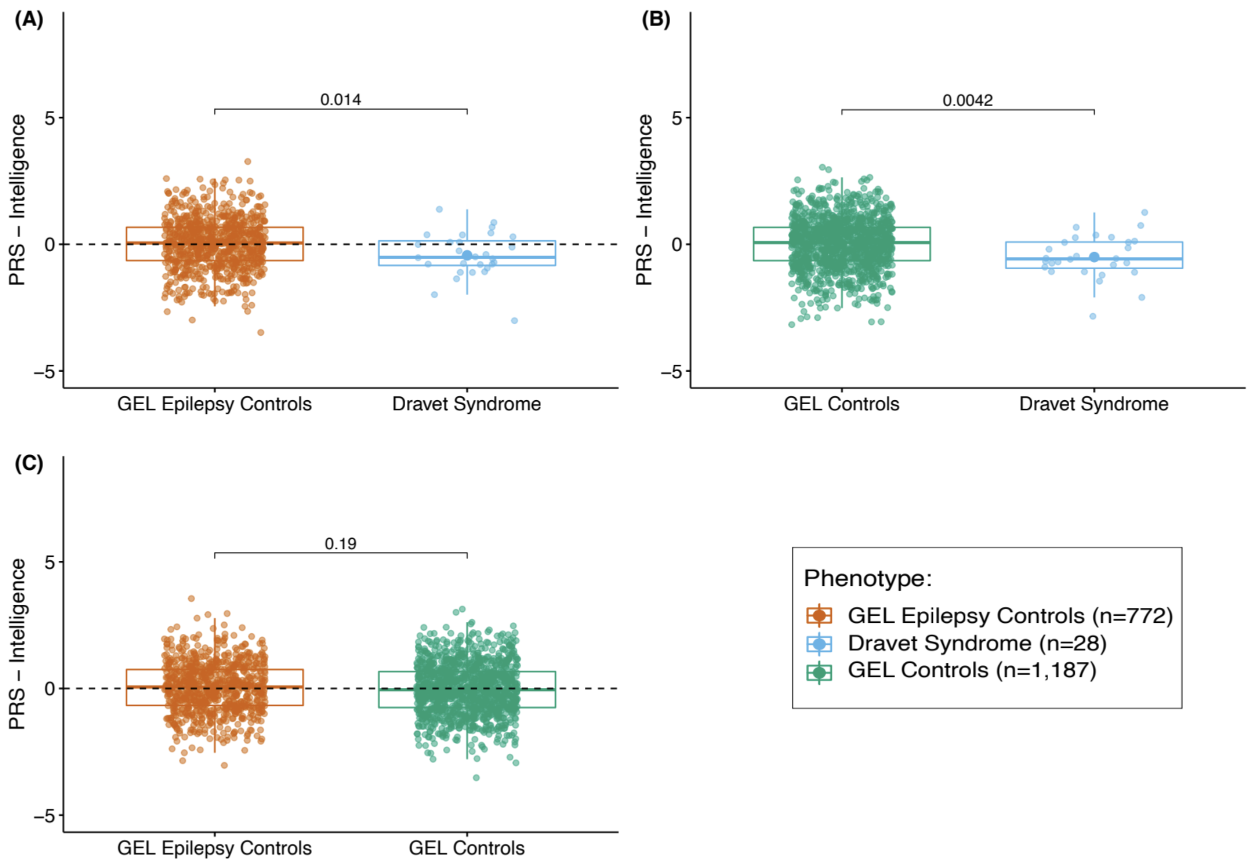

Supplementary Figure 4: Intelligence Polygenic risk score (PRS) for P-value threshold equal to 10^{-4}. (A) PRS for intelligence estimation for Dravet syndrome vs GEL Epilepsy controls: PRS for intelligence was lower in the Dravet syndrome cohort than in GEL Epilepsy controls (Adjusted P=0.014). **(B) PRS for intelligence estimation for Dravet syndrome vs GEL controls:** PRS for intelligence was higher in GEL controls than in the Dravet syndrome cohort (Adjusted P=0.0042)**,** and **(C) PRS for intelligence estimation for GEL Epilepsy controls vs GEL controls:** There was no significant difference in intelligence PRS between GEL controls and GEL Epilepsy controls (Adjusted P=0.19). The per-PRS P-values shown in the graphics are estimated using a post-hoc multiple pairwise comparison (Tukey's test). As three separate PRS analyses were performed, the adjusted P-value significance threshold was set to α=0.05/3.

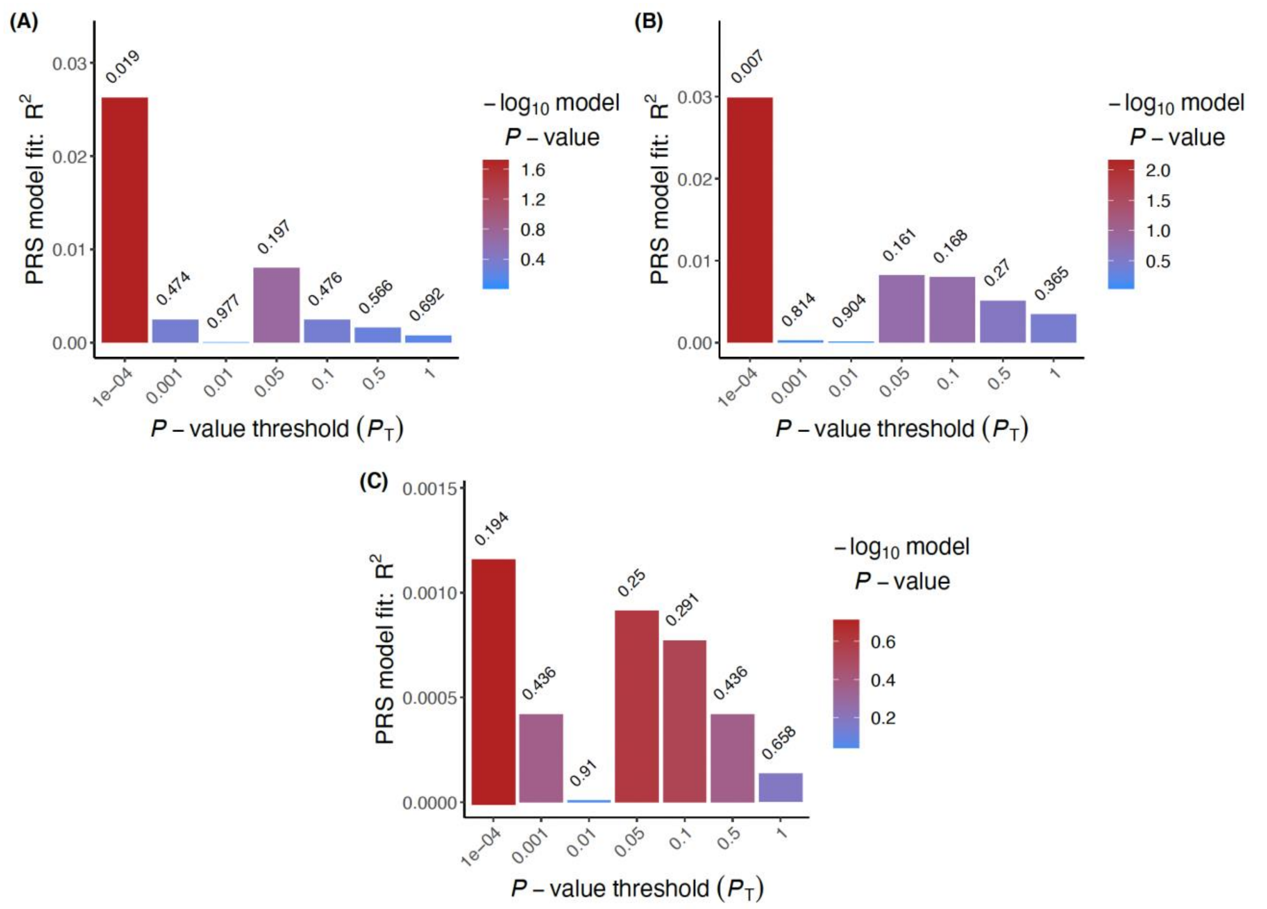

Supplementary Figure 5: Bar plot displaying the model fit of the intelligence PRS at P-value threshold: (A) Dravet syndrome cohort vs GEL Epilepsy controls, (B) Dravet syndrome cohort vs GEL controls and (C) GEL Epilepsy vs GEL controls: The model fit of the intelligence PRS shows a best predicting P-value threshold at 10^{-4} in all study models; Dravet syndrome cohort vs GEL Epilepsy controls, Dravet syndrome cohort vs GEL controls, and GEL Epilepsy vs GEL controls.

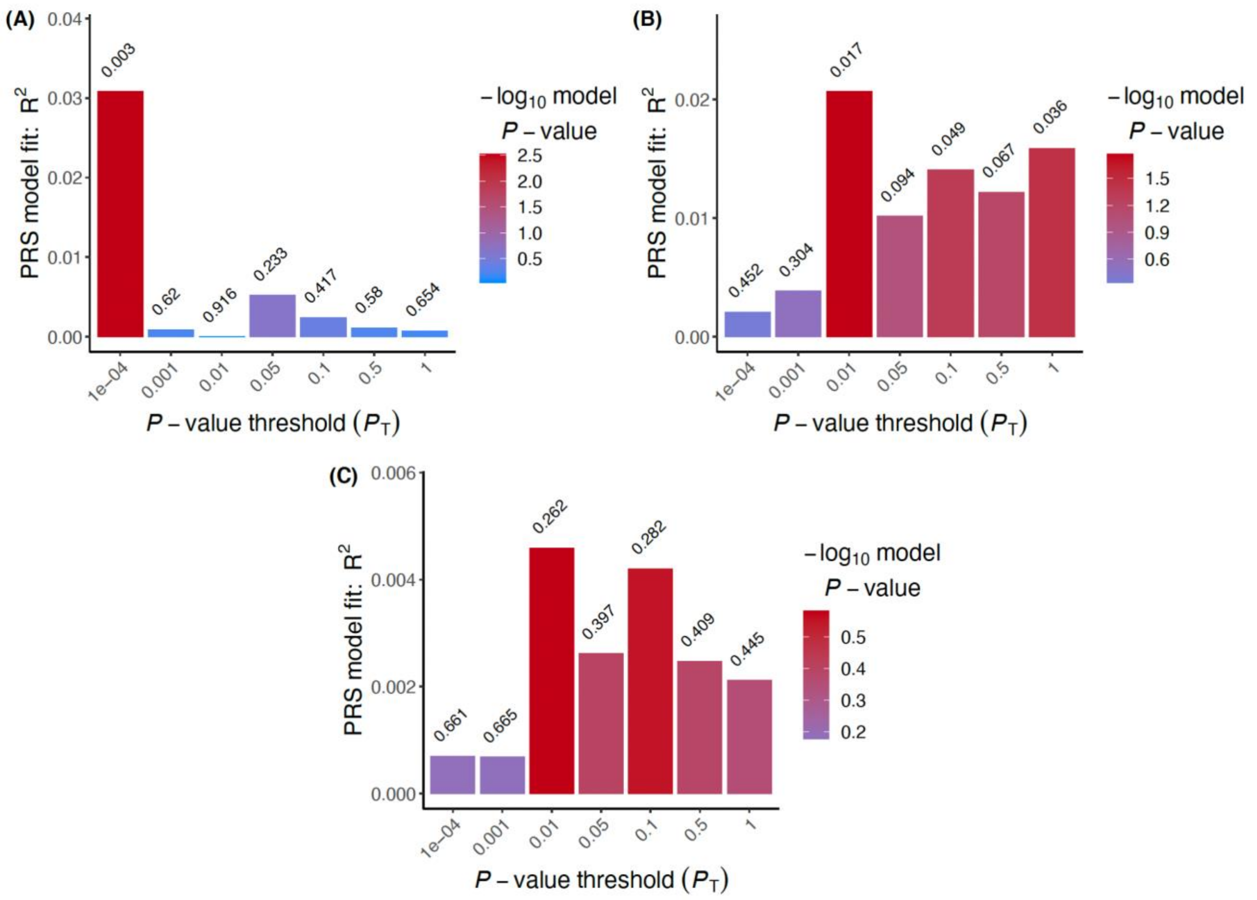

Supplementary Figure 6: P-value thresholding for polygenic risk score analysis: (A) Bar plot displaying the model fit of the intelligence PRS at P-value threshold (Dravet syndrome vs GEL Epilepsy and GEL controls): The model fit of the intelligence PRS shows a best predicting P-value threshold at 10^{-4}. The intelligence PRS explained around 3% (R^2=0.030) of the total phenotypic variance in the Dravet syndrome cohort. (**B) Bar plot displaying the model fit of the longevity PRS at P-value threshold (Dravet syndrome vs GEL Epilepsy and GEL controls):** The model fit of the longevity PRS shows a best predicting P-value threshold at 10^{-2}**.** The longevity PRS explained around 2% (R^2=0.020) of the total phenotypic variance in the Dravet syndrome cohort. (**C) Bar plot displaying the model fit of the epilepsy PRS at P-value threshold (Dravet syndrome vs GEL Epilepsy and GEL controls):** The model fit of the epilepsy PRS shows the best predicting P-value threshold at 10^{-2}**.** The epilepsy PRS explained around 0.05 % (R^2=0.0005) of the total phenotypic variance in the Dravet syndrome cohort.

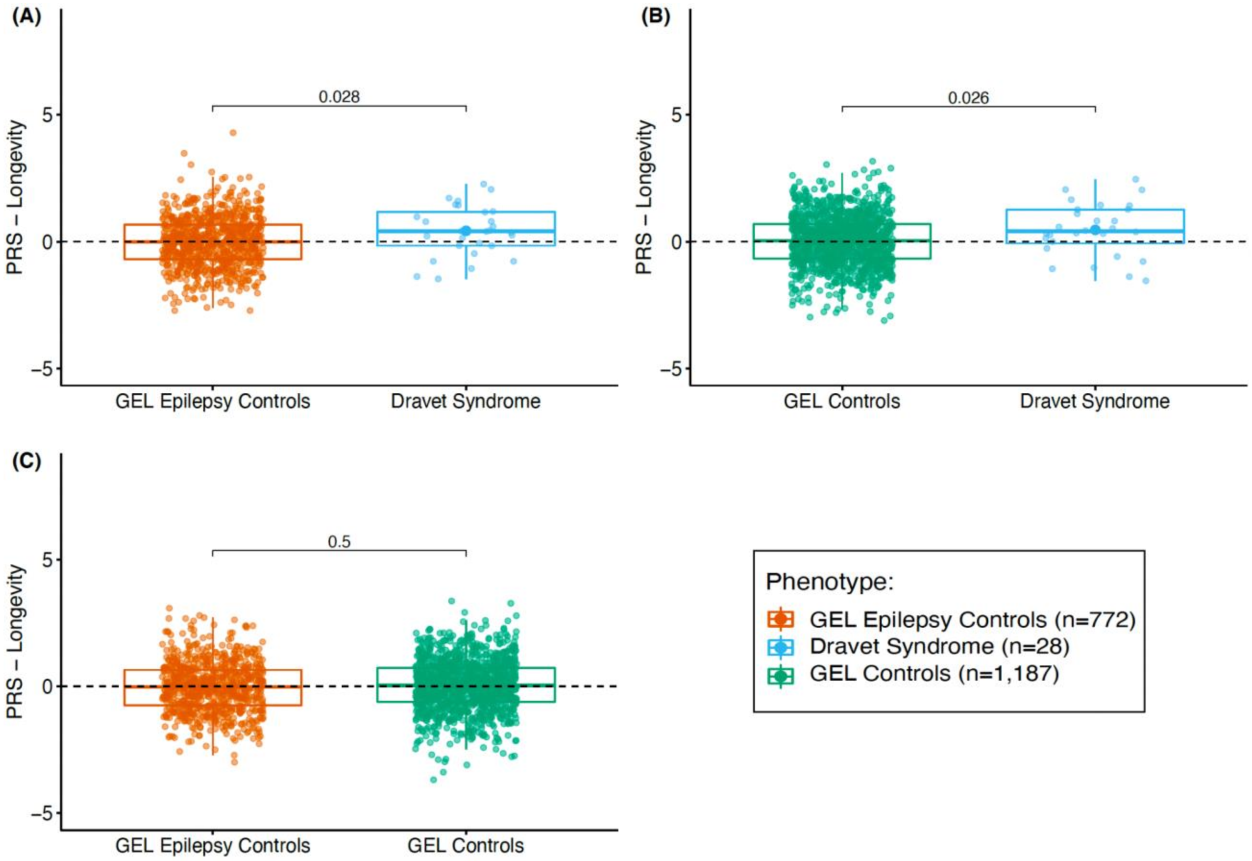

Supplementary Figure 7: Longevity Polygenic risk score (PRS) for P-value threshold equal to 10^{-4}. (A) PRS for longevity estimation for Dravet syndrome vs GEL Epilepsy controls: PRS for longevity was higher in Dravet syndrome cohort than in GEL Epilepsy controls (Adjusted P=0.028). **(B) PRS for longevity estimation for Dravet syndrome vs GEL controls:** PRS for longevity was higher in the Dravet syndrome cohort than in GEL controls (Adjusted P=0.026)**, and (C) PRS for longevity estimation for GEL Epilepsy vs GEL controls cohorts:** PRS for longevity was not significant higher in GEL controls than in GEL Epilepsy controls (Adjusted P=0.5). The per-PRS P-values shown in the graphics are estimated using a post-hoc multiple pairwise comparison (Tukey's test). As three separate PRS analyses were performed, the adjusted P-value significance threshold was set to α=0.05/3.

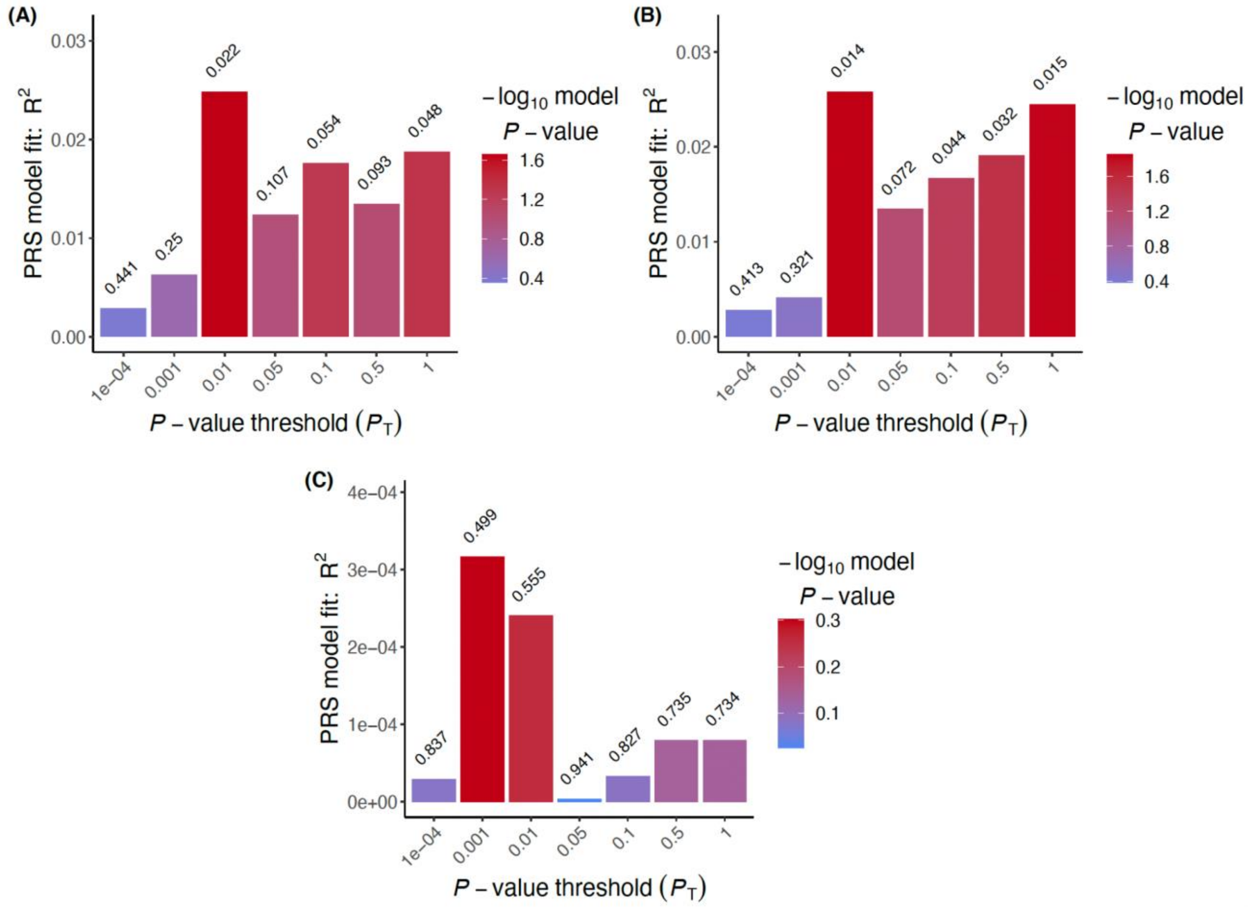

Supplementary Figure 8: Bar plot displaying the model fit of the longevity PRS at P-value threshold in the models: (A) Dravet syndrome vs GEL Epilepsy controls, (B) Dravet syndrome vs GEL controls and (C) GEL Epilepsy vs GEL controls: The model fit of the longevity PRS shows the best predicting P-value threshold at 10^{-2}.

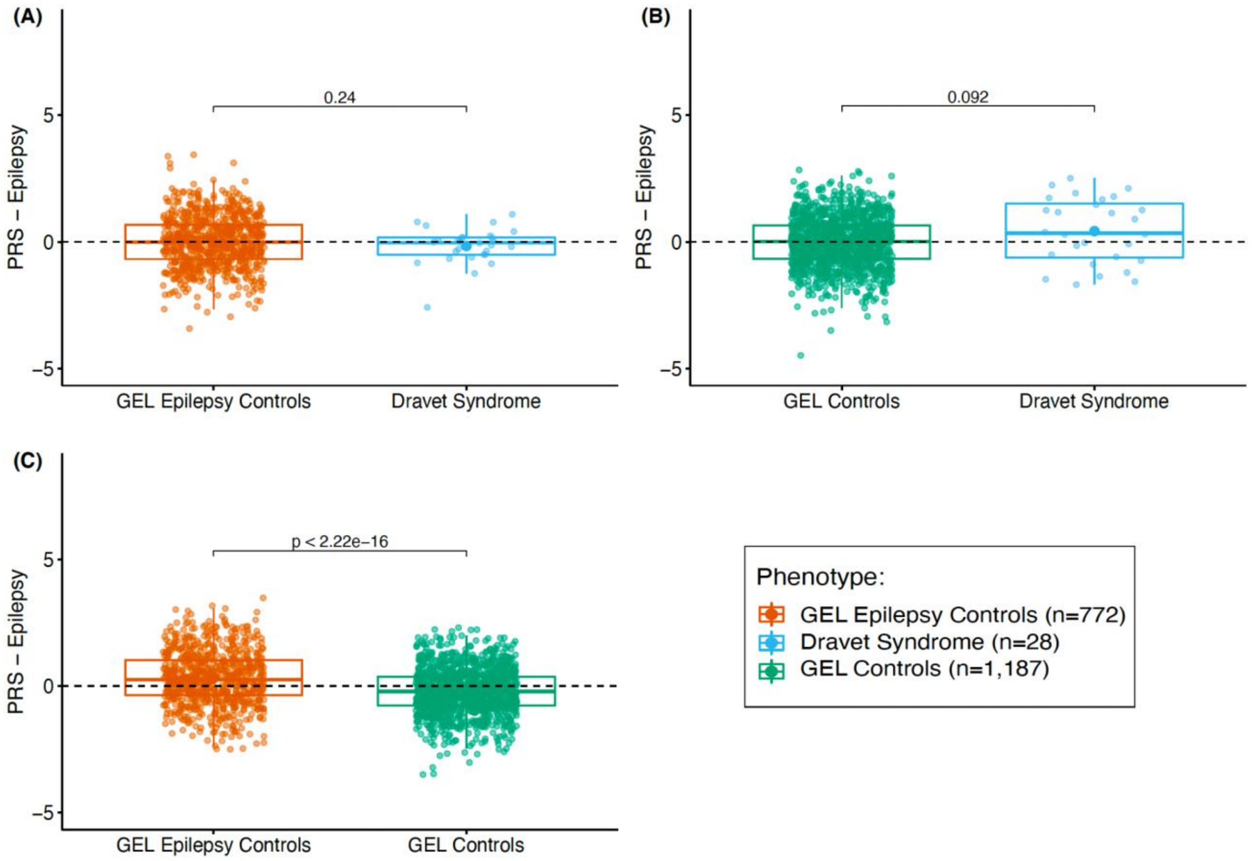

Supplementary Figure 9: Epilepsy Polygenic risk score (PRS) for P-value threshold equal to 10^{-4}. (A) PRS for epilepsy estimation for Dravet syndrome vs Epilepsy controls: There was no significant difference in the epilepsy PRS between the Dravet syndrome cohort and GEL Epilepsy controls (Adjusted P=0.24). **(B) PRS for epilepsy estimation for Dravet syndrome vs GEL controls:** There was no significant difference in the epilepsy PRS between the Dravet syndrome cohort and GEL controls (Adjusted P=0.092). **(C) PRS for epilepsy estimation for Epilepsy controls vs GEL controls:** The epilepsy PRS was significantly higher in the GEL Epilepsy controls than the GEL controls (P<2.2e-16). The per-PRS P-values shown in the graphics are estimated using a post-hoc multiple pairwise comparison (Tukey's test). As three separate PRS analyses were performed, the adjusted P-value significance threshold was set to α=0.05/3.

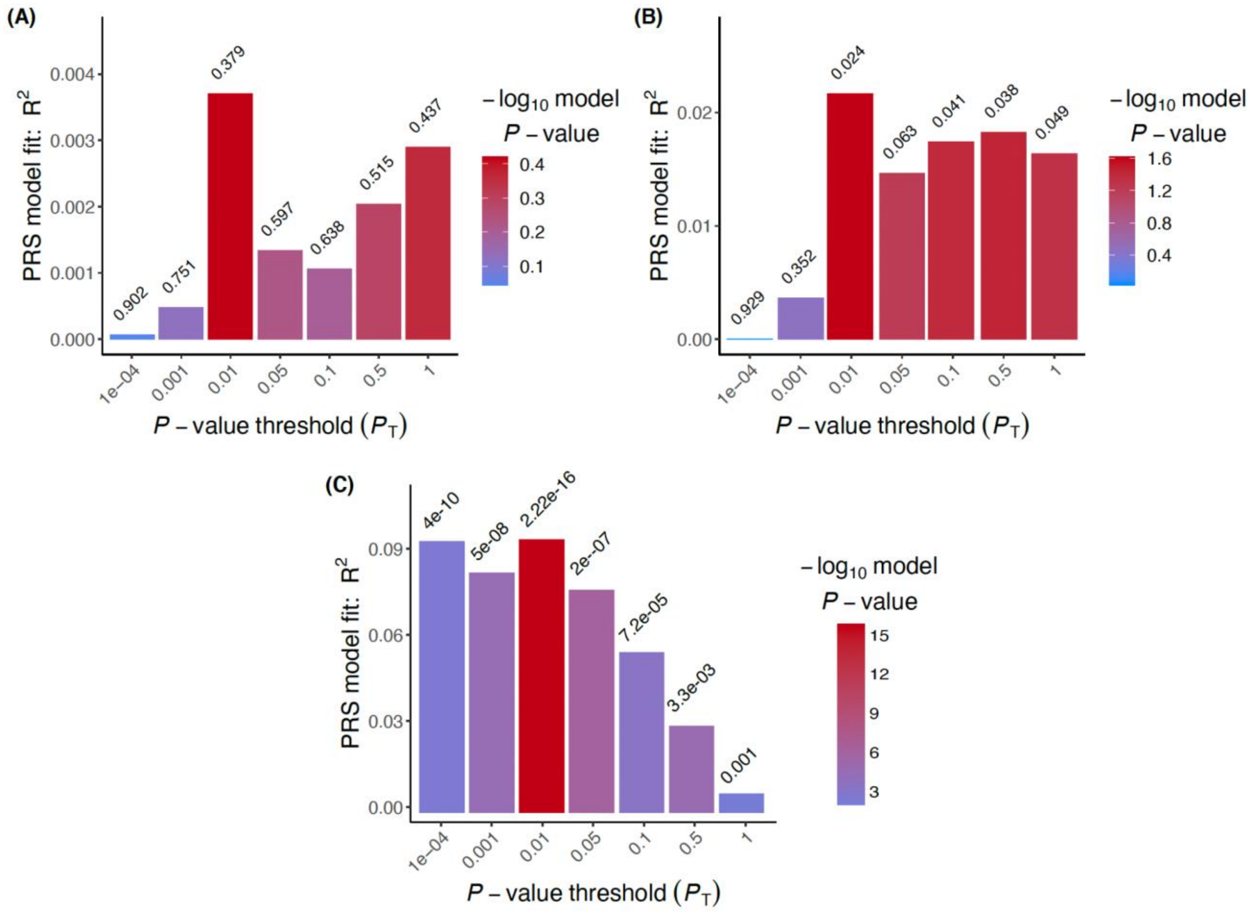

Supplementary Figure 10: Bar plot displaying the model fit of the epilepsy PRS at P-value threshold: (A) Dravet syndrome vs GEL Epilepsy controls, (B) Dravet syndrome vs GEL controls and (C) GEL Epilepsy vs GEL controls: The model fit of the longevity PRS shows a best predicting P-value threshold at 10^{-2}.

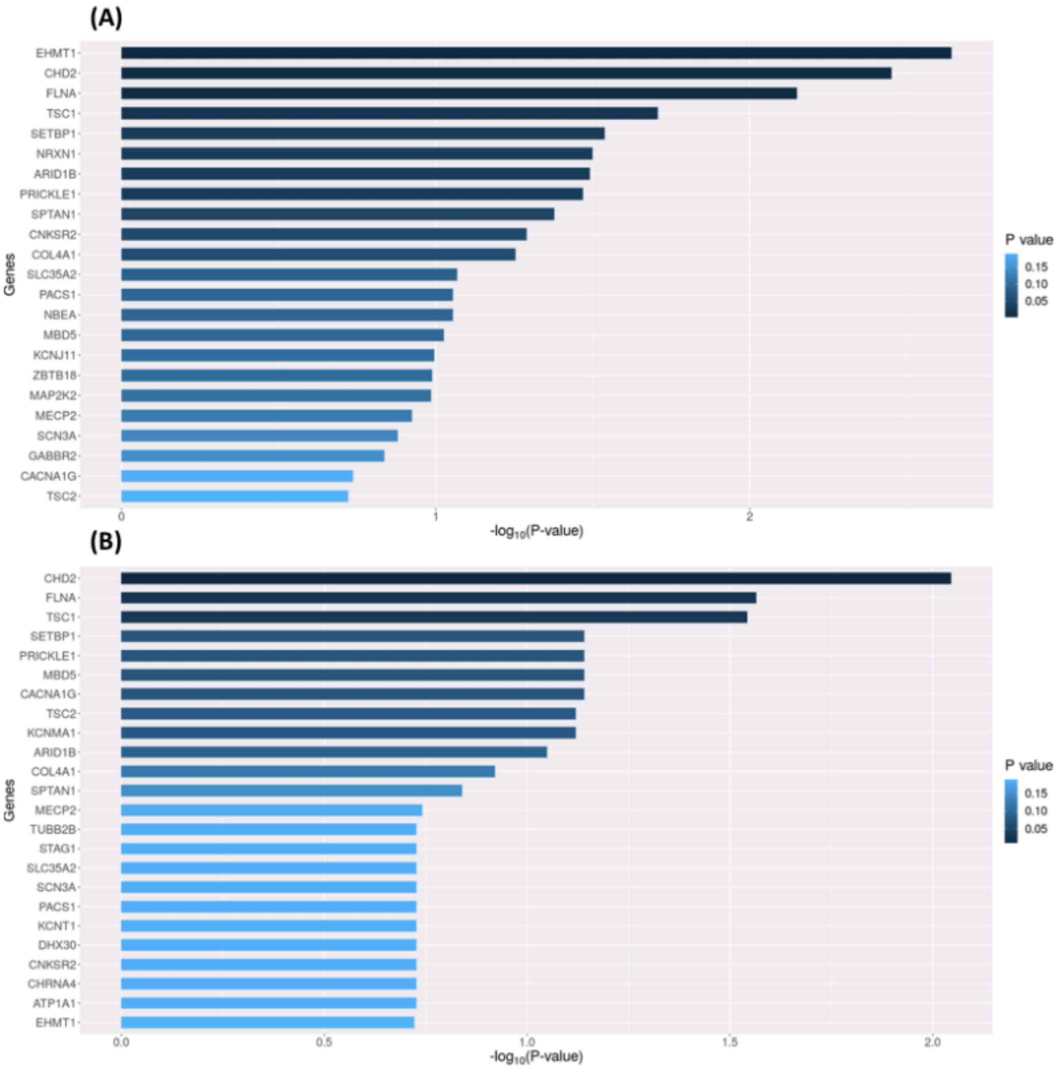

Supplementary Figure 11: Plot of P-values for gene-based collapsing analyses (SKAT-O) of rare variants across the epilepsy-related genes. (A) SKAT-O P-value of epilepsy-related genes in individuals with Dravet syndrome versus GEL Epilepsy controls. **(B)** SKAT-O P-value of epilepsy-related genes in individuals with Dravet syndrome versus GEL *SCN1A* controls.

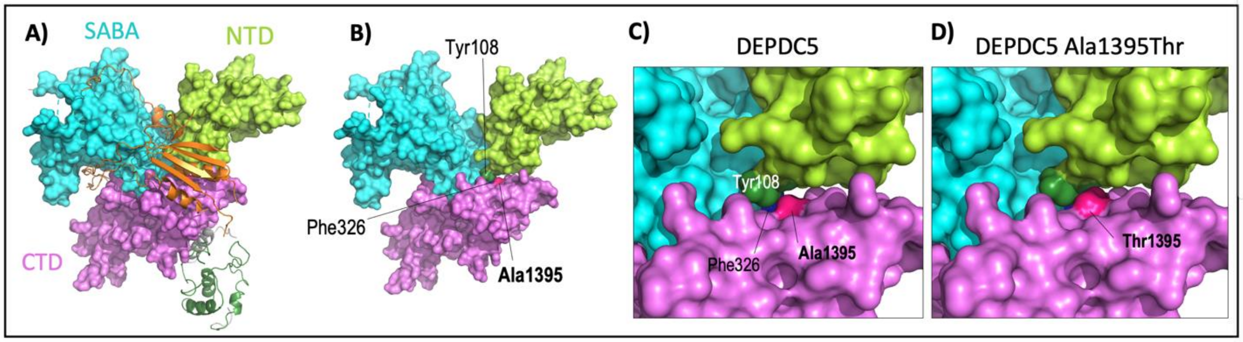

Supplementary Figure 12: (A) Ala1395 lies at an inter-domain interface in DEPDC5. The figure shows selected residues of DEPDC5 from PDB 6ces (chain D); residues of the N-terminal domain (NTD; 38-165; light green), SABA domain (166-425; cyan) and C-terminal domain (CTD; 1271-1600; violet) are shown as separate surfaces; residues of the SHEN domain (721-1010; orange) and DEP domain (1175-1270; dark green) are shown as ribbons. **(B)** shows the same structure as **(A)** with SHEN and DEP domains removed; residues Tyr108 (bright green), Phe326 (blue) and Ala1395 (rose pink) lie in close proximity at a 3-way interface between the N-terminal, SABA and C-terminal domains. (Supplementary Figures 12A and B show the same as Fig. 4E and F, main text, but show DEPDC5 from PDB 6cet (chain D)). **(C)** DEPDC5 (PDB 6ces, chain D) as in **(A)** and **(B)**, zoomed to show detail around the 3-way interface between the N-terminal, SABA and C-terminal domains in PDB 6cet chain D; **(D) The Ala1395Thr substitution results in reduced space at the inter-domain interface in 6cesD**. This figure shows the same structure as **(C)** after introduction of the Ala1395Thr variant by *in silico* mutagenesis.

Supplementary Figure 13

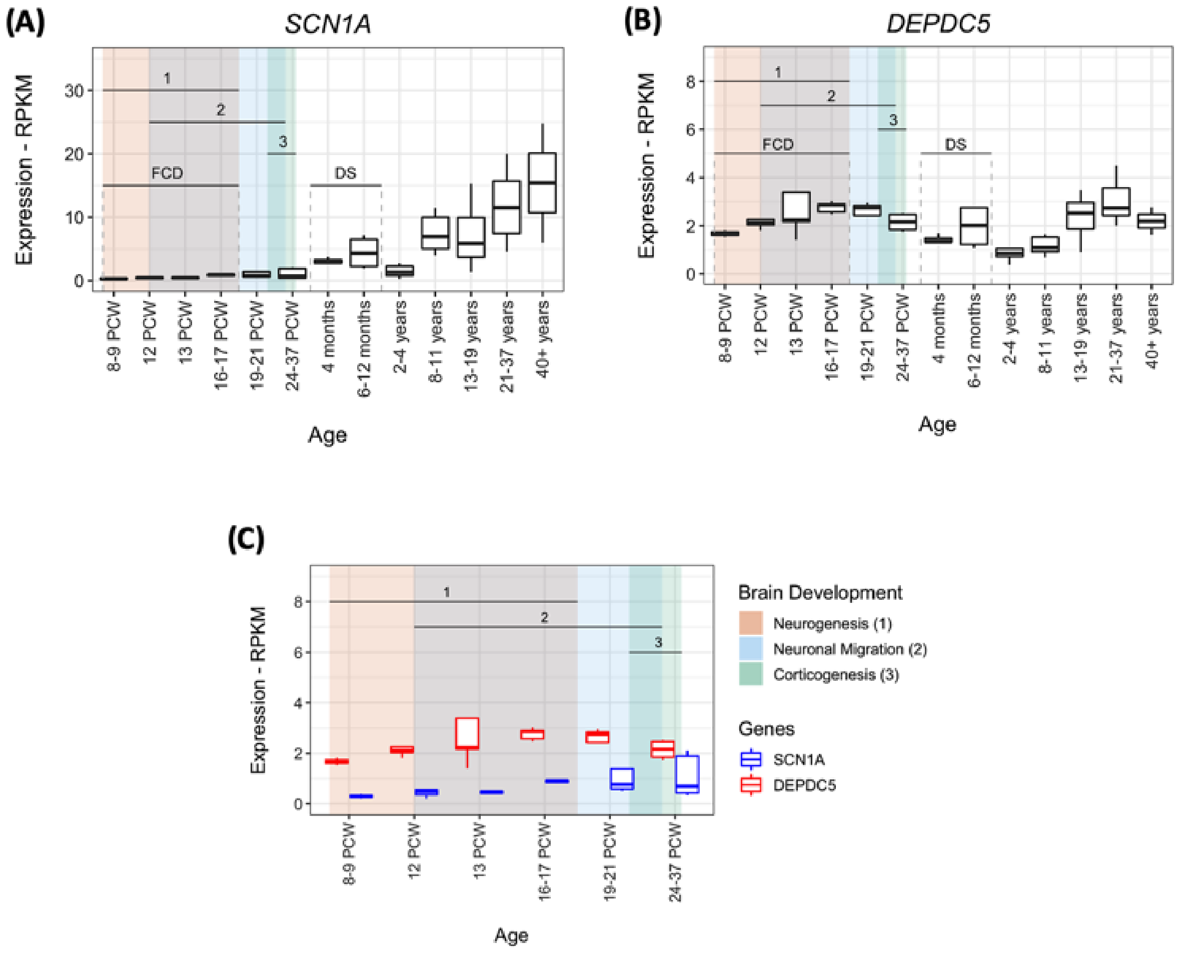

Supplementary Figure 13. Temporal expression patterns of *SCN1A* and *DEPDC5*. (A) Temporal expression pattern of *SCN1A* and **(B)** *DEPDC5*, in the inferolateral temporal cortex and the posterior (caudal) superior temporal cortex from thirteen different age bins (x-axis). Expression as Reads per Kilobase Million (RPKM) is shown on the y-axis (Supplementary Material 19). The various, overlapping, stages of brain development are highlighted in different colours, and the approximate beginning and end points of each brain development phase are depicted as horizontal lines. The development of FCD and the typical age of seizure onset in Dravet syndrome are represented as horizontal lines. *SCN1A* expression is low until 24-37 PCW, at which point expression begins to increase. This increase in expression continues throughout maturation and into adulthood. By contrast, *DEDPC5*, while having lower levels of expression overall, is

consistently expressed throughout life. (**C)** The difference between *SCN1A* and *DEPDC5* expression is most marked between 8-21 PCW, the known developmental timeline of FCD, and at which point *SCN1A* expression is minimal.

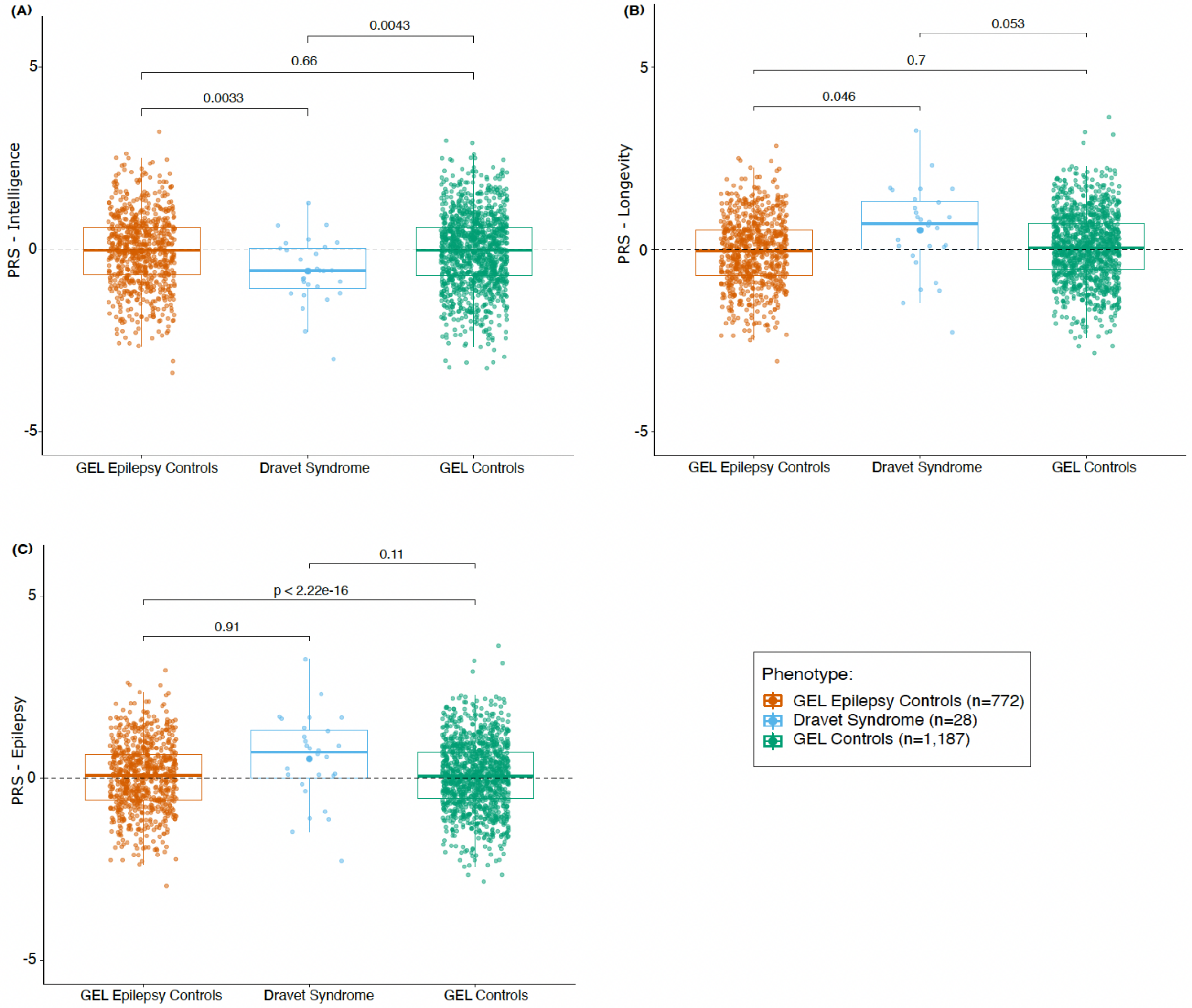

Supplementary Figure 14: Localised Polygenic Risk Score (PRS) for intelligence (A), longevity (B) and epilepsy (C), performed excluding the 2q24.3 *SCN1A*-related locus. PRS were compared across the Dravet syndrome cohort, GEL Epilepsy controls and GEL controls. Exclusion of the *SCN1A* signal did not modify the findings from the full PRS analysis, indicating that *SCN1A* is not driving the lower PRS for intelligence and the higher PRS for longevity observed in the Dravet syndrome cohort compared to the control cohorts. The per-PRS P-values shown in the graphics are estimated using a post-hoc multiple pairwise comparison (Tukey's test). As three

separate PRS analyses were performed, the adjusted P-value significance threshold was set to α=0.05/3.

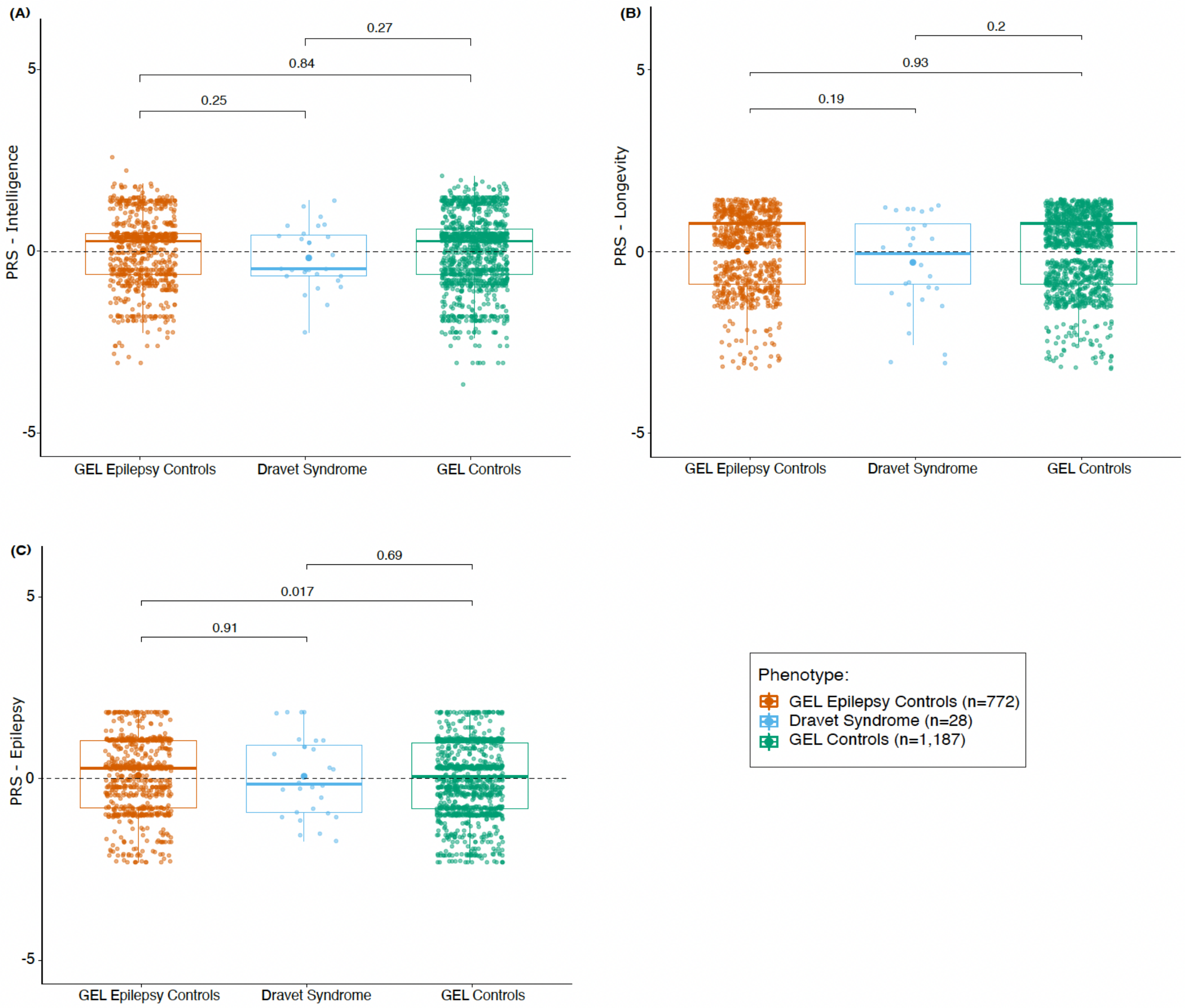

Supplementary Figure 15: Localised Polygenic Risk Score (PRS) for intelligence (A), longevity (B) and epilepsy (C), performed including only the 2q24.3 *SCN1A*-related locus. PRS were compared across the Dravet syndrome cohort, GEL Epilepsy controls and GEL controls. PRS analyses performed considering only the 2q24.3 *SCN1A*-related SNPs did not show a significant difference across the cohorts, further supporting the finding that *SCN1A* is not driving the observed differences in PRS. The per-PRS P-values shown in the graphics are estimated using a post-hoc multiple pairwise comparison (Tukey's test). As three separate PRS analyses were performed, the adjusted P-value significance threshold was set to α=0.05/3.

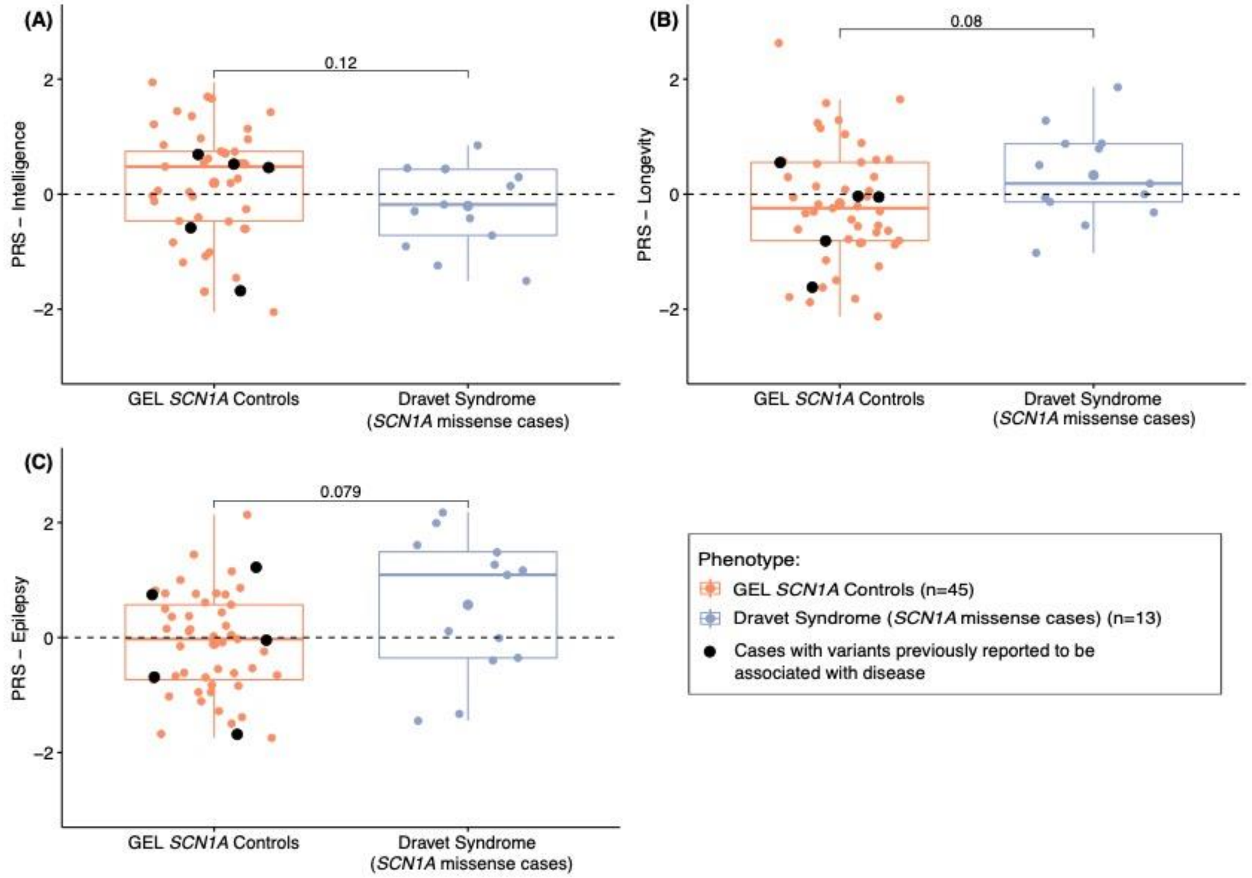

Supplementary Figure 16: Polygenic Risk Scores (PRS) applied across the GEL *SCN1A* control and Dravet syndrome cohort with *SCN1A* missense variants cohorts: (A) PRS for intelligence was lower but not significant in Dravet syndrome cases with *SCN1A* missense variants than in GEL *SCN1A* control cohort (Adjusted *P*=0.12). **(B) PRS for longevity** was higher but not significant in Dravet syndrome cases with *SCN1A* missense variants than in the GEL *SCN1A* control cohort (Adjusted *P*=0.08). **(C) PRS for epilepsy** was higher but not significant between Dravet syndrome cases with *SCN1A* missense variants and individuals in the GEL *SCN1A* control cohort. Black circles = Individuals from the GEL *SCN1A* control cohort with variants previously reported to be associated with disease. The per-PRS P-values shown in the graphics are estimated using a post-hoc multiple pairwise comparison (Tukey's test). As three separate PRS analyses were performed, the adjusted P-value significance threshold was set to α=0.05/3.

2. Supplementary Tables

Please note that Supplementary Tables 1 - 5 and 7 are provided as excel documents.

Supplementary Table 6: Phenotype information of the two unrelated individuals with Dravet syndrome who share the same *SCN1A* splicing variant.

**Case:**	**1-105287**	**1-105683**
***SCN1A* variant:**	NM_006920.6:c.602+1G>T	
**Inheritance:**	Paternal (Father mosaic)	Unknown
**Sex**	Female	Male
**Age at genetic diagnosis:**	7 years	14 years
**Age at seizure onset:**	6 months	9 months
**Seizure Type at onset:**	Prolonged hemiclonic, evolving to bilateral tonic clonic seizure	Prolonged bilateral tonic clonic seizure
**Provoking factor:**	24 hours after vaccination	No provocation
**Status epilepticus:**	Frequent in childhood	Frequent in childhood
**All seizure types experienced:**	Tonic-clonic[a], myoclonic jerks, "unresponsive episodes"[b], FIAS, hemiclonic	Tonic-clonic[a], myoclonic jerks, "unresponsive episodes"[b]
**Development:**	Normal until after seizure onset	Normal until after seizure onset
**Age at delay/regression:**	Language delay by 2.5 years	Unknown

**All antiseizure medications tried:**	Phenobarbitone, phenytoin, carbamazepine, lamotrigine, valproate	Phenytoin, carbamazepine, lamotrigine, topiramate, gabapentin
**Age at last follow-up:**	21 years	24 years
**Antiseizure medication at last follow-up:**	Stiripentol<br>Topiramate<br>Clobazam	Valproate<br>Ketogenic diet
**Seizures at most recent follow-up:**	Around 6 tonic-clonic[a] seizures per year	Weekly "unresponsive episodes"[b]<br>Weekly tonic seizures from sleep<br>Rare myoclonic jerks
**Intellectual disability:**	Mild-moderate	Severe, non-verbal
**Gait and mobility:**	Very mild crouch gait<br>Still independently mobile	Crouch gait in childhood<br>Wheelchair dependant since 11 years
**Other medical problems:**	Scoliosis<br>Anxiety<br>Difficulties with sleep	Scoliosis
**PRS epilepsy:**	-1.42	0.61
**PRS intelligence:**	0.67	-1.02
**Number of additional rare variants[c]**	2 *(KCNT1, SLC35A2)*	1 *(COL4A1)*

Two unrelated individuals with Dravet syndrome share the same *SCN1A* splicing variant. On detailed review of the individual phenotypes, there are both shared and divergent clinical features, as well as shared and divergent aspects of the clinical management. In adulthood, case 1-105287 has a milder phenotype, with relatively infrequent seizures, mild intellectual disability and is independently mobile, with only a slight crouch gait. On the other hand, case 1-105683 has weekly seizures, severe intellectual disability, is non-verbal and non-ambulant. Case 1-105287, with the milder phenotype, has a lower polygenic risk score (PRS) for epilepsy, and a higher PRS for intelligence than the more severely affected case 1-105683, suggesting a more favourable genetic background against which the *SCN1A* variant exerts its effect. Although illustrated in a small sample, these cases demonstrate, in principle, the concept that differences in genomic background underlie (at least in part) the phenotypic heterogeneity seen in "monogenic disorders"; a = unclear if generalised or focal onset; b = no EEG to confirm seizure type; c = Additional rare variants (allele frequency in gnomAD<0.0005) in epilepsy-related genes (for details see Supplementary Table 1 and 7); FIAS=focal impaired awareness seizures.

Supplementary Table 8: Individuals reported in the literature with Dravet syndrome and *SCN1A* variants who also have focal cortical dysplasia (FCD)

Patient ID in reporting paper / sex	Reported *SCN1A* variant / Inheritance	MRI brain[a]	Histology[a]	Reference
5 / Male	NM_001165963: c.C2593T:p.R865X / NR	Right temporo-anterior FCD	FCD Ib[b]	Vezyroglou 2020[104]
6 / Male	NM_001165963: c.G985T:p.G329C / NR	Left temporo-occipital FCD	FCD[b]	Vezyroglou 2020[104]
1 / Male	NM_001165963:c.602+1G>A / de novo	Increased T2 and FLAIR signal intensity and grey/white matter blurring in the right temporal lobe, right hippocampal volume loss	No specimen	Barba 2014[105]
2 / Male	NM_001165963:c.317C>T;p.S106F / paternal inheritance[d]	Increased T2 and FLAIR signal intensity and grey/white matter blurring in the right temporoccipital lobe	FCD Ia[b]	Barba 2014[105]
3 / Male	NM_001165963:c.504dupA;p.S169Ifs[c]108 / de novo	Normal	Micronodular Dysplasia[c]	Barba 2014[105]
4 / Male[e]	NM_001165963:c.2584C>G;p.R862G / de novo	Increased T2 and FLAIR intensity, grey matter/white matter blurring, cortical thickening and abnormal sulcation in the R hemisphere, bilateral PNH, cerebellar atrophy	FCD IIa[b]	Barba 2014[105]
3 / Male	p.R393C[f] / NR	Right temporal pole FCD	No specimen	Striano 2007[106] Guerrini 2011[107]
1 / Female	c.4507-4509del[f]/ de novo	Subtle temporal atrophy bilaterally	FCD IIb[a]	Wright 2015[108]

a - as reported in referenced paper;
b - Identified after surgical resection;
c - identified from post-mortem examination;
d - Father also had epilepsy (no further clinical details given);
e - Individual also underwent sequencing of *FLNA*, due to presence of PNH**;** NR = not reported in paper, PNH = periventricular nodular heterotopia, FCD = focal cortical dysplasia;
f – no further variant information provided in reporting paper

5. Supplementary References

1. Li H, Durbin R. Fast and accurate long-read alignment with Burrows-Wheeler transform. *Bioinformatics*. 2010;26(5):589-595.

2. DePristo MA, Banks E, Poplin R, et al. A framework for variation discovery and genotyping using next-generation DNA sequencing data. *Nat Genet*. 2011;43(5):491-498.

3. Zuberi SM, Wirrell E, Yozawitz E, et al. ILAE classification and definition of epilepsy syndromes with onset in neonates and infants: Position statement by the ILAE Task Force on Nosology and Definitions. *Epilepsia*. 2022;63(6):1349-1397.

4. Kwan P, Arzimanoglou A, Berg AT, et al. Definition of drug resistant epilepsy: consensus proposal by the ad hoc Task Force of the ILAE Commission on Therapeutic Strategies. *Epilepsia*. 2010;51(6):1069-1077.

5. Kobayashi K, Ohmori I, Ouchida M, Ohtsuka Y. Dravet syndrome with an exceptionally good seizure outcome in two adolescents. *Epileptic Disord*. 2011;13(3):340-344.

6. Buoni S, Orrico A, Galli L, et al. SCN1A (2528delG) novel truncating mutation with benign outcome of severe myoclonic epilepsy of infancy. *Neurology*. 2006;66(4):606-607.

7. 1000 Genomes Project Consortium, Auton A, Brooks LD, et al. A global reference for human genetic variation. *Nature*. 2015;526(7571):68-74.

8. McLaren W, Gil L, Hunt SE, et al. The Ensembl Variant Effect Predictor. *Genome Biol*. 2016;17(1):122.

9. Zuberi SM, Brunklaus A, Birch R, Reavey E, Duncan J, Forbes GH. Genotype-phenotype associations in SCN1A-related epilepsies. *Neurology*. 2011;76(7):594-600.

10. Till Á, Zima J, Fekete A, et al. Mutation spectrum of the SCN1A gene in a Hungarian population with epilepsy. *Seizure*. 2020;74:8-13.

11. Staněk D, Laššuthová P, Štěrbová K, et al. Detection rate of causal variants in severe childhood epilepsy is highest in patients with seizure onset within the first four weeks of life. *Orphanet J Rare Dis*. 2018;13(1):71.

12. Lindy AS, Stosser MB, Butler E, et al. Diagnostic outcomes for genetic testing of 70 genes in 8565 patients with epilepsy and neurodevelopmental disorders. *Epilepsia*. 2018;59(5):1062-1071.

13. Gorman KM, Peters CH, Lynch B, et al. Persistent sodium currents in SCN1A developmental and degenerative epileptic dyskinetic encephalopathy. *Brain Commun*. 2021;3(4):fcab235.

14. Koh HY, Haghighi A, Keywan C, et al. Genetic Determinants of Sudden Unexpected Death in Pediatrics. *Genet Med*. Published online January 10, 2022. doi:10.1016/j.gim.2021.12.004

15. Nykamp K, Anderson M, Powers M, et al. Sherloc: a comprehensive refinement of the ACMG-

AMP variant classification criteria. *Genet Med*. 2017;19(10):1105-1117.

16. Kumar P, Henikoff S, Ng PC. Predicting the effects of coding non-synonymous variants on protein function using the SIFT algorithm. *Nat Protoc*. 2009;4(7):1073-1081.

17. Adzhubei IA, Schmidt S, Peshkin L, et al. A method and server for predicting damaging missense mutations. *Nat Methods*. 2010;7(4):248-249.

18. Schwarz JM, Rödelsperger C, Schuelke M, Seelow D. MutationTaster evaluates disease-causing potential of sequence alterations. *Nat Methods*. 2010;7(8):575-576.

19. Jaganathan K, Kyriazopoulou Panagiotopoulou S, McRae JF, et al. Predicting Splicing from Primary Sequence with Deep Learning. *Cell*. 2019;176(3):535-548.e24.

20. Choi SW, Mak TSH, O'Reilly PF. Tutorial: a guide to performing polygenic risk score analyses. *Nat Protoc*. 2020;15(9):2759-2772.

21. Savage JE, Jansen PR, Stringer S, et al. Genome-wide association meta-analysis in 269,867 individuals identifies new genetic and functional links to intelligence. *Nat Genet*. 2018;50(7):912-919.

22. Deelen J, Evans DS, Arking DE, et al. A meta-analysis of genome-wide association studies identifies multiple longevity genes. *Nat Commun*. 2019;10(1):3669.

23. Purcell S, Neale B, Todd-Brown K, et al. PLINK: a tool set for whole-genome association and population-based linkage analyses. *Am J Hum Genet*. 2007;81(3):559-575.

24. Choi SW, O'Reilly PF. PRSice-2: Polygenic Risk Score software for biobank-scale data. *Gigascience*. 2019;8(7). doi:10.1093/gigascience/giz082

25. Braatz V, Martins Custodio H, Leu C, et al. Postictal Psychosis in Epilepsy: A Clinicogenetic Study. *Ann Neurol*. 2021;90(3):464-476.

26. de Lange IM, Koudijs MJ, van 't Slot R, et al. Mosaicism of de novo pathogenic SCN1A variants in epilepsy is a frequent phenomenon that correlates with variable phenotypes. *Epilepsia*. 2018;59(3):690-703.

27. Martin AR, Williams E, Foulger RE, et al. PanelApp crowdsources expert knowledge to establish consensus diagnostic gene panels. *Nat Genet*. 2019;51(11):1560-1565.

28. 100,000 Genomes Project Pilot Investigators, Smedley D, Smith KR, et al. 100,000 Genomes Pilot on Rare-Disease Diagnosis in Health Care - Preliminary Report. *N Engl J Med*. 2021;385(20):1868-1880.

29. Zhao Z, Bi W, Zhou W, VandeHaar P, Fritsche LG, Lee S. UK Biobank Whole-Exome Sequence Binary Phenome Analysis with Robust Region-Based Rare-Variant Test. *Am J Hum Genet*. 2020;106(1):3-12.

30. Miller JA, Ding SL, Sunkin SM, et al. Transcriptional landscape of the prenatal human brain. *Nature*. 2014;508(7495):199-206.

31. International League Against Epilepsy Consortium on Complex Epilepsies. Genome-wide mega-

analysis identifies 16 loci and highlights diverse biological mechanisms in the common epilepsies. *Nat Commun*. 2018;9(1):5269.

32. Bulik-Sullivan B, Finucane HK, Anttila V, et al. An atlas of genetic correlations across human diseases and traits. *Nat Genet*. 2015;47(11):1236-1241.

33. Brunklaus A, Ellis R, Stewart H, et al. Homozygous mutations in the SCN1A gene associated with genetic epilepsy with febrile seizures plus and Dravet syndrome in 2 families. *Eur J Paediatr Neurol*. 2015;19(4):484-488.

34. UniProt: the Universal Protein knowledgebase in 2023. *Nucleic Acids Res*. 2023;51(D1):D523-D531.

35. Omasits U, Ahrens CH, Müller S, Wollscheid B. Protter: interactive protein feature visualization and integration with experimental proteomic data. *Bioinformatics*. 2014;30(6):884-886.

36. Lamar KMJ, Carvill GL. Chromatin Remodeling Proteins in Epilepsy: Lessons From CHD2-Associated Epilepsy. *Front Mol Neurosci*. 2018;11:208.

37. Liu JC, Ferreira CG, Yusufzai T. Human CHD2 is a chromatin assembly ATPase regulated by its chromo- and DNA-binding domains. *J Biol Chem*. 2015;290(1):25-34.

38. De Maria B, Balestrini S, Mei D, et al. Expanding the genetic and phenotypic spectrum of CHD2-related disease: From early neurodevelopmental disorders to adult-onset epilepsy. *Am J Med Genet A*. 2022;188(2):522-533.

39. Chen J, Zhang J, Liu A, et al. CHD2-related epilepsy: novel mutations and new phenotypes. *Dev Med Child Neurol*. 2020;62(5):647-653.

40. Silvennoinen K, Martins Custodio H, Balestrini S, Rugg-Gunn F, England Research Consortium G, Sisodiya SM. Complex epilepsy: it's all in the history. *Pract Neurol*. Published online October 17, 2020. doi:10.1136/practneurol-2020-002522

41. Brunklaus A, Ellis R, Reavey E, Forbes GH, Zuberi SM. Prognostic, clinical and demographic features in SCN1A mutation-positive Dravet syndrome. *Brain*. 2012;135(Pt 8):2329-2336.

42. Li W, Schneider AL, Scheffer IE. Defining Dravet syndrome: An essential pre-requisite for precision medicine trials. *Epilepsia*. 2021;62(9):2205-2217.

43. Zhu L, Peng F, Deng Z, Feng Z, Ma X. A Novel Variant of the CHD2 Gene Associated With Developmental Delay and Myoclonic Epilepsy. *Front Genet*. 2022;13:761178.

44. Jansen FE, Sadleir LG, Harkin LA, et al. Severe myoclonic epilepsy of infancy (Dravet syndrome): recognition and diagnosis in adults. *Neurology*. 2006;67(12):2224-2226.

45. Akiyama M, Kobayashi K, Yoshinaga H, Ohtsuka Y. A long-term follow-up study of Dravet syndrome up to adulthood. *Epilepsia*. 2010;51(6):1043-1052.

46. Takayama R, Fujiwara T, Shigematsu H, et al. Long-term course of Dravet syndrome: a study from an epilepsy center in Japan. *Epilepsia*. 2014;55(4):528-538.

47. Genton P, Velizarova R, Dravet C. Dravet syndrome: the long-term outcome. *Epilepsia*. 2011;52

Suppl 2:44-49.

48. Catarino CB, Liu JYW, Liagkouras I, et al. Dravet syndrome as epileptic encephalopathy: evidence from long-term course and neuropathology. *Brain*. 2011;134(Pt 10):2982-3010.

49. Dravet C. The core Dravet syndrome phenotype. *Epilepsia*. 2011;52 Suppl 2:3-9.

50. Yakoub M, Dulac O, Jambaqué I, Chiron C, Plouin P. Early diagnosis of severe myoclonic epilepsy in infancy. *Brain Dev*. 1992;14(5):299-303.

51. Carvill GL, Heavin SB, Yendle SC, et al. Targeted resequencing in epileptic encephalopathies identifies de novo mutations in CHD2 and SYNGAP1. *Nat Genet*. 2013;45(7):825-830.

52. Thomas RH, Zhang LM, Carvill GL, et al. CHD2 myoclonic encephalopathy is frequently associated with self-induced seizures. *Neurology*. 2015;84(9):951-958.

53. Suls A, Jaehn JA, Kecskés A, et al. De novo loss-of-function mutations in CHD2 cause a fever-sensitive myoclonic epileptic encephalopathy sharing features with Dravet syndrome. *Am J Hum Genet*. 2013;93(5):967-975.

54. Kim YJ, Khoshkhoo S, Frankowski JC, et al. Chd2 Is Necessary for Neural Circuit Development and Long-Term Memory. *Neuron*. 2018;100(5):1180-1193.e6.

55. Meganathan K, Lewis EMA, Gontarz P, et al. Regulatory networks specifying cortical interneurons from human embryonic stem cells reveal roles for CHD2 in interneuron development. *Proc Natl Acad Sci U S A*. 2017;114(52):E11180-E11189.

56. Trivisano M, Striano P, Sartorelli J, et al. CHD2 mutations are a rare cause of generalized epilepsy with myoclonic-atonic seizures. *Epilepsy Behav*. 2015;51:53-56.

57. Lund C, Brodtkorb E, Øye AM, Røsby O, Selmer KK. CHD2 mutations in Lennox-Gastaut syndrome. *Epilepsy Behav*. 2014;33:18-21.

58. Chénier S, Yoon G, Argiropoulos B, et al. CHD2 haploinsufficiency is associated with developmental delay, intellectual disability, epilepsy and neurobehavioural problems. *J Neurodev Disord*. 2014;6(1):9.

59. Monlong J, Girard SL, Meloche C, et al. Global characterization of copy number variants in epilepsy patients from whole genome sequencing. *PLoS Genet*. 2018;14(4):e1007285.

60. Galizia EC, Myers CT, Leu C, et al. CHD2 variants are a risk factor for photosensitivity in epilepsy. *Brain*. 2015;138(Pt 5):1198-1207.

61. Shoubridge C, Harvey RJ, Dudding-Byth T. IQSEC2 mutation update and review of the female-specific phenotype spectrum including intellectual disability and epilepsy. *Hum Mutat*. 2019;40(1):5-24.

62. Mignot C, McMahon AC, Bar C, et al. IQSEC2-related encephalopathy in males and females: a comparative study including 37 novel patients. *Genet Med*. 2019;21(4):837-849.

63. Shoubridge C, Tarpey PS, Abidi F, et al. Mutations in the guanine nucleotide exchange factor gene IQSEC2 cause nonsyndromic intellectual disability. *Nat Genet*. 2010;42(6):486-488.

64. Levy NS, Umanah GKE, Rogers EJ, Jada R, Lache O, Levy AP. IQSEC2-Associated Intellectual Disability and Autism. *Int J Mol Sci*. 2019;20(12). doi:10.3390/ijms20123038

65. Kalscheuer VM, James VM, Himelright ML, et al. Novel Missense Mutation A789V in IQSEC2 Underlies X-Linked Intellectual Disability in the MRX78 Family. *Front Mol Neurosci*. 2015;8:85.

66. Karaca E, Harel T, Pehlivan D, et al. Genes that Affect Brain Structure and Function Identified by Rare Variant Analyses of Mendelian Neurologic Disease. *Neuron*. 2015;88(3):499-513.

67. Piton A, Gauthier J, Hamdan FF, et al. Systematic resequencing of X-chromosome synaptic genes in autism spectrum disorder and schizophrenia. *Mol Psychiatry*. 2011;16(8):867-880.

68. Hu H, Haas SA, Chelly J, et al. X-exome sequencing of 405 unresolved families identifies seven novel intellectual disability genes. *Mol Psychiatry*. 2016;21(1):133-148.

69. Jansson JS, Hallböök T, Reilly C. Intellectual functioning and behavior in Dravet syndrome: A systematic review. *Epilepsy Behav*. 2020;108:107079.

70. de Lange IM, Gunning B, Sonsma ACM, et al. Outcomes and comorbidities of SCN1A-related seizure disorders. *Epilepsy Behav*. 2019;90:252-259.

71. Berkvens JJL, Veugen I, Veendrick-Meekes MJBM, et al. Autism and behavior in adult patients with Dravet syndrome (DS). *Epilepsy Behav*. 2015;47:11-16.

72. Ouss L, Leunen D, Laschet J, et al. Autism spectrum disorder and cognitive profile in children with Dravet syndrome: Delineation of a specific phenotype. *Epilepsia Open*. 2019;4(1):40-53.

73. Barrie ES, Cottrell CE, Gastier-Foster J, et al. Genotype-phenotype correlation: Inheritance and variant-type infer pathogenicity in IQSEC2 gene. *Eur J Med Genet*. 2020;63(3):103735.

74. Zerem A, Haginoya K, Lev D, et al. The molecular and phenotypic spectrum of IQSEC2-related epilepsy. *Epilepsia*. 2016;57(11):1858-1869.

75. Chieffo D, Battaglia D, Lucibello S, et al. Disorders of early language development in Dravet syndrome. *Epilepsy Behav*. 2016;54:30-33.

76. Ragona F, Granata T, Dalla Bernardina B, et al. Cognitive development in Dravet syndrome: a retrospective, multicenter study of 26 patients. *Epilepsia*. 2011;52(2):386-392.

77. Johannesen KM, Liu Y, Koko M, et al. Genotype-phenotype correlations in SCN8A-related disorders reveal prognostic and therapeutic implications. *Brain*. Published online August 25, 2021. doi:10.1093/brain/awab321

78. Gardella E, Møller RS. Phenotypic and genetic spectrum of SCN8A-related disorders, treatment options, and outcomes. *Epilepsia*. 2019;60 Suppl 3:S77-S85.

79. de Lera Ruiz M, Kraus RL. Voltage-Gated Sodium Channels: Structure, Function, Pharmacology, and Clinical Indications. *J Med Chem*. 2015;58(18):7093-7118.

80. Marban E, Yamagishi T, Tomaselli GF. Structure and function of voltage-gated sodium channels. *J Physiol*. 1998;508 (Pt 3):647-657.

81. Rolvien T, Butscheidt S, Jeschke A, et al. Severe bone loss and multiple fractures in SCN8A-related epileptic encephalopathy. *Bone*. 2017;103:136-143.

82. Encinas AC, Moore IKM, Watkins JC, Hammer MF. Influence of age at seizure onset on the acquisition of neurodevelopmental skills in an SCN8A cohort. *Epilepsia*. 2019;60(8):1711-1720.

83. Johannesen KM, Gardella E, Encinas AC, et al. The spectrum of intermediate SCN8A-related epilepsy. *Epilepsia*. 2019;60(5):830-844.

84. Jain P. Novel SCN8A mutation in a girl with refractory seizures and autistic features. *Neurol India*. 2017;65(1):180-181.

85. Sadleir LG, Mountier EI, Gill D, et al. Not all SCN1A epileptic encephalopathies are Dravet syndrome: Early profound Thr226Met phenotype. *Neurology*. 2017;89(10):1035-1042.

86. Knupp KG, Scarbro S, Wilkening G, Juarez-Colunga E, Kempe A, Dempsey A. Parental Perception of Comorbidities in Children With Dravet Syndrome. *Pediatr Neurol*. 2017;76:60-65.

87. Nabbout R, Mistry A, Zuberi S, et al. Fenfluramine for Treatment-Resistant Seizures in Patients With Dravet Syndrome Receiving Stiripentol-Inclusive Regimens: A Randomized Clinical Trial. *JAMA Neurol*. 2020;77(3):300-308.

88. Villas N, Meskis MA, Goodliffe S. Dravet syndrome: Characteristics, comorbidities, and caregiver concerns. *Epilepsy Behav*. 2017;74:81-86.

89. Kröll-Seger J, Portilla P, Dulac O, Chiron C. Topiramate in the treatment of highly refractory patients with Dravet syndrome. *Neuropediatrics*. 2006;37(6):325-329.

90. Buck ML, Goodkin HP. Stiripentol: A Novel Antiseizure Medication for the Management of Dravet Syndrome. *Ann Pharmacother*. 2019;53(11):1136-1144.

91. Holland KD, Bouley TM, Horn PS. Location: A surrogate for personalized treatment of sodium channelopathies. *Ann Neurol*. 2018;84(1):1-9.

92. Wagnon JL, Mencacci NE, Barker BS, et al. Partial loss-of-function of sodium channel SCN8A in familial isolated myoclonus. *Hum Mutat*. 2018;39(7):965-969.

93. Blanchard MG, Willemsen MH, Walker JB, et al. De novo gain-of-function and loss-of-function mutations of SCN8A in patients with intellectual disabilities and epilepsy. *J Med Genet*. 2015;52(5):330-337.

94. Wagnon JL, Barker BS, Ottolini M, et al. Loss-of-function variants of SCN8A in intellectual disability without seizures. *Neurol Genet*. 2017;3(4):e170.

95. Heyne HO, Baez-Nieto D, Iqbal S, et al. Predicting functional effects of missense variants in voltage-gated sodium and calcium channels. *Sci Transl Med*. 2020;12(556). doi:10.1126/scitranslmed.aay6848

96. Deciphering Developmental Disorders Study. Large-scale discovery of novel genetic causes of developmental disorders. *Nature*. 2015;519(7542):223-228.

97. Larsen J, Carvill GL, Gardella E, et al. The phenotypic spectrum of SCN8A encephalopathy.

Neurology. 2015;84(5):480-489.

98. de Kovel CGF, Meisler MH, Brilstra EH, et al. Characterization of a de novo SCN8A mutation in a patient with epileptic encephalopathy. *Epilepsy Res*. 2014;108(9):1511-1518.

99. Gardella E, Marini C, Trivisano M, et al. The phenotype of SCN8A developmental and epileptic encephalopathy. *Neurology*. 2018;91(12):e1112-e1124.

100. Meisler MH, Hill SF, Yu W. Sodium channelopathies in neurodevelopmental disorders. *Nat Rev Neurosci*. 2021;22(3):152-166.

101. Martin MS, Tang B, Papale LA, Yu FH, Catterall WA, Escayg A. The voltage-gated sodium channel Scn8a is a genetic modifier of severe myoclonic epilepsy of infancy. *Hum Mol Genet*. 2007;16(23):2892-2899.

102. Lenk GM, Jafar-Nejad P, Hill SF, et al. Scn8a Antisense Oligonucleotide Is Protective in Mouse Models of SCN8A Encephalopathy and Dravet Syndrome. *Ann Neurol*. 2020;87(3):339-346.

103. Ittisoponpisan S, Islam SA, Khanna T, Alhuzimi E, David A, Sternberg MJE. Can Predicted Protein 3D Structures Provide Reliable Insights into whether Missense Variants Are Disease Associated? *J Mol Biol*. 2019;431(11):2197-2212.

104. Vezyroglou A, Varadkar S, Bast T, et al. Focal epilepsy in SCN1A-mutation carrying patients: is there a role for epilepsy surgery? *Dev Med Child Neurol*. 2020;62(11):1331-1335.

105. Barba C, Parrini E, Coras R, et al. Co-occurring malformations of cortical development and SCN1A gene mutations. *Epilepsia*. 2014;55(7):1009-1019.

106. Striano P, Mancardi MM, Biancheri R, et al. Brain MRI findings in severe myoclonic epilepsy in infancy and genotype-phenotype correlations. *Epilepsia*. 2007;48(6):1092-1096.

107. Guerrini R, Striano P, Catarino C, Sisodiya SM. Neuroimaging and neuropathology of Dravet syndrome. *Epilepsia*. 2011;52 Suppl 2:30-34.

108. Wright R, Hernandez A, Malik S, Donahue D, Alles A, Perry M. Dravet syndrome associated with cortical dysplasia of Taylor-type. *J Pediatr Neurol*. 2015;09(04):479-481.
