# Supplementary material for: Widespread genomic influences on phenotype in Dravet syndrome, a ‘monogenic’ condition
Source: Brain. 2023 Apr 3;146(9):3885–97. doi: 10.1093/brain/awad111 (PMC10473570; doi:10.1093/brain/awad111)
Supplement: awad111_Supplementary_Data [file awad111_supplementary_data.zip › brain-2022-01408-File014.pdf]

Genomics England Research Consortium contributors

Ambrose, J. C.[1] Arumugam, P.[1], Bevers, R.[1], Bleda, M.[1], Boardman-Pretty, F.[1,2], Boustred, C. R. [1], Brittain, H.[1], Brown, M.A., Caulfield, M. J.[1,2], Chan, G. C. [1], Fowler, T. [1], Giess A. [1], Hamblin, A.[1], Henderson, S.[1,2], Hubbard, T. J. P. [1], Jackson, R. [1], Jones, L. J. [1,2], Kasperaviciute, D. [1,2], Kayikci, M. [1], Kousathanas, A. [1], Lahnstein, L. [1], Leigh, S. E. A. [1], Leong, I. U. S. [1], Lopez, F. J. [1], Maleady-Crowe, F. [1], McEntagart, M.[1], Minneci F. [1], Moutsianas, L. [1,2], Mueller, M. [1,2], Murugaesu, N. [1], Need, A. C. [1,2], O'Donovan P. [1], Odhams, C. A. [1], Patch, C. [1,2], Perez-Gil, D. [1], Pereira, M. B.[1], Pullinger, J. [1], Rahim, T. [1], Rendon, A. [1], Rogers, T. [1], Savage, K. [1], Sawant, K. [1], Scott, R. H. [1], Siddiq, A. [1], Sieghart, A. [1], Smith, S. C. [1], Sosinsky, A. [1,2], Stuckey, A. [1], Tanguy M. [1], Taylor Tavares, A. L.[1], Thomas, E. R. A. [1,2], Thompson, S. R. [1], Tucci, A. [1,2], Welland, M. J. [1], Williams, E. [1], Witkowska, K. [1,2], Wood, S. M. [1,2]

(1) Genomics England, London, UK. (2) William Harvey Research Institute, Queen Mary University of London, London, EC1M 6BQ, UK.
